# From dual norepinephrine–dopamine reuptake inhibition to selective dopamine reuptake inhibitors: a case of aromatically substituted *N*,2-cyclopentylamines

**DOI:** 10.1039/d6md00112b

**Published:** 2026-06-24

**Authors:** Majlen A. Dilweg, Willem Jespers, Ameya S. Kasture, Rongfang Liu, Tamara A. M. Mocking, Jelle G. van der Ploeg, Adrianus M. M. Baselier, Gerard J. P. van Westen, Thomas Hummel, Sonja Sucic, Adriaan P. IJzerman, Laura H. Heitman, Daan van der Es

**Affiliations:** a Division of Medicinal Chemistry, Leiden Academic Centre for Drug Research, Leiden University Leiden The Netherlands d.van.der.es@lacdr.leidenuniv.nl; b Institute of Pharmacology, Medical University of Vienna Vienna Austria; c Department of Neurosciences and Developmental Biology, University of Vienna Vienna Austria; d Oncode Institute Leiden The Netherlands; e Department of Medicinal Chemistry, Photopharmacology and Imaging, Groningen Research Institute of Pharmacy (GRIP), Faculty of Science and Engineering Antonius Deusinglaan 1 9713 AV Groningen The Netherlands

## Abstract

Dopamine (DA) is essential for motor control, mood, motivation, and reward in the central nervous system. It functions through receptor activation and is regulated by the dopamine transporter (DAT, SLC6A3), which manages DA reuptake. DAT dysfunction can lead to abnormal DA levels, associated with conditions like depression, Parkinson's disease, and substance abuse. Current efforts to develop dopamine reuptake inhibitors (DRIs) have resulted in only one approved drug, emphasizing the need for new DRI chemotypes. To address this, we expanded the structure–activity relationship of our previously reported *N*,2-substituted cycloalkylamine scaffold. We synthesized and characterized two aliphatic (9a and 10b) and sixteen aromatic (11a–11p) *N*-substituted derivatives. Pharmacological evaluation with a fluorescent neurotransmitter uptake assay revealed that compounds 9a, 10b, and a previously reported compound (6) acted as dual norepinephrine–dopamine reuptake inhibitors. Contrarily, most aromatic compounds were selective for DAT, with *meta*-hydroxyl 11e and *para*-hydroxyl 11f as most potent DRIs with an inhibitory potency in the nanomolar range. Using molecular docking, key interactions with residues R85 and D476 were proposed to underlie potent inhibitory effects. The stabilization of residue F326 within the flexible loop region between TM6a and TM6b was predicted to contribute to DAT selectivity. *In vivo* characterization using hDAT-expressing *Drosophila melanogaster* showed increased locomotor activity and reduced sleep for *para*-hydroxyl compound 11f. This study introduces a new series of potent *N*,2-cyclopentylamine-based DRIs, providing insights into mechanisms of selectivity and offering potential directions for DAT-related drug discovery.

## Abstract 2

A novel series of *N*,2-cyclopentylamine-based dopamine reuptake inhibitors was developed from earlier dual inhibitors. MAT selectivity was assessed *in vitro* and rationalized *in silico*. *In vivo* efficacy was shown in fruit fly locomotor assays.

## Graphical abstract

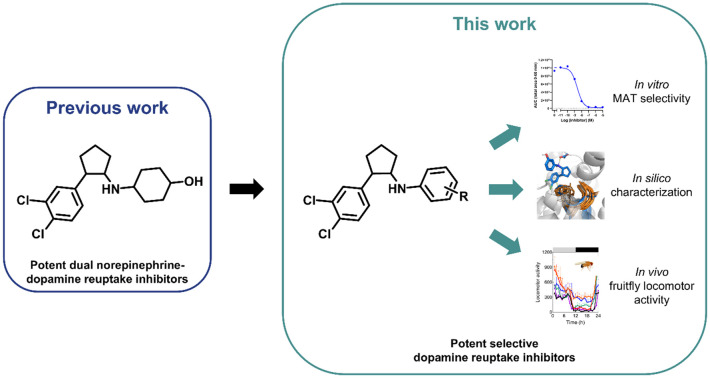

## 1. Introduction

Dopamine (DA) is a neurotransmitter that regulates vital functions such as motor control, reward processing, mood regulation, and motivation in the central nervous system (CNS).^1^ Upon release of DA *via* exocytosis from the pre-synaptic neuron, dopamine receptors can be activated to initiate intracellular signaling in the post-synaptic neuron.^2^ Embedded within the outer membrane of dopaminergic neurons, the dopamine transporter (DAT), encoded by the SLC6A3 gene, is one of the key players regulating DA levels in the synaptic cleft by reuptake of DA back into the pre-synaptic neurons.^3,4^ Disruptions in DAT function can cause excessive or deficient DA levels, which both are associated with various diseases. Depression is often associated with low DA levels, leading to symptoms such as anhedonia, low mood, and fatigue.^5^ In Parkinson's disease (PD), the loss of dopaminergic neurons results in motor deficits and cognitive decline.^6^ In contrast, substance abuse involves increased DA activity, often due to altered DAT function, reinforcing addictive behaviors.^7^

**Fig. 1:**
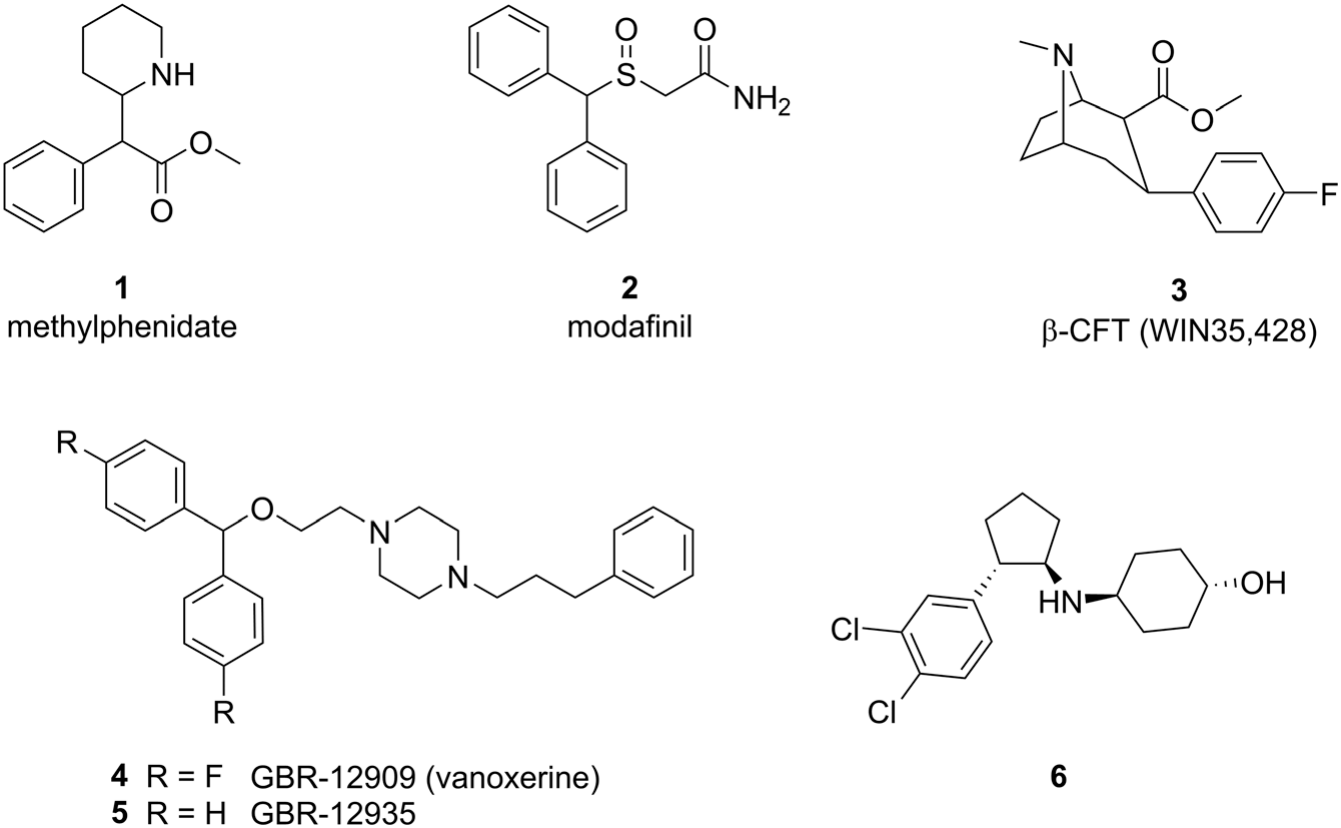
Molecular structures of NDRI methylphenidate (1), DRIs modafinil (2), β-CFT (3), GBR-12909 (4) and GBR-12935 (5), and previously identified NET inhibitor *N*,2-substituted cyclopentylamine 6.

Several pharmacological strategies can be employed to modulate DA levels. Triple reuptake inhibitors (TRIs) are able to inhibit DAT in combination with inhibition of the other monoamine transporters (MATs): the norepinephrine transporter (NET, SLC6A2) and serotonin transporter (SERT, SLC6A4).^8,9^ In addition, dual targeting inhibitors such as norepinephrine–dopamine reuptake inhibitors (NDRIs) are commonly used to treat attention deficit hyperactivity disorder (ADHD), with main examples such as methylphenidate (1, Fig. 1).^10^ In case of selective dopamine reuptake inhibitors (DRIs), multiple inhibitors have been developed with the aim to treat psychostimulant use disorder or alleviate motor-related symptoms in Parkinson's disease which are a result of dopaminergic neurodegeneration.^7,11^ Despite the various efforts in DRI development, to date only modafinil (2, Fig. 1), a moderately potent FDA-approved DRI (DAT IC_50_ value <10 μM), is currently on the market to treat narcolepsy but is used off-label for ADHD and in a combination therapy for bipolar disorder.^12–16^

Most DRIs investigated so far have been based on a few key chemical scaffolds. The most investigated chemotypes, phenyltropane and benztropine, include the tropane moiety originally derived from cocaine which is present in compounds such as β-CFT (3, Fig. 1).^17^ Phenyltropanes and benztropines have been under investigation for various therapeutic purposes and are considered typical DRIs, stabilizing the outward-facing open conformation of the transporter as demonstrated by the recently published cryo-EM structures.^18–20^ On the other hand, modafinil and related compounds are based on the benzhydryl scaffold, which are a class of atypical DRIs with non-psychostimulant properties and reduced addiction potential compared to many tropane-based DRIs.^21,22^ Disubstituted piperazine derivatives such as GBR-12909, otherwise known as vanoxerine (4, Fig. 1), and GBR-12935 (5, Fig. 1), have been reported to potently and selectively inhibit DAT with some showing promise as cocaine addiction treatments.^23^ Unlike phenyltropanes, GBR-12909 was found to stabilize DAT in the inward-facing conformation which might explain the diminished locomotor activity and abuse effects compared to cocaine.^20^ Despite drug discovery efforts using the multiple different available DRI scaffolds, there is still a high demand for new inhibitors to combat conditions like substance abuse and may also provide new therapeutic opportunities in neurodegenerative diseases such as PD and Alzheimer's disease.^11^

Previous research by our group has identified the *N*,2-substituted cycloalkylamine scaffold as a potent chemotype to inhibit NET.^24,25^ In this article the follow-up investigation of *N*,2-substituted cyclopentylamines on selective DA reuptake inhibition is reported. Therefore, we developed a new series of compounds based on 6 (Fig. 1), a previously reported *N*,2-substituted cyclopentylamine with nanomolar NET inhibitory potency, including *N*-phenyl-substituted derivatives to explore MAT selectivity.^25^ The compounds were screened for their inhibitory properties on NET, DAT and SERT with the use of a fluorescent uptake assay in order to draft a SAR as well as a selectivity profile. Subsequently, the molecular interactions underlying DAT inhibition as well as selectivity over NET and SERT were investigated using molecular docking. Additionally, the *in vivo* effects of DAT inhibition by our most potent compounds were assessed in locomotor activity experiments with *Drosophila melanogaster* expressing human DAT (hDAT). The SAR described in this study for the *N*-phenyl-substituted cyclopentylamines on the different MATs provides new insights for tuning selectivity towards DAT. This may provide new opportunities in drug discovery efforts for CNS-related disorders by using this atypical MAT inhibitor scaffold.

## 2. Results and discussion

### 2.1 Compound design and synthesis

Based on the previously described SAR of *N*,2-substituted cycloalkylamines, we designed 18 new inhibitors. Firstly, the influence of the aliphatic hydroxyl group of compound 6 was investigated with the use of tetrahydro-2*H*-pyran and cyclohexan-1-one derivatives 9a and 10b, respectively. Furthermore, to determine the possibility of including a second aromatic ring in the scaffold, reducing the number of chiral centers as well as providing more options for substitution, several substituted phenyl derivatives were introduced (11a–11p).

**Scheme 1:**
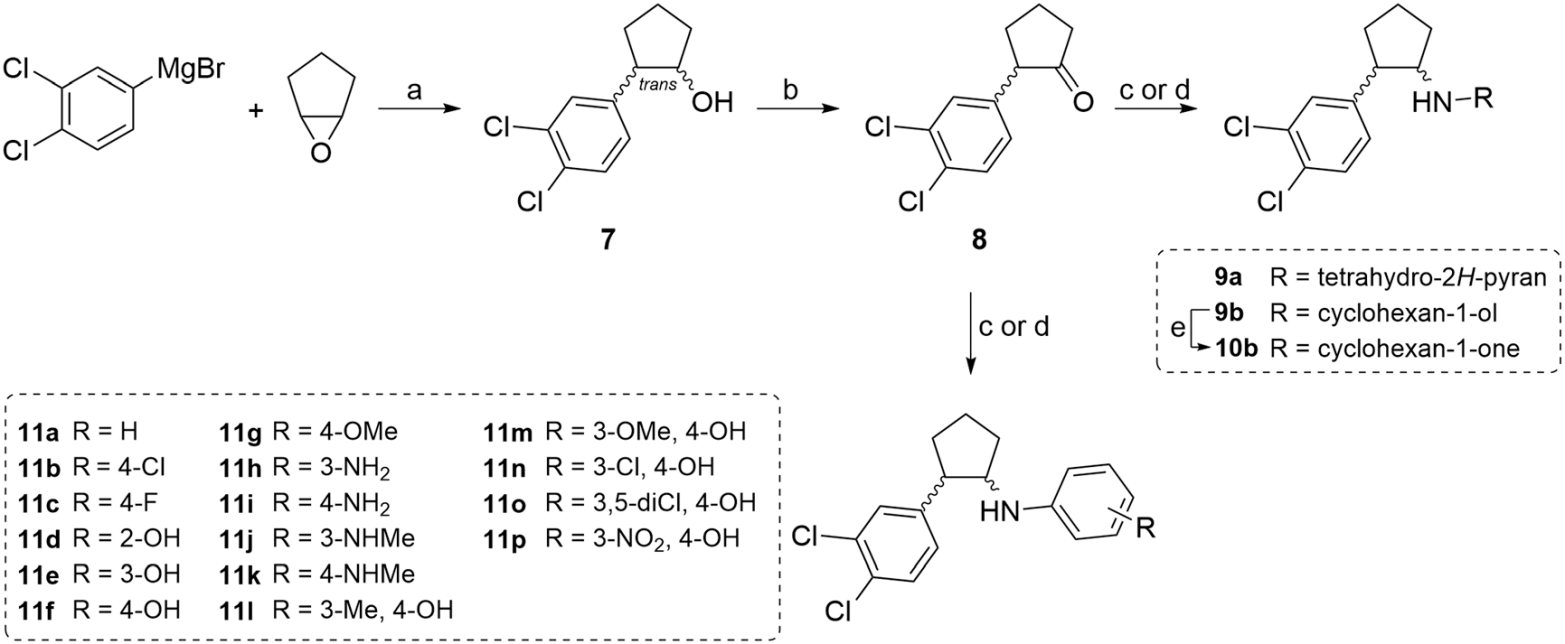
Synthesis of cycloalkylamines 9a, 10b and 11a–11p. Reagents and conditions: a) cat. Cu^I^I, THF, rt, 5 h, 83%; b) Dess–Martin periodinane, DCM, 0 °C to rt, 17 h, 96%; c) corresponding amine, AcOH, NaBH(OAc)_3_, DCM, rt, 1–5 days, 3–68%; d) corresponding amine, NaBH_3_CN, AcOH, MeOH or EtOH, 60 °C, 2–5 days, 12–25%; e) Dess–Martin periodinane, DCM, 0 °C to rt, 19 h, 20%.

Compounds were synthesized as previously described for the stereospecific synthesis of *N*,2-substituted cycloalkylamines (Scheme 1).^25^ In short, after Grignard epoxide opening (intermediate 7) and subsequent oxidation of the formed alcohol (ketone intermediate 8), a series of reductive aminations were carried out with the use of two aliphatic amines and various substituted anilines. Final aliphatic compound 9a and intermediate 9b were obtained with the building blocks tetrahydro-2*H*-pyran-4-amine and 4-aminocyclohexanol, respectively. Following a Dess–Martin oxidation, intermediate 9b, the isomeric mixture of our previously reported 6, was converted into cyclopentanone 10b. Final compounds 11a–11p were synthesized under classic reductive amination conditions with NaBH(OAc)_3_ or NaBH_3_CN using their corresponding substituted anilines. Of note, all aniline-containing final compounds (11a–11o) except for NO_2_-substituted 11p, were identified as exclusively containing *cis*-oriented cyclopentylamines. This *cis*-selectivity has been observed previously in a simplified system^26^ and was confirmed here based on previously described proton-coupling patterns of specific cycloalkylamine stereoisomers (exemplary 2D NMR spectra in SI).^25^ Our previous research, detailing the stereospecific synthesis, demonstrated that the inhibitory potency differences between the two *trans*-oriented cyclopentyl enantiomers, as well as between the two *cis*-oriented enantiomers, are minimal. Therefore, the inhibitors presented here, assumed to be racemic mixtures of both *cis*-oriented enantiomers, were kept as racemic mixtures. Compound 11p, however, was found to contain both *trans*- and *cis*-oriented species in a 1 : 3 ratio (SI).

### 2.2 *In vitro* hMAT characterization

**Fig. 2:**
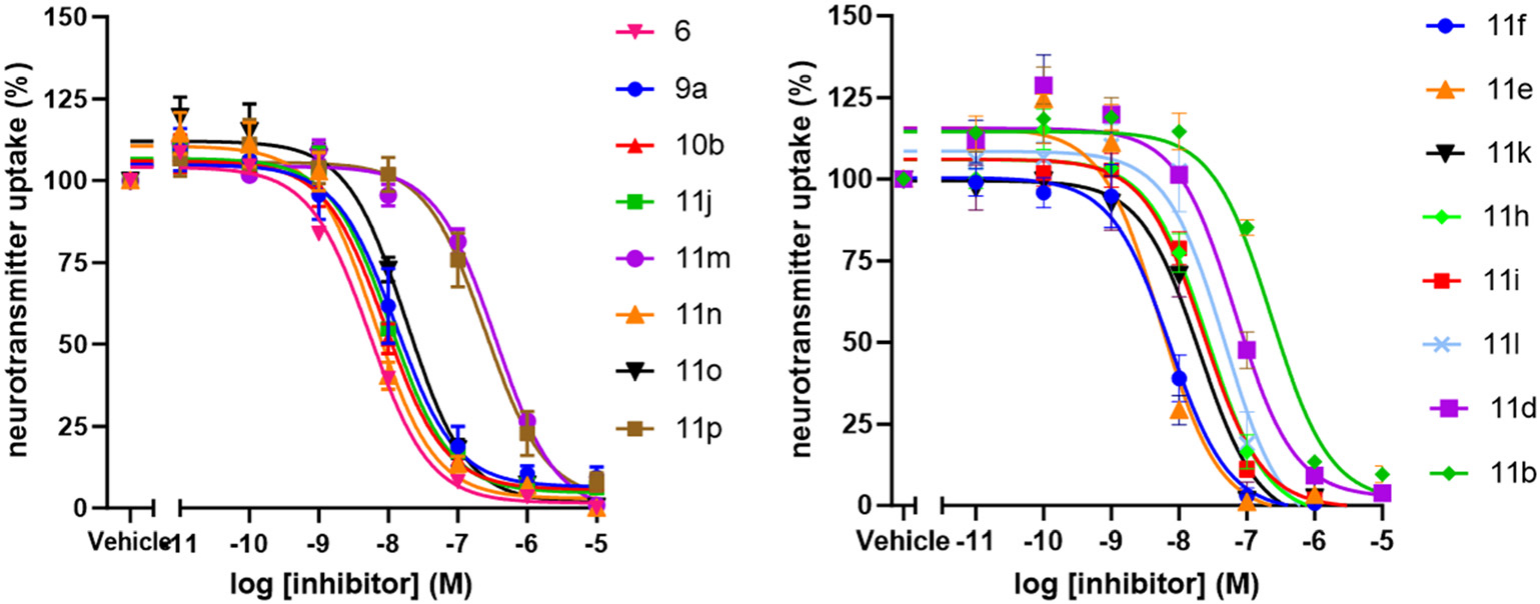
Dose-dependent inhibition of DAT-mediated fluorescent neurotransmitter uptake by cycloalkylamine derivatives. HEK293-JumpIn-DAT cells were treated with 100 μM fluorescent neurotransmitter dye and increasing concentration of cycloalkylamine inhibitors and uptake of fluorescent neurotransmitter was analyzed by AUC over 60 min after stimulation.

With the compounds in hand, the MAT-inhibitory properties were tested with the use of fluorescent uptake assays. The novel series of *N*,2-substituted cyclopentylamines, as well as parent compound 6, were screened for their ability to inhibit the NET, DAT and SERT mediated reuptake of 100 μM of a fluorescent neurotransmitter by adding them at a 1 μM concentration to a HEK293-JumpIn cell line with doxycycline-inducible expression of hNET, hDAT or hSERT (HEK293-JumpIn-NET, HEK293-JumpIn-DAT, HEK293-JumpIn-SERT, respectively). All compounds that showed more than 50% inhibition were tested in a full concentration range to determine their respective pIC_50_ value for each MAT (Fig. 2, Table 1, Fig. S1 and S2).

**Table 1:** Inhibitory potency values or percentage inhibition at 1 μM of reference inhibitors nisoxetine and GBR-12909 and cycloalkylamine derivatives 6, 9a, 10b and 11a–11p in HEK293-JumpIn cells expressing the corresponding MAT, determined with a fluorescent uptake assay

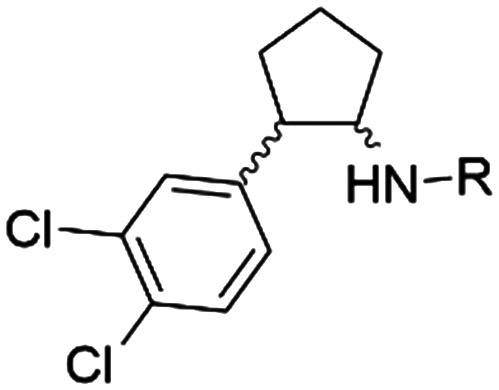				
Cmpd	R	NET	DAT	SERT
		pIC_50_ ± SEM (IC_50_^a^ (nM)) or inhibition at 1^b^ μM	pIC_50_ ± SEM (IC_50_^a^ (nM))	Inhibition at 1^b^ μM
Nisoxetine		8.2 ± 0.0 (6.3)	>6^c^	N.D.
GBR-12909		6.1 ± 0.2 (794)	8.6 ± 0.1 (2.5)	N.D.
6	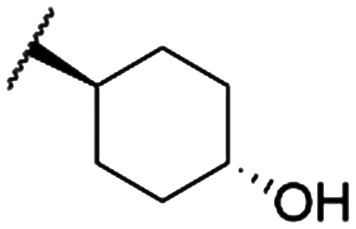	8.4 ± 0.1 (4.0)	9.4 ± 0.1 (0.4)	26% (18, 33)
9a	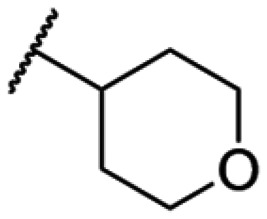	7.1 ± 0.1 (79)	7.9 ± 0.2 (13)	7% (1, 12)
10b	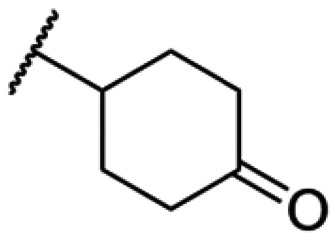	6.9 ± 0.1 (126)	8.1 ± 0.1 (7.9)	5% (−1, 11)
11a	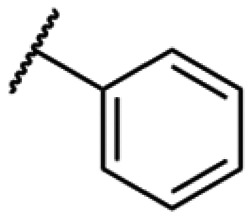	1% (4, −6)	7.2 ± 0.1 (63)	−2% (1, −6)
11b	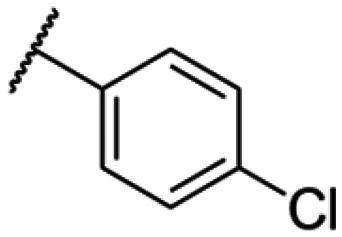	−4% (1, −8)	6.6 ± 0.0 (251)	−1% (−2, 1)
11c	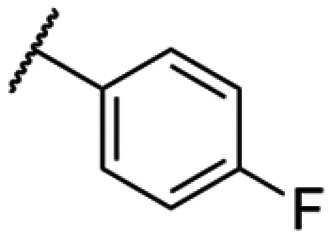	−8% (0, −16)	6.9 ± 0.1 (126)	−4% (−11, 3)
11d	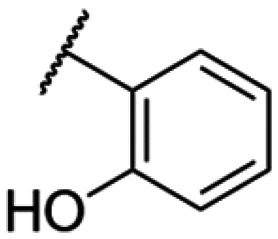	35% (31, 38)	7.2 ± 0.1 (63)	−21% (−24, −18)
11e	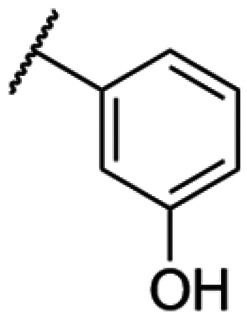	29% (27, 30)	8.3 ± 0.1 (5.0)	2% (−2, 7)
11f	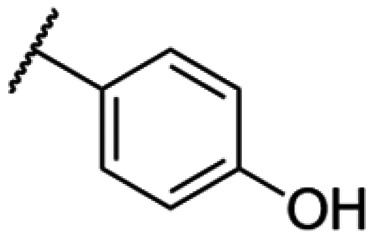	26% (25, 27)	8.2 ± 0.1 (6.3)	13% (9, 17)
11g	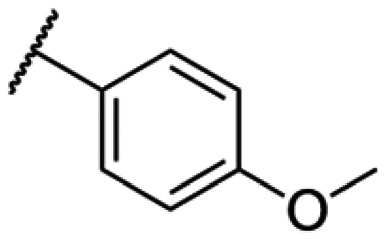	2% (7, −4)	7.0 ± 0.1 (100)	7% (1, 14)
11h	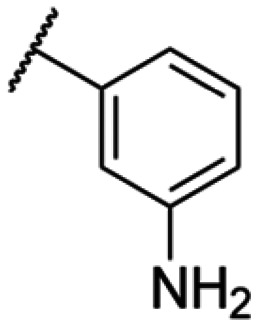	−1% (1, −3)	7.6 ± 0.1 (25)	10% (2, 17)
11i	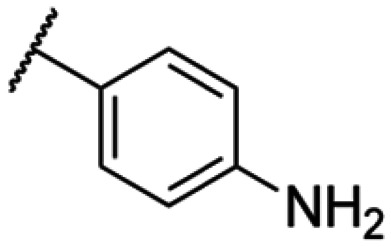	7.0 ± 0.0 (100)	7.7 ± 0.1 (20)	11% (4, 17)
11j	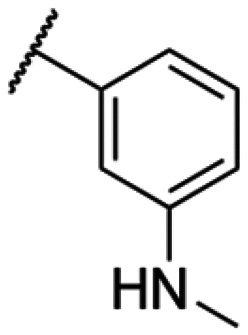	3% (4, 2)	8.0 ± 0.0 (10)	3% (−1, 7)
11k	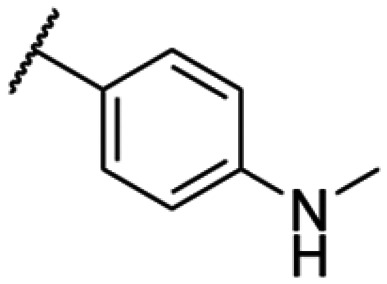	6.6 ± 0.0 (251)	7.7 ± 0.1 (20)	8% (5, 11)
11l	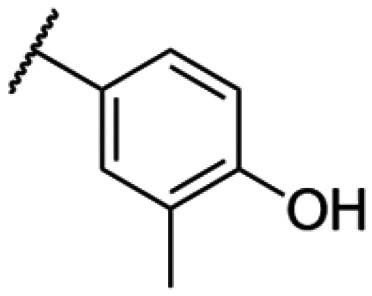	−5% (−2, −7)	7.3 ± 0.1 (50)	1% (−1, 2)
11m	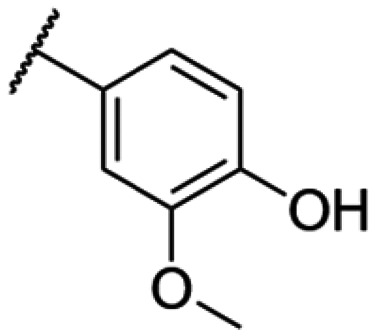	−4% (−2, −6)	6.5 ± 0.0 (316)	7% (6, 8)
11n	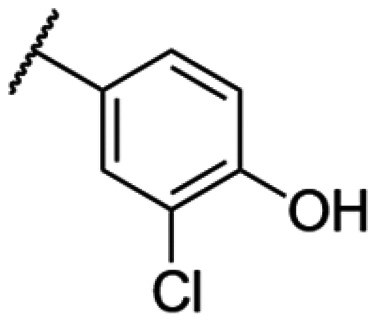	36% (37, 36)	8.2 ± 0.1 (6.3)	3% (0, 5)
11o	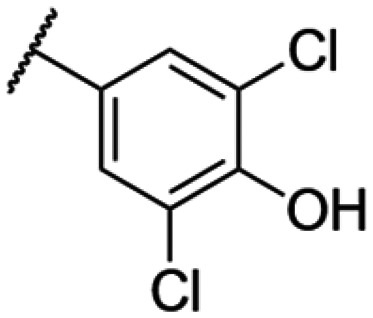	46% (48, 43)	7.7 ± 0.0 (20)	1% (4, −1)
11p	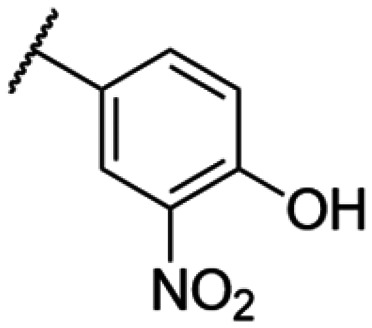	−17% (−19, −15)	6.6 ± 0.1 (251)	−197% (−215, −178)^d^

a pIC_50_ values are presented as mean ± SEM of three independent experiments performed in duplicate.

b Percentage inhibition values represent mean with individual values between brackets of two independent experiments performed in duplicate.

c Based on IC_50_ value obtained by Ahn *et al.*^27^

d Enhancement of the fluorescent signal was observed, which was not further explored in an orthogonal assay and is interpreted as a limitation of the fluorescent uptake assay. N.D.: not determined.

In our previous work, compound 6 was fully characterized for NET displaying a pIC_50_ value of 8.4 ± 0.1 in a label-free transporter activity through receptor activation (TRACT) functional assay.^25^ In the fluorescent uptake assay, 6 showed similar NET inhibitory properties with a pIC_50_ value of 8.4 ± 0.1. Strikingly, a 10-fold higher inhibitory potency for DAT (pIC_50_ of 9.4 ± 0.1) and a low activity towards SERT (26% inhibition at 1 μM) was found, showcasing the dual reuptake inhibiting properties of 6 as a NDRI. A similar trend was observed for the other two aliphatic derivatives 9a bearing a tetrahydro-2*H*-pyran (NET pIC_50_ of 7.1 ± 0.1, DAT pIC_50_ of 7.9 ± 0.2) and 10b bearing a cyclohexanone (NET pIC_50_ of 6.9 ± 0.1, DAT pIC_50_ of 8.1 ± 0.1), albeit with a log unit lower potency for both NET and DAT compared to 6 and a negligible activity for SERT. Although changes in basicity, geometry, or hydrophobic contacts may also contribute, the 10-fold decrease in inhibitory activity for 9a and 10b may be explained by the loss of a hydrogen bond between the hydroxyl group of 6 with NET residue D473, analogous to D476 in DAT.^25^ This residue is involved in the salt-bridge gating mechanism with a respective arginine (R81 for NET and R85 for DAT) and is essential to close the extracellular cavity during transport previously hypothesized in mutagenesis studies^28,29^ and validated by the recently published NET and DAT cryo-EM structures.^20,30–32^

The higher activity for DAT over NET, combined with the structure–activity relationships (SARs) reported for DRIs such as benztropines and modafinil analogues as compounds with multiple phenyl moieties,^33,34^ prompted us to replace the aliphatic amine with several substituted anilines (11a–11p). This allowed investigation of the impact of two substituted phenyl rings on both sides of the core of the molecule. Upon characterization of the inhibitory properties, most of the aromatically substituted compounds (11a–11h, 11j, 11m–11n, and 11p) were found to completely lose NET-activity. Unsubstituted aniline 11a showed moderate DAT potency (7.2 ± 0.1), which slightly decreased upon substitution with an electron withdrawing chlorine or fluorine atom at the *para*-position (11b and 11c, respectively). In order to mimic parent compound 6, several hydroxyl-substituted derivatives were synthesized 11d–11f, albeit with different electrostatic properties compared to aliphatic alcohols. The 2-OH derivative 11d showed equal inhibitory activity for DAT (pIC_50_ of 7.2 ± 0.1) and a slight NET activity of 35% at 1 μM compared to the unsubstituted 11a. On the other hand, 11e and 11f with 3-OH and 4-OH substitution patterns, respectively, showed nanomolar inhibitory potency for DAT (pIC_50_ of 8.3 ± 0.1 and 8.2 ± 0.1) with slight NET activity (29% and 26% at 1 μM) and no SERT activity at 1 μM. Changing the electrostatic properties as well as the hydrogen bond forming capacity by converting 4-OH in 4-OMe in 11g decreased DAT potency around 20-fold but maintained selectivity.

The hydroxyl groups were substituted for amines and methylamines in compounds 11h–11k, for which remarkably both *meta*-substituted versions (11h and 11j) retained selectivity for DAT over the other MATs with high inhibitory potencies of 7.6 ± 0.1 and 8.0 ± 0.0, respectively. Contrarily, *para*-substituted amine 11i and methylamine 11k displayed equal potency for DAT (pIC_50_ of 7.7 ± 0.1) and also showed moderate potency for NET. The results from substituting with amines and methylamines suggest that these compounds are still able to maintain interactions with the aforementioned DAT residue D476 but potentially to a lesser extent compared to hydroxyl-substituted 11e and 11f.

To assess the relationship between the p*K*_a_ of the 4-OH and activity, the addition of electron donating groups such as a methyl (11l) or methoxy (11m) was pursued. These substitutions decreased DAT inhibitory properties to 7.3 ± 0.1 and 6.5 ± 0.0, respectively, compared to 11f while maintaining selectivity. Alternatively, substituting one *meta*-position with a chlorine (11n) led to similar DAT inhibition compared to 11f (pIC_50_ of 8.2 ± 0.1) again with low inhibition of NET transport (36% at 1 μM). However, the substitution of both *meta*-positions with chlorines (11o) decreased DAT activity (pIC_50_ of 7.7 ± 0.0) and slightly increased NET activity at 1 μM to 46%. Compound 11p, where the electron withdrawing properties at the *meta*-position were further increased to modulate the 4-OH substituent by the addition of a nitro-group, inhibited DAT transport with decreased activity (pIC_50_ of 6.6 ± 0.1). Notably, although no inhibitory activity against NET was found for 11p, the SERT assay did reveal an apparent increased uptake of fluorescent neurotransmitter (−197%) in the presence 1 μM of 11p, compared to vehicle (Fig. S2). Because this finding was not further explored in an orthogonal assay, these result should be interpreted cautiously. The observed increase in fluorescence may reflect assay interference (potentially caused by the UV absorption properties of the *o*-nitrophenol moiety^35^) or an indirect off-target mechanism (*e.g.* GLUT1 inhibition could alter local glucose levels, which can artificially enhance SERT-mediated uptake^36^), rather than definitive evidence for enhanced SERT-mediated transport. Ultimately, this ambiguity represents a limitation in the experimental setup. The overall trend that *N*-phenyl substituted derivatives show a decreased DAT inhibitory activity compared to the aliphatic parent compound 6 can be a consequence of changing the secondary amine into an aniline and therefore its p*K*_a_. Hence, compounds 11a–11p will be unprotonated at physiological pH and have diminished electrostatic interactions with residue D79, the conserved aspartic acid amongst all MATs responsible for interacting with the terminal amines of the endogenous substrates during transport.^37^ Nevertheless, protonated amines are not essential for DAT inhibition as was showcased by substituting the amine for ether functionalities in phenyltropane and methylphenidate derivatives.^38,39^

### 2.3 Computational characterization of selectivity

**Fig. 3:**
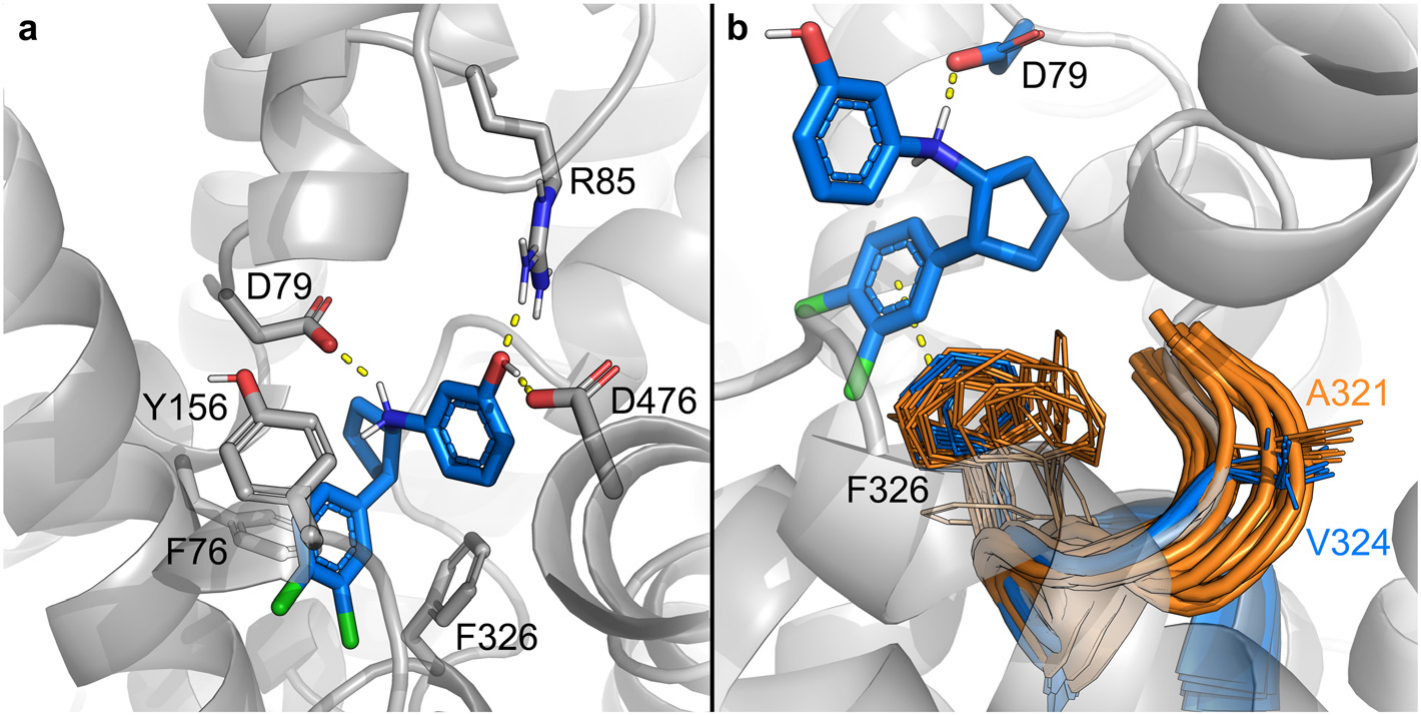
(a) Putative binding orientation of compound 11e ((1*R*,2*R*)-isomer shown in blue) in hDAT (PDB: 9EO4), hydrogen bond (to R85 and D476) and salt bridge (to D79) interactions are represented by a dashed line. (b) The edge-to-face π–π stacking interaction with F326 (DAT in blue; F323 for NET in orange) is hypothesized to be a key contributor to the selectivity of 11e towards DAT. This residue is found on a flexible loop region between TM6a and TM6b, represented as cartoon for NET (orange) and DAT (blue) obtained from all available NET and DAT structures. The loop contains a valine in DAT (V324), which restricts the movement of this region, as compared to alanine in NET (A321).

To provide insights into the key determinants of binding and selectivity of our new compounds towards DAT, we performed a docking study of one of the most potent and selective compounds, 11e (Fig. 3a), using the recently published cryo-EM structure bound with cocaine (PDB: 9EO4). A canonical salt bridge interaction is predicted between D79 and the secondary amine.^40^ In turn, the *m*-OH substituent is predicted to be oriented towards a salt bridge formed by R85 and D476, serving as an extracellular gating mechanism during transport, as was also observed for compound 6 in the NET binding pocket with its corresponding residue D473 but not R81.^25,41^ Upon accommodation of the phenol moiety, the 3,4-dichlorophenyl moiety is predicted to be accommodated deeper in the binding site in a pocket lined by residues F76, Y156, F326. The latter is part of the flexible loop region between TM6a and TM6b and forms an edge-to-face π–π stacking interaction with the 3,4-dichlorophenyl moiety as similarly observed in the hDAT cryo-EM structure with the phenyl moiety of methylphenidate.^20,42^ A structural analysis of all available NET and DAT structures shows that this region is more flexible in NET than in DAT (Fig. 3b). Further analysis shows that this flexibility might originate from a single amino acid change, where a valine (V324) in DAT is an alanine (A321) in NET. The former is a bulkier residue, which restricts the movement of this loop and stabilizes the orientation of F326, such that the edge-to-face π–π interaction with F326 becomes more stable in the DAT which may contribute to the selectivity observed for the aromatically-substituted compounds and might provide a valuable avenue for future selective ligand design.

To provide further insights in the determinants of selective recognition of compound 11e, we performed free energy calculations of binding for selected compounds (6 to 11e and 11e to 11f) on hNET. In addition, we calculated the free energy differences of the A321V mutation for compound 11e. As shown in Table 2, compound 6 was predicted to be 1.84 kcal mol^−1^ more potent than 11f, which is in agreement, though underpredicted, with the >3.27 kcal mol^−1^ our experimentally determined IC_50_-values would suggest. In contrast, no markable difference in free energies was observed between 11e and 11f, which is in agreement with the experimental values showing no difference between these two compounds. Finally, we showed that, when calculating the binding energy of 11e to WT hNET *versus* the A231V mutant thereof (essentially mimicking the hDAT structure), the predicted binding energy increased by 1.53 kcal mol^−1^, which is in agreement with the observed increase in affinity of 11e towards hDAT. Taken together, these calculations show clear correlations with the experimental values, underpinning the selectivity observed experimentally.

**Table 2:** Calculated and experimental changes in free energy for two compounds and the A324V mutation of hNET

Perturbation	ΔΔ*G*_calc_ (kcal mol^−1^)	ΔΔ*G*_exp_^a^ (kcal mol^−1^)
**QligFEP**		
6 → 11f	1.84 ± 0.71	3.27
11e → 11f	0.30 ± 0.13	0.00
**QresFEP**		
A231V (11e)	−1.53 ± 0.71	−3.14

a Experimental values were obtained by converting the relative IC_50_ values obtained in the corresponding experiments (binding of 6, 11e and 11f to hNET, as well as binding of 11e to hDAT) to Gibbs free energy using the free energy equation.

### 2.4 *In vivo* characterization in *Drosophila melanogaster*

The dopaminergic system is evolutionarily conserved, and *Drosophila melanogaster* expresses both D_1_-like and D_2_-like dopamine receptors, along with a single dopamine transporter (dDAT).^43^ Dopaminergic neurons in flies express dDAT, which exhibits substrate specificity similar to that of its human counterpart, hDAT.^44–46^ Knockout of dDAT, also known as *fumin*, results in a hyperlocomotive and sleepless phenotype in fruit flies, which can be rescued by expressing functional hDAT. Therefore, *Drosophila* serves as a powerful model to study DAT inhibitors. We investigated the *in vivo* inhibitory activity of parent compound 6 and two newly synthesized *N*,2-substituted cycloalkylamines, 11e and 11f, in this model.

To assess the flies' consumption of *N*,2-substituted cycloalkylamine-based inhibitors, humanized flies expressing hDAT in a *fumin* background were starved for 20 to 22 h and transferred to the FlyPad arena. This contained glucose food pellets supplemented either with DMSO (control), control inhibitors (nisoxetine and GBR-12909), or the novel inhibitors, all at concentrations of 100 times their IC_50_ values (Table 1). Importantly, there was no significant difference in food consumption across conditions (Fig. S3). Locomotor activity of *Drosophila* expressing hDAT was then measured using the DAM5H *Drosophila* Activity Monitor.

**Fig. 4:**
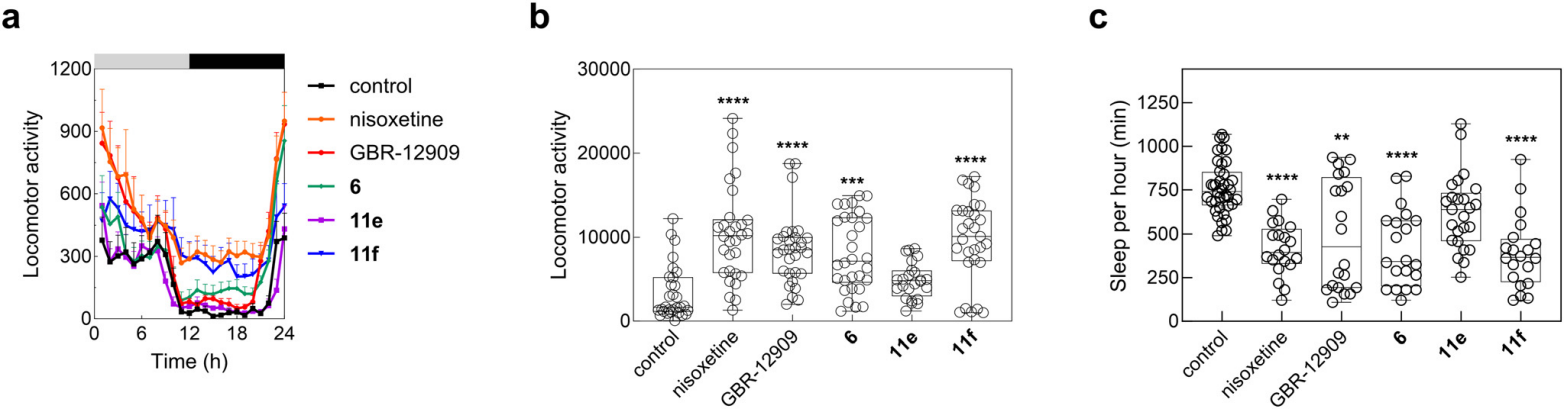
(a) Locomotion of individually housed three- to five-day-old male flies was studied using a DAM5H *Drosophila* Activity Monitor (TriKinetics, USA). The *w; fmn (w; roo{}DAT*^*fmn*^*); UAS-YFP-hDAT* flies received food supplemented with DMSO (control) or inhibitors for 48 h. The flies were subsequently anaesthetized using CO_2_ and housed in individual 5 mm diameter polycarbonate tubes, containing food pellet supplemented with DMSO (control) or inhibitors. To isolate hDAT-mediated effects from light-mediated effects, flies were entrained for the first two days with 12 h light/12 h dark cycles, followed by 12 h dark/12 h dark cycles (indicated by the grey and black bars).^46^ Locomotion data was recorded at 1 min intervals and grouped into 60 min bins. Displayed above are data from the second day of the 12 h dark/12 h dark cycle. (b) Fly locomotion activity in 12 h dark/12 h dark cycles was measured for a period of 4 days. The conditions were statistically analyzed by the Kruskal–Wallis test, followed by Dunn's multiple comparison (*p = 0.0342, ***p = 0.0003, ****p < 0.0001, significantly different from control). (c) The total duration of sleep was calculated for flies tested in each of the experimental conditions. Kruskal–Wallis test, followed by Dunn's multiple comparison, indicated statistically distinct effects (**p = 0.0014, ****p < 0.0001, significantly different from control). Data represent mean ± SEM from three independent experiments, which were carried out in parallel with at least 8 flies per condition.

Flies that received the control inhibitors, nisoxetine or GBR-12909, displayed an increase in locomotor activity compared to the control flies (Fig. 4a and b). Furthermore, upon quantifying the total sleep time, these inhibitors significantly reduced the time of sleep, relative to the control condition. Even though nisoxetine is reported as a selective norepinephrine reuptake inhibitor (NRI) with a reported NET IC_50_ value in the low nanomolar range, the inhibitor also displays some inhibitory activity in the low micromolar range explaining the observed effect on the hDAT expressing flies.^27^ Similar to the control inhibitors, the previously reported aliphatic *N*,2-substituted cycloalkylamine, 6, now identified as a NDRI, increased the locomotor activity and significantly reduced the total sleep time in flies. Among the selective DRIs, 11e and 11f, which both feature a hydroxyl substitution on the aromatic ring at the *meta*- and *para*-positions, respectively, the effect on locomotor activity was notable. 11f increased locomotor activity, whereas this effect was absent for 11e, once again consistent with the effect observed from the calculated sleep time, which was significantly reduced by 11f, but not by 11e. The differences between 11e and 11f observed in the *Drosophila* model were not further explored but trivial reasons for this difference might be solubility, blood–brain barrier penetration or the presence of metabolic enzymes that favor a *meta*- over *para*-hydroxyl substituted inhibitor.

## 3. Conclusions

In this study, we further investigated the previously reported *N*,2-substituted cycloalkylamine scaffold to develop selective DAT inhibitors. Eighteen new derivatives were designed, synthesized, and evaluated for their inhibitory properties, using *in vitro* fluorescent uptake assays and *in vivo* locomotor activity experiments in *Drosophila melanogaster*. Characterization of the compounds across all monoamine transporters revealed minimal SERT activity, while some aliphatic derivatives (6, 9a, and 10b) demonstrated a dual norepinephrine–dopamine reuptake inhibition profile. The aromatically substituted derivatives were primarily selective for DAT, except for compounds 11i and 11k, and exhibited inhibitory potencies ranging from submicromolar to nanomolar concentrations. Computational docking studies predicted key interactions with residues R85 and D476, which are associated with strong inhibitory effects. Additionally, stabilization of residue F326 within the flexible loop region between transmembrane domains TM6a and TM6b was suggested to contribute to DAT selectivity. In hDAT-expressing *Drosophila melanogaster*, 3-OH-substituted compound 11e did not significantly affect locomotor activity or sleep time compared to the control. In contrast, compound 11f, featuring a 4-OH substitution, did affect both locomotor activity and sleep time significantly comparable to the observed effects of reference inhibitors nisoxetine and GBR-12909. While the aromatic substitution did lead to improved selectivity for 11f, it also introduced potential ADME/tox liabilities through the *p*-hydroxyaniline moiety, warranting caution in future research. Together, these findings offer valuable insights into DAT-targeting therapeutic strategies and highlight the potential and limitations of this chemotype for further exploration in the context of DAT inhibition.

## 4. Experimental section

### 4.1 Chemistry

#### 4.1.1 General chemistry

All solvents and reagents were purchased from commercial sources and were of analytical grade. Demineralized water is referred to as H_2_O and was used in all cases unless noted otherwise (*i.e.*, brine). All reactions were routinely monitored with thin-layer chromatography (TLC), using aluminum silica gel coated 60 F_254_ plates from Merck (Darmstadt, Germany) and visualized by UV irradiation at 254 nm and subsequent staining with ceric ammonium molybdate, KMnO_4_ or ninhydrin solution. Purification by flash column chromatography was carried out with the use of silica gel irregular ZEOprep® particles (60–200 μm) from VWR (Amsterdam, The Netherlands) or by using an Isolera™ One or Selekt automatic flash chromatography system from Biotage® (Uppsala, Sweden) with pre-packed cartridges (Phenomenex (Torrance, CA, USA) Gemini® Claricep™ (silica) or Biotage® Sfär C18 D Duo 100 Å 30 μm (C18)). Solutions were concentrated using a Heidolph (Schwabach, Germany) Hei-VAP Value rotary evaporator. Nuclear magnetic resonance (NMR) spectra were recorded on a Bruker (Billerica, MA, USA) AV-400 liquid or AV-400 WB spectrometer (^1^H NMR, 400 MHz and ^13^C NMR, 101 MHz) at ambient temperature and subsequently analyzed with MestReNova v14.1.0 software (Mestrelab Research S.L., Santiago de Compostela, Spain). Chemical shifts are reported in parts per million (ppm), designated by *δ* and corrected to the internal standard tetramethylsilane (*δ* = 0). Multiplicities are indicated by s, singlet; d, doublet; dd, doublet of doublets; ddd, doublet of doublet of doublets; dddd, doublet of doublet of doublet of doublets; dtd, doublet of triplet of doublets; t, triplet; dt, doublet of triplets; tt, triplet of triplets; q, quartet; p, pentet; m, multiplet; br s, broad singlet. Coupling-constants (*J*) are reported in Hz. Mass and compound purity analyses were performed with liquid chromatography-mass spectrometry (LC-MS) using an LCMS-2020 system from Shimadzu (Kyoto, Japan) coupled to a Phenomenex Gemini® C18 110 Å column (50 mm × 3 mm × 3 μm). Samples were prepared by dissolving 0.3–0.8 mg of compound in 1 mL of a 1 : 1 : 1 mixture of CH_3_CN/H_2_O/*t*BuOH and were eluted using an isocratic system of H_2_O/CH_3_CN with 0.1% FA, using gradients from 100 : 0 to 60 : 40 and 90 : 10 to 10 : 90 in an elution time of 15 minutes. All tested compounds were determined to be of >95% purity as determined by HPLC measuring UV absorption at 215 nm or 254 nm.

#### 4.1.2 Synthetic procedures

##### 2-(3,4-Dichlorophenyl)cyclopentan-1-ol (7)

Intermediate 7 was synthesized following our previously described procedure.^25^ Automatic column chromatography (silica) with 15 to 80% DCM in PE as mobile phase on Biotage® Isolera™ One gave 7 as a yellow oil (3.82 g, 16.5 mmol, 83%). ^1^H NMR (400 MHz, CDCl_3_) *δ* 7.32 (d, *J* = 8.3 Hz, 1H), 7.30 (d, *J* = 2.1 Hz, 1H), 7.03 (dd, *J* = 8.3, 2.1 Hz, 1H), 3.94 (q, *J* = 7.1 Hz, 1H), 3.05 (s, 1H), 2.72 (dt, *J* = 10.2, 7.9 Hz, 1H), 2.13–1.92 (m, 2H), 1.85–1.67 (m, 2H), 1.66–1.50 (m, 2H). HPLC *t*_R_: 10.697 min.

##### 2-(3,4-Dichlorophenyl)cyclopentan-1-one (8)

Intermediate 7 (2.00 g, 8.65 mmol, 1.0 equiv.) was dissolved in DCM (43 mL) and allowed to cool down to 0 °C after which Dess–Martin periodinane (3.67 g, 8.65 mmol, 1.0 equiv.) was added. The mixture was warmed up to rt and stirred for 17 h followed by dilution with DCM (60 mL). After quenching with 1 N NaOH (50 mL), the organic phase was separated, dried over MgSO_4_, filtrated and concentrated *in vacuo*. Automatic column chromatography (silica) with 15 to 80% DCM in PE as mobile phase on Biotage® Isolera™ One provided intermediate 8 as a yellow oil (1.90 g, 6.29 mmol, 96%). ^1^H NMR (400 MHz, CDCl_3_) *δ* 7.36 (d, *J* = 8.3 Hz, 1H), 7.28 (d, *J* = 2.2 Hz, 1H), 7.03 (dd, *J* = 8.3, 2.2 Hz, 1H), 3.25 (dd, *J* = 12.0, 8.8 Hz, 1H), 2.60–2.36 (m, 2H), 2.33–1.72 (m, 4H). HPLC *t*_R_: 10.935 min.

##### General procedure A

Intermediate 8 and appropriate amine or aniline were dissolved in DCM after which acetic acid and NaBH(OAc)_3_ were added sequentially. To account for varying substrate p*K*_a_ and resulting reaction variability, the reaction mixture was stirred at rt for 1 to 5 days while routinely monitoring the pH (using pH paper, a pH between 5–6 was maintained by adding additional acetic acid when required) and reaction progress (using TLC). Upon completion, the reaction mixture was quenched with NaHCO_3_ followed by addition of EtOAc. The aqueous phase was extracted three times with EtOAc, after which the combined organic phases were dried over MgSO_4_, filtrated and concentrated *in vacuo*. Flash column chromatography or automatic column chromatography provided final compounds 9a, 11a–11m and 11q.

##### General procedure B

Intermediate 8 and appropriate amine or aniline were dissolved in MeOH or EtOH after which acetic acid and NaBH_3_CN were added sequentially. To account for varying substrate p*K*_a_ and resulting reaction variability, the reaction mixture was stirred at 60 °C for 2 to 5 days while routinely monitoring the pH (using pH paper, a pH between 5–6 was maintained by adding additional acetic acid when required) and reaction progress (using TLC). Upon completion, the reaction mixture was quenched with NaHCO_3_ followed by addition of EtOAc. The aqueous phase was extracted three times with EtOAc, after which the combined organic phases were dried over MgSO_4_, filtrated and concentrated *in vacuo*. Flash column chromatography or automatic column chromatography provided intermediate 9b and final compounds 11n–11p.

##### *N*-(2-(3,4-Dichlorophenyl)cyclopentyl)tetrahydro-2*H*-pyran-4-amine (9a)

Final compound 9a was obtained following general procedure A with a 1 : 2 : 1 : 2 stoichiometry of intermediate 8, tetrahydro-2*H*-pyran-4-amine, acetic acid and NaBH(OAc)_3_, respectively. Flash column chromatography with 5% MeOH + 1% Et_3_N in DCM as mobile phase gave 9a as a yellow oil (138 mg, 0.44 mmol, 41%). ^1^H NMR (400 MHz, CDCl_3_) *δ* 7.37 (d, *J* = 8.2 Hz, 1H), 7.34 (d, *J* = 2.1 Hz, 1H), 7.08 (dd, *J* = 8.3, 2.1 Hz, 1H), 3.93–3.79 (m, 2H), 3.42 (q, *J* = 5.6 Hz, 1H), 3.29 (dtd, *J* = 21.4, 11.5, 2.4 Hz, 2H), 3.13 (q, *J* = 7.2, 1H), 2.45 (tt, *J* = 10.5, 4.0 Hz, 1H), 2.06–1.85 (m, 4H), 1.77–1.65 (m, 2H), 1.65–1.52 (m, 2H), 1.24 (dddd, *J* = 13.0, 11.6, 10.4, 4.4 Hz, 1H), 1.12 (dddd, *J* = 13.0, 11.5, 10.5, 4.5 Hz, 1H), 0.77 (br s, 1H). ^13^C NMR (101 MHz, CDCl_3_) *δ* 142.4, 132.2, 130.8, 130.2, 130.1, 128.3, 67.1, 67.0, 58.8, 52.0, 48.7, 34.6, 33.7, 32.6, 29.3, 22.3. LC-MS (ESI^+^) *m*/*z* calcd. for C_16_H_21_Cl_2_NO [(M + H)]^+^: 314.11; found 314.00. HPLC *t*_R_: 6.702 min.

##### 4-((2-(3,4-Dichlorophenyl)cyclopentyl)amino)cyclohexan-1-ol (9b)

Intermediate 9b was obtained following general procedure B in MeOH with a 1 : 3 : 3 : 4 stoichiometry of intermediate 8, *trans*-4-aminocyclohexanol, acetic acid and NaBH(OAc)_3_, respectively. Flash column chromatography with 7% MeOH + 0.5% Et_3_N in DCM as mobile phase gave 9b as a yellow oil (463 mg, 1.41 mmol, 18%). ^1^H NMR (400 MHz, CDCl_3_) *δ* 7.36 (d, *J* = 8.3 Hz, 1H), 7.32 (d, *J* = 2.1 Hz, 1H), 7.06 (dd, *J* = 8.3, 2.1 Hz, 1H), 3.56–3.43 (m, 1H), 3.37 (q, *J* = 6.0 Hz, 1H), 3.15 (q, *J* = 7.4 Hz, 1H), 2.23 (tt, *J* = 10.9, 3.8 Hz, 1H), 2.08–1.79 (m, 7H), 1.74–1.49 (m, 3H), 1.29–1.10 (m, 2H), 1.07–0.93 (m, 1H), 0.93–0.79 (m, 1H). ^13^C NMR (101 MHz, CDCl_3_) *δ* 142.4, 132.2, 130.7, 130.1, 130.0, 128.2, 70.4, 59.7, 54.2, 48.3, 34.2, 32.4, 31.8, 31.2, 29.4, 22.1. LC-MS (ESI^+^) *m*/*z* calcd. for C_17_H_23_Cl_2_NO [(M + H)]^+^: 328.12; found 328.00. HPLC *t*_R_: 6.390 min.

##### 4-((2-(3,4-Dichlorophenyl)cyclopentyl)amino)cyclohexan-1-one (10b)

Intermediate 9b (100 mg, 0.31 mmol, 1.0 equiv.) was dissolved in DCM (2.5 mL) and allowed to cool down to 0 °C after which Dess–Martin periodinane (155 mg, 0.37 mmol, 1.2 equiv.) was added. The mixture was warmed up to rt and stirred for 19 h followed by dilution with DCM (15 mL) and quenching with 1 N NaOH (20 mL). The aqueous phase was extracted with DCM (20 mL), after which the combined organic phases were dried over MgSO_4_, filtrated and concentrated *in vacuo*. Automatic column chromatography (silica) with 20 to 50% EtOAc in PE as mobile phase on Biotage® Isolera™ One provided final compound 10b as a yellow oil (20.0 mg, 0.06 mmol, 20%). ^1^H NMR (400 MHz, CDCl_3_) *δ* 7.37 (d, *J* = 8.3 Hz, 1H), 7.34 (d, *J* = 2.4 Hz 1H), 7.09 (dd, *J* = 8.5, 2.1 Hz, 1H), 3.39 (q, *J* = 5.9 Hz, 1H), 3.19 (q, *J* = 7.6 Hz, 1H), 2.75 (tt, *J* = 8.5, 3.5 Hz, 1H), 2.45–2.36 (m, 1H), 2.35–2.12 (m, 3H), 2.11–1.89 (m, 5H), 1.89–1.79 (m, 1H), 1.79–1.67 (m, 1H), 1.67–1.50 (m, 2H), 1.49–1.36 (m, 1H). ^13^C NMR (101 MHz, CDCl_3_) *δ* 211.5, 142.4, 132.3, 130.7, 130.3, 130.1, 128.2, 59.9, 51.8, 48.5, 38.7, 38.6, 32.8, 32.5, 31.9, 29.4, 22.2. LC-MS (ESI^+^) *m*/*z* calcd. for C_17_H_21_Cl_2_NO [(M + H)]^+^: 326.11; found 326.00. HPLC *t*_R_: 8.533 min.

##### *N*-(2-(3,4-Dichlorophenyl)cyclopentyl)aniline (11a)

Final compound 11a was obtained following general procedure A with a 1.2 : 1 : 3 : 2 stoichiometry of intermediate 8, aniline, acetic acid and NaBH(OAc)_3_, respectively. Flash column chromatography with 10 to 30% DCM in PE as mobile phase gave 11a as a brown oil (67.4 mg, 0.22 mmol, 19%). ^1^H NMR (400 MHz, CDCl_3_) *δ* 7.30 (d, *J* = 8.3 Hz, 1H), 7.27–7.23 (d, *J* = 1.14 Hz, 1H), 7.16–7.05 (m, 2H), 6.98 (ddd, *J* = 8.3, 2.1, 0.8 Hz, 1H), 6.66 (tt, *J* = 7.4, 1.1 Hz, 1H), 6.51–6.42 (m, 2H), 4.01 (q, *J* = 6.2 Hz, 1H), 3.39 (q, *J* = 7.5 Hz, 1H), 3.27 (br s, 1H), 2.25–1.87 (m, 4H), 1.87–1.76 (m, 1H), 1.76–1.66 (m, 1H). ^13^C NMR (101 MHz, CDCl_3_) *δ* 147.4, 141.5, 132.3, 130.6, 130.4, 130.1, 129.3, 128.3, 117.4, 113.3, 57.6, 47.1, 31.9, 29.1, 21.8. LC-MS (ESI^+^) *m*/*z* calcd. for C_17_H_17_Cl_2_N [(M + H)]^+^: 306.08; found 306.00. HPLC *t*_R_: 8.704 min.

##### 4-Chloro-*N*-(2-(3,4-dichlorophenyl)cyclopentyl)aniline (11b)

Final compound 11b was obtained following general procedure A with a 1 : 2.2 : 4 : 5 stoichiometry of intermediate 8, 4-chloroaniline, acetic acid and NaBH(OAc)_3_, respectively. Flash column chromatography with 10 to 20% DCM in PE as mobile phase gave 11b as a transparent oil (9.1 mg, 0.03 mmol, 3%). ^1^H NMR (400 MHz, CDCl_3_) *δ* 7.31 (d, *J* = 8.3 Hz, 1H), 7.23 (d, *J* = 2.1 Hz, 1H), 7.10–7.01 (m, 2H), 6.96 (dd, *J* = 8.3, 2.1 Hz, 1H), 6.43–6.34 (m, 2H), 3.96 (q, *J* = 6.4 Hz, 1H), 3.39 (q, *J* = 7.2 Hz, 1H), 3.26 (br s, 1H), 2.23–1.89 (m, 4H), 1.88–1.74 (m, 1H), 1.73–1.63 (m, 1H). ^13^C NMR (101 MHz, CDCl_3_) *δ* 146.0, 141.3, 132.4, 130.6, 130.2, 129.1, 128.3, 122.0, 114.3, 57.8, 47.0, 31.9, 29.2, 21.8. LC-MS (ESI^+^) *m*/*z* calcd. for C_17_H_16_Cl_3_N [(M + H)]^+^: 340.04; found 340.00. HPLC *t*_R_: 13.326 min.

##### *N*-(2-(3,4-Dichlorophenyl)cyclopentyl)-4-fluoroaniline (11c)

Final compound 11c was obtained following general procedure A with a 1.1 : 1 : 3 : 2 stoichiometry of intermediate 8, 4-fluoroaniline, acetic acid and NaBH(OAc)_3_, respectively. Flash column chromatography with 5 to 10% EtOAc in PE as mobile phase gave 11c as a brown oil (287 mg, 0.89 mmol, 55%). ^1^H NMR (400 MHz, CDCl_3_) *δ* 7.29 (d, *J* = 8.3 Hz, 1H), 7.23 (d, *J* = 2.1 Hz, 1H), 6.97 (ddd, *J* = 8.3, 2.2, 0.7 Hz, 1H), 6.87–6.75 (m, 2H), 6.44–6.30 (m, 2H), 3.94 (q, *J* = 6.3 Hz, 1H), 3.37 (q, *J* = 7.3 Hz, 1H), 3.14 (br s, 1H), 2.21–1.88 (m, 4H), 1.86–1.74 (m, 1H), 1.74–1.61 (m, 1H). ^13^C NMR (101 MHz, CDCl_3_) *δ* 157.0, 154.6, 143.8, 143.8, 141.5, 132.3, 130.6, 130.5, 130.1, 128.3, 115.8, 115.6, 114.1, 114.1, 58.2, 47.2, 31.9, 29.2, 21.9. LC-MS (ESI^+^) *m*/*z* calcd. for C_17_H_16_Cl_2_NF [(M + H)]^+^: 324.07; found 324.00. HPLC *t*_R_: 8.489 min.

##### 2-((2-(3,4-Dichlorophenyl)cyclopentyl)amino)phenol (11d)

Final compound 11d was obtained following general procedure A with a 1 : 1.2 : 3 : 3 stoichiometry of intermediate 8, 2-aminophenol, acetic acid and NaBH(OAc)_3_, respectively. Flash column chromatography with 70 to 100% DCM in PE as mobile phase gave 11d as a green oil (17.5 mg, 0.05 mmol, 5%). ^1^H NMR (400 MHz, CDCl_3_) *δ* 7.23 (d, *J* = 8.3 Hz, 1H), 7.19 (dd, *J* = 2.1, 0.6 Hz, 1H), 6.95 (ddd, *J* = 8.3, 2.2, 0.7 Hz, 1H), 6.78–6.68 (m, 1H), 6.63–6.50 (m, 3H), 4.52 (br s, 1H), 3.91 (s, 1H), 3.29 (q, *J* = 7.5 Hz, 1H), 2.14–1.83 (m, 4H), 1.79–1.58 (m, 2H). ^13^C NMR (101 MHz, CDCl_3_) *δ* 144.5, 141.4, 135.8, 132.2, 130.6, 130.3, 130.1, 128.2, 121.5, 118.5, 114.3, 114.0, 58.4, 47.7, 31.9, 29.0, 22.0. LC-MS (ESI^+^) *m*/*z* calcd. for C_17_H_17_Cl_2_NO [(M + H)]^+^: 322.08; found 322.10. HPLC *t*_R_: 12.267 min.

##### 3-((2-(3,4-Dichlorophenyl)cyclopentyl)amino)phenol (11e)

Final compound 11e was obtained following general procedure A with a 1 : 1.2 : 3 : 4 stoichiometry of intermediate 8, 3-aminophenol, acetic acid and NaBH(OAc)_3_, respectively. Flash column chromatography with 0.5 to 1.5% 1 N methanolic NH_3_ in DCM as mobile phase gave 11e as a red oil (56.4 mg, 0.18 mmol, 17%). ^1^H NMR (400 MHz, CDCl_3_) *δ* 7.30 (d, *J* = 8.3 Hz, 1H), 7.24 (d, *J* = 2.1 Hz, 1H), 6.99–6.92 (m, 2H), 6.12 (ddd, *J* = 8.0, 2.4, 0.8 Hz, 1H), 6.05 (ddd, *J* = 8.2, 2.2, 0.8 Hz, 1H), 5.98 (t, *J* = 2.3 Hz, 1H), 4.80 (br s, 1H), 3.96 (q, *J* = 6.3 Hz, 1H), 3.38 (q, *J* = 7.1 Hz, 1H), 3.29 (br s, 1H), 2.25–1.87 (m, 4H), 1.86–1.60 (m, 2H). ^13^C NMR (101 MHz, CDCl_3_) *δ* 156.7, 149.0, 141.4, 132.3, 130.5, 130.4, 130.2, 130.1, 128.4, 106.5, 104.4, 100.0, 57.6, 47.0, 31.8, 29.0, 21.7. LC-MS (ESI^+^) *m*/*z* calcd. for C_17_H_17_Cl_2_NO [(M + H)]^+^: 322.08; found 322.05. HPLC *t*_R_: 11.673 min.

##### 4-((2-(3,4-Dichlorophenyl)cyclopentyl)amino)phenol (11f)

Final compound 11f was obtained following general procedure A with a 1.1 : 1 : 3 : 2 stoichiometry of intermediate 8, 4-aminophenol, acetic acid and NaBH(OAc)_3_, respectively. Flash column chromatography with 2% MeOH + 1% Et_3_N in DCM as mobile phase gave 11f as a brown oil (166 mg, 0.52 mmol, 35%). ^1^H NMR (400 MHz, CDCl_3_) *δ* 7.30 (d, *J* = 8.3 Hz, 1H), 7.23 (dd, *J* = 2.1, 0.7 Hz, 1H), 6.97 (ddd, *J* = 8.3, 2.1, 0.7 Hz, 1H), 6.63 (d, *J* = 9.0 Hz, 2H), 6.39 (d, *J* = 9.1 Hz, 2H), 3.93 (q, *J* = 6.3 Hz, 1H), 3.36 (q, *J* = 7.2 Hz, 1H), 2.20–1.88 (m, 4H), 1.86–1.74 (m, 1H), 1.73–1.61 (m, 1H). ^13^C NMR (101 MHz, CDCl_3_) *δ* 147.7, 141.7, 141.6, 132.2, 130.7, 130.4, 130.1, 128.3, 116.3, 114.9, 58.6, 47.2, 31.9, 29.2, 21.9. LC-MS (ESI^+^) *m*/*z* calcd. for C_17_H_17_Cl_2_NO [(M + H)]^+^: 322.08; found 322.00. HPLC *t*_R_: 9.829 min.

##### *N*-(2-(3,4-Dichlorophenyl)cyclopentyl)-4-methoxyaniline (11g)

Final compound 11g was obtained following general procedure A with a 1.1 : 1 : 3 : 2 stoichiometry of intermediate 8, 4-methoxyaniline, acetic acid and NaBH(OAc)_3_, respectively. Flash column chromatography with 10 to 30% DCM in PE as mobile phase gave 11g as a brown oil (280 mg, 0.83 mmol, 68%). ^1^H NMR (400 MHz, CDCl_3_) *δ* 7.30 (d, *J* = 8.3 Hz, 1H), 7.24 (dd, *J* = 2.1, 0.7 Hz, 1H), 6.98 (ddd, *J* = 8.2, 2.1, 0.7 Hz, 1H), 6.77–6.66 (m, 2H), 6.47–6.39 (m, 2H), 3.95 (q, *J* = 6.2 Hz, 1H), 3.73 (s, 3H), 3.37 (q, *J* = 7.2 Hz, 1H), 3.01 (br s, 1H), 2.21–1.88 (m, 4H), 1.86–1.75 (m, 1H), 1.74–1.63 (m, 1H). ^13^C NMR (101 MHz, CDCl_3_) *δ* 152.1, 141.7, 141.7, 132.2, 130.7, 130.4, 130.0, 128.4, 115.0, 114.7, 58.5, 55.9, 47.2, 31.9, 29.2, 21.9. LC-MS (ESI^+^) *m*/*z* calcd. for C_18_H_19_Cl_2_NO [(M + H)]^+^: 336.09; found 336.05. HPLC *t*_R_: 7.644 min.

##### *N* ^1^-(2-(3,4-Dichlorophenyl)cyclopentyl)benzene-1,3-diamine (11h)

Final compound 11h was obtained following general procedure A with a 1 : 1.6 : 1 : 4 stoichiometry of intermediate 8, *tert*-butyl (3-aminophenyl)carbamate, acetic acid and NaBH(OAc)_3_, respectively, without purification. Subsequently, the formed Boc-protected amine was dissolved in DCM (0.1 M) and allowed to cool down to 0 °C after which TFA (20 equiv.) was added dropwise. The mixture was stirred for 2 h, concentrated *in vacuo* and co-evaporated with toluene to remove the excess TFA. Automatic column chromatography with 0 to 1% MeOH in DCM as mobile phase on Biotage® Isolera™ One provided final compound 11h as a dark green oil (60.3 mg, 0.19 mmol, 32%). ^1^H NMR (400 MHz, CDCl_3_) *δ* 7.29 (d, *J* = 8.3 Hz, 1H), 7.24 (d, *J* = 2.1 Hz, 1H), 6.97 (dd, *J* = 8.3, 2.1 Hz, 1H), 6.93 (t, *J* = 7.9 Hz, 1H), 6.17 (d, *J* = 7.8 Hz, 1H), 6.04 (dd, *J* = 8.1, 1.9 Hz, 1H), 6.01 (s, 1H), 4.59 (br s, 3H), 3.96 (q, *J* = 6.3 Hz, 1H), 3.37 (q, *J* = 7.2 Hz, 1H), 2.25–1.87 (m, 4H), 1.86–1.61 (m, 2H). ^13^C NMR (101 MHz, CDCl_3_) *δ* 148.4, 143.4, 141.4, 132.3, 130.5, 130.4, 130.3, 130.1, 128.4, 107.0, 106.7, 101.9, 57.7, 47.0, 31.8, 29.0, 21.7. LC-MS (ESI^+^) *m*/*z* calcd. for C_17_H_18_Cl_2_N_2_ [(M + H)]^+^: 321.09; found: 321.05. HPLC *t*_R_: 9.228 min.

##### *N* ^1^-(2-(3,4-Dichlorophenyl)cyclopentyl)benzene-1,4-diamine (11i)

Final compound 11i was obtained following general procedure A with a 1 : 1.6 : 1 : 4 stoichiometry of intermediate 8, benzene-1,4-diamine, acetic acid and NaBH(OAc)_3_, respectively. Automatic column chromatography (C18) with 10 to 90% CH_3_CN in H_2_O + 0.1% TFA as mobile phase on Biotage® Selekt gave 11i as a brown oil (153 mg, 0.48 mmol, 36%). ^1^H NMR (400 MHz, CDCl_3_) *δ* 7.29 (d, *J* = 8.3 Hz, 1H), 7.24 (dd, *J* = 2.1, 0.7 Hz, 1H), 6.97 (ddd, *J* = 8.3, 2.2, 0.7 Hz, 1H), 6.59–6.51 (m, 2H), 6.38–6.32 (m, 2H), 3.91 (q, *J* = 6.3 Hz, 1H), 3.35 (q, *J* = 7.2 Hz, 1H), 3.09 (br s, 3H), 2.21–1.87 (m, 4H), 1.84–1.60 (m, 2H). ^13^C NMR (101 MHz, CDCl_3_) *δ* 141.7, 140.5, 137.7, 132.1, 130.6, 130.2, 130.0, 128.3, 116.9, 115.1, 58.6, 47.1, 31.8, 29.1, 21.8. LC-MS (ESI^+^) *m*/*z* calcd. for C_17_H_18_Cl_2_N_2_ [(M + H)]^+^: 321.09; found: 321.05. HPLC *t*_R_: 7.912 min.

##### *N* ^1^-(2-(3,4-Dichlorophenyl)cyclopentyl)-*N*^3^-methylbenzene-1,3-diamine (11j)

Final compound 11j was obtained following general procedure A with a 1 : 1.2 : 1 : 5 stoichiometry of intermediate 8, *N*^1^-methylbenzene-1,3-diamine, acetic acid and NaBH(OAc)_3_, respectively. Flash column chromatography with 60 to 100% DCM in PE as mobile phase gave 11j as a brown oil (43.2 mg, 0.13 mmol, 13%). ^1^H NMR (400 MHz, CDCl_3_) *δ* 7.31 (d, *J* = 8.3 Hz, 1H), 7.26 (d, *J* = 2.1 Hz, 1H), 7.00 (dd, *J* = 8.3, 2.1, 0.7 Hz, 1H), 6.94 (p, *J* = 4.0 Hz, 1H), 5.98 (dd, *J* = 8.0, 0.9 Hz, 1H), 5.90 (dd, *J* = 8.0, 1.9 Hz, 1H), 5.72 (t, *J* = 1.9 Hz, 1H), 3.99 (q, *J* = 6.2 Hz, 1H), 3.37 (q, *J* = 7.2 Hz, 1H), 2.76 (s, 3H), 2.21–2.10 (m, 1H), 2.09–1.89 (m, 3H), 1.86–1.63 (m, 2H). ^13^C NMR (101 MHz, CDCl_3_) *δ* 150.6, 150.5, 148.6, 148.6, 141.6, 132.2, 130.6, 130.3, 130.1, 130.0, 130.0, 129.9, 129.9, 128.4, 103.2, 102.8, 97.4, 97.3, 57.6, 47.1, 31.9, 30.9, 28.9, 21.7. LC-MS (ESI^+^) *m*/*z* calcd. for C_18_H_20_Cl_2_N_2_ [(M + H)]^+^: 335.11; found: 335.00. HPLC *t*_R_: 9.416 min.

##### *N* ^1^-(2-(3,4-Dichlorophenyl)cyclopentyl)-*N*^4^-methylbenzene-1,4-diamine (11k)

Final compound 11k was obtained following general procedure A with a 1 : 1.2 : 1 : 4 stoichiometry of intermediate 8, *N*^1^-methylbenzene-1,4-diamine, acetic acid and NaBH(OAc)_3_, respectively. Flash column chromatography with 0 to 0.4% MeOH in DCM as mobile phase gave 11k as a yellow oil (31.0 mg, 0.09 mmol, 9%). ^1^H NMR (400 MHz, CDCl_3_) *δ* 7.30 (d, *J* = 8.3 Hz, 1H), 7.25 (dd, *J* = 2.1, 0.7 Hz, 1H), 6.99 (ddd, *J* = 8.3, 2.1, 0.7 Hz, 1H), 6.55–6.50 (m, 2H), 6.46–6.39 (m, 2H), 3.93 (bri s, 1H), 3.36 (q, *J* = 7.5 Hz, 1H), 2.78 (br s, 5H), 2.30–1.88 (m, 4H), 1.86–1.55 (m, 2H). ^13^C NMR (101 MHz, CDCl_3_) *δ* 141.7, 132.2, 130.7, 130.3, 130.0, 128.4, 115.3, 114.5, 58.7, 47.3, 32.1, 31.9, 29.1, 21.8. LC-MS (ESI^+^) *m*/*z* calcd. for C_17_H_18_Cl_2_N_2_ [(M + H)]^+^: 335.11; found: 335.05. HPLC *t*_R_: 8.123 min.

##### 4-((2-(3,4-Dichlorophenyl)cyclopentyl)amino)-2-methylphenol (11l)

Final compound 11l was obtained following general procedure A with a 1 : 1.2 : 1 : 5 stoichiometry of intermediate 8, 4-amino-2-methylphenol, acetic acid and NaBH(OAc)_3_, respectively. Flash column chromatography with 0 to 1% MeOH in DCM as mobile phase gave 11l as a red oil (25.5 mg, 0.08 mmol, 8%). ^1^H NMR (400 MHz, CDCl_3_) *δ* 7.30 (d, *J* = 8.3 Hz, 1H), 7.24 (d, *J* = 2.1 Hz, 1H), 6.99 (dd, *J* = 8.4, 2.1 Hz, 1H), 6.57 (d, *J* = 8.3 Hz, 1H), 6.31–6.20 (m, 2H), 3.93 (q, *J* = 6.3 Hz, 1H), 3.36 (q, *J* = 7.2 Hz, 1H), 2.14 (s, 3H), 2.11–1.87 (m, 4H), 1.87–1.54 (m, 2H). ^13^C NMR (101 MHz, CDCl_3_) *δ* 146.3, 141.6, 141.2, 132.2, 130.7, 130.4, 130.0, 128.3, 124.9, 117.0, 115.9, 112.4, 58.8, 47.2, 31.8, 29.1, 21.8, 16.2. LC-MS (ESI^+^) *m*/*z* calcd. for C_18_H_19_Cl_2_NO [(M + H)]^+^: 336.09; found: 336.05. HPLC *t*_R_: 10.009 min.

##### 4-((2-(3,4-Dichlorophenyl)cyclopentyl)amino)-2-methoxyphenol (11m)

Final compound 11m was obtained following general procedure A with a 1 : 1.4 : 1 : 5 stoichiometry of intermediate 8, 4-amino-2-methylphenol, acetic acid and NaBH(OAc)_3_, respectively. Flash column chromatography with 80 to 100% DCM in PE as mobile phase gave 11m as a brown oil (28.8 mg, 0.08 mmol, 8%). ^1^H NMR (400 MHz, CDCl_3_) *δ* 7.31 (d, *J* = 8.3 Hz, 1H), 7.24 (dd, *J* = 2.2, 0.6 Hz, 1H), 7.00 (ddd, *J* = 8.3, 2.1, 0.7 Hz, 1H), 6.71 (d, *J* = 8.4 Hz, 1H), 6.04 (dd, *J* = 8.4, 2.6 Hz, 1H), 5.98 (d, *J* = 2.5 Hz, 1H), 5.06 (br s, 1H), 3.94 (q, *J* = 6.2 Hz, 1H), 3.77 (s, 3H), 3.36 (q, = 7.2 Hz, 1H), 2.96 (br s, 1H), 2.29–1.86 (m, 4H), 1.85–1.59 (m, 2H). ^13^C NMR (101 MHz, CDCl_3_) *δ* 147.2, 141.7, 141.5, 137.9, 132.2, 130.7, 130.3, 130.1, 128.3, 114.8, 105.4, 98.7, 58.7, 55.9, 47.2, 32.0, 29.2, 21.9. LC-MS (ESI^+^) *m*/*z* calcd. for C_18_H_19_Cl_2_NO_2_ [(M + H)]^+^: 352.09; found: 352.00. HPLC *t*_R_: 10.685 min.

##### 2-Chloro-4-((2-(3,4-dichlorophenyl)cyclopentyl)amino)phenol (11n)

Final compound 11n was obtained following general procedure B in EtOH with a 1 : 1.3 : 8.8 : 7.2 stoichiometry of intermediate 8, 4-amino-2-chlorophenol, acetic acid and NaBH(OAc)_3_, respectively. Automatic column chromatography (C18) with 10 to 70% CH_3_CN in H_2_O + 0.1% TFA as mobile phase on Biotage® Selekt gave 11n as a white solid (TFA salt, 28.6 mg, 0.06 mmol, 12%). ^1^H NMR (400 MHz, MeOD) *δ* 7.41 (d, *J* = 8.3 Hz, 1H), 7.37 (d, *J* = 2.1 Hz, 1H), 7.20 (dd, *J* = 8.3, 2.1 Hz, 1H), 7.00 (d, *J* = 2.6 Hz, 1H), 6.93–6.80 (m, 2H), 4.27 (q, *J* = 6.5 Hz, 1H), 3.49 (q, *J* = 8.7, 6.6 Hz, 1H), 2.31–2.12 (m, 3H), 2.10–1.97 (m, 1H), 1.93–1.74 (m, 2H). ^13^C NMR (101 MHz, MeOD) *δ* 153.3, 140.2, 133.4, 132.2, 131.6, 131.2, 129.6, 123.7, 122.5, 121.7, 118.1, 65.9, 48.1, 30.8, 29.8, 22.8. LC-MS (ESI^+^) *m*/*z* calcd. for C_17_H_16_Cl_3_NO [(M + H)]^+^: 356.04; found: 355.90. HPLC *t*_R_: 12.018 min.

##### 2,6-Dichloro-4-((2-(3,4-dichlorophenyl)cyclopentyl)amino)phenol (11o)

Final compound 11o was obtained following general procedure B in EtOH with a 1 : 1.3 : 8.8 : 2.3 stoichiometry of intermediate 8, 4-amino-2,6-dichlorophenol, acetic acid and NaBH(OAc)_3_, respectively. Automatic column chromatography (C18) with 30 to 70% CH_3_CN in H_2_O + 0.1% TFA as mobile phase on Biotage® Selekt gave 11o as an off-white solid (TFA salt, 42.3 mg, 0.08 mmol, 17%). ^1^H NMR (400 MHz, MeOD) *δ* 7.31 (dd, *J* = 5.2, 3.1 Hz, 2H), 7.10 (dd, *J* = 8.3, 2.1 Hz, 1H), 6.50 (s, 2H), 4.04 (q, *J* = 6.3 Hz, 1H), 3.36 (q, *J* = 7.3 Hz, 1H), 2.25–1.90 (m, 4H), 1.83–1.61 (m, 2H). ^13^C NMR (101 MHz, MeOD) *δ* 142.9, 142.7, 140.5, 132.7, 132.1, 131.1, 130.8, 129.8, 124.3, 115.9, 60.8, 48.9, 32.8, 30.7, 23.1. LC-MS (ESI^+^) *m*/*z* calcd. for C_17_H_15_Cl_4_NO [(M + H)]^+^: 390.00; found: 389.90. HPLC *t*_R_: 12.582 min.

##### 4-((2-(3,4-Dichlorophenyl)cyclopentyl)amino)-2-nitrophenol (11p)

Final compound 11p was obtained following general procedure B in EtOH with a 1 : 1.3 : 8.8 : 7.2 stoichiometry of intermediate 8, 4-amino-2-nitrophenol, acetic acid and NaBH(OAc)_3_, respectively. Automatic column chromatography (C18) with 30 to 70% CH_3_CN in H_2_O + 0.1% TFA as mobile phase on Biotage® Selekt gave 11p as an orange solid (TFA salt, 60.0 mg, 0.12 mmol, 25%). Two stereoisomers in a ratio of 1 : 3 observed on NMR. Only major stereoisomer peaks are reported: ^1^H NMR (400 MHz, MeOD) *δ* 7.39 (dd, *J* = 8.6, 2.7 Hz, 1H), 7.32–7.30 (m, 1H), 7.25 (d, *J* = 8.2 Hz, 1H), 7.21 (d, *J* = 2.7 Hz, 1H), 7.12 (dt, *J* = 8.3, 1.6 Hz, 1H), 6.60 (d, *J* = 8.6 Hz, 1H), 4.14 (q, *J* = 6.0, 5.6 Hz, 1H), 3.42 (q, *J* = 7.4 Hz, 1H), 2.31–2.10 (m, 2H), 2.08–1.90 (m, 2H), 1.85–1.73 (m, 2H). ^13^C NMR (101 MHz, MeOD) *δ* 151.5, 143.2, 142.2, 138.1, 132.6, 131.8, 130.9, 130.7, 129.6, 114.2, 112.6, 105.9, 58.3, 49.2, 33.7, 31.1, 23.5. LC-MS (ESI^+^) *m*/*z* calcd. for C_17_H_16_Cl_2_N_2_O_3_ [(M + H)]^+^: 367.06; found: 367.00. HPLC *t*_R_: 12.123 min.

### 4.2 Molecular pharmacology

#### 4.2.1 Reagents and materials

Jump-In™ T-REx™ human embryonic kidney 293 (HEK293-JumpIn) cells with doxycycline-inducible expression of human NET (HEK293-JumpIn-NET), DAT (HEK293-JumpIn-DAT) or SERT (HEK293-JumpIn-SERT) were kindly provided by the RESOLUTE consortium (http://re-solute.eu). Nisoxetine hydrochloride was purchased from Santa Cruz Biotechnology (Dallas, TX, USA), GBR-12909 dihydrochloride was obtained from Toronto Research Chemicals (Toronto, ON, Canada) and imipramine from Nogepha B.V. (Alkmaar, The Netherlands).

#### 4.2.2 Cell culture

HEK293-JumpIn-NET, -DAT and -SERT cells were grown in Dulbecco's modified Eagles medium (DMEM) supplemented with 10% (v/v) fetal bovine serum (FBS, dialyzed in case of SERT), 2 mM GlutaMAX, penicillin (100 IU mL^−1^) and streptomycin (100 μg mL^−1^) at 37 °C with 5% CO_2_. Cells were subcultured twice weekly at a ratio of 1 : 15. All experiments were performed within 20 passages.

#### 4.2.3 MAT fluorescent neurotransmitter uptake assay

Fluorescent uptake assays were performed using the Neurotransmitter Transporter Uptake Assay Kit (Molecular Devices, San Jose, CA, USA) following the supplier's protocol.^36^ In brief, HEK293-JumpIn-NET, -DAT and -SERT cells were seeded (60 000 cells per well) in a poly-d-lysine coated black 96-well plate and induced with doxycycline (1 μg per mL) to express the transporter of interest for 24 h. Subsequently, medium was removed, and cells were preincubated with either no inhibitor (vehicle), 10 μM reference inhibitor (nisoxetine, GBR-12909 or imipramine for NET, DAT and SERT, respectively) or inhibitor of interest (at 1 μM or increasing concentrations ranging from 10^−11^ to 10^−5^ M) in HBSS containing 20 mM HEPES (pH 7.4 at 25 °C) for 1 h at 37 °C. Next, uptake was initiated by the addition of 100 μM fluorescent neurotransmitter with extracellular quenching dye. Uptake of fluorescent neurotransmitter was continuously monitored by measuring fluorescence (*λ*_ex_ = 440 nm, *λ*_em_ = 520 nm) on the FlexStation 3 Multi-Mode Microplate Reader (Molecular Devices, San Jose, CA, USA) every 25 s for 1 h at 37 °C.

#### 4.2.4 Data analysis

All experimental data were analyzed using GraphPad Prism 10.1.0 (GraphPad Software Inc., San Diego, CA, USA). Data are represented as the mean with individual values between brackets (for *n* = 2) or mean ± SEM (for *n* = 3) where each individual experiments was performed in duplicate.

Fluorescent uptake data were analyzed by subtracting basal fluorescence over time prior to calculating the total area under the curve (AUC) over 60 minutes from the baseline-correct time traces. The AUC of vehicle-treated and reference inhibitor-treated cells was set to 0% and 100% inhibition, respectively.

### 4.3 Molecular docking

The binding pose of compound 11e was analyzed when docked into the recently published high-resolution cryo-EM structure of hDAT bound to cocaine (PDB: 9EO4 (ref. 47)) using GLIDE-SP in Schrödinger's Maestro v2022-3 (Schrödinger, Inc., New York, NY, USA).^18^ The protein was prepared using protein preparation wizard, including an energy minimization step followed by generation of a docking grid around the binding site based on the center of geometry of cocaine and preparation for docking with GLIDE.^48^ Both *cis*-oriented isomers of 11e were used, prepared with LigPrep and consequently docked using GLIDE. Following docking, the results were filtered and analyzed regarding their predicted docking scores and interactions. Docking of both *cis*-oriented isomers resulted in similar interactions and therefore the stereoisomer with the best docking score (1*R*,2*R*-11e) was used. Images were generated using PyMOL version 2.5.2.^49^ Alignment of all published hDAT and hNET cryo-EM structures available (hDAT PDB: 8VBY, 8Y2C, 8Y2D, 8Y2E, 8Y2F, 8Y2G, 9EO4 and hNET PDB: 8HFE, 8HFF, 8HFG, 8HFI, 8HFL, 8I3V, 8WGR, 8WGX, 8WTU, 8WTV, 8WTW, 8WTX, 8WTY, 8XB2, 8XB3, 8XB4, 8Y8Z, 8Y90, 8Y91, 8Y92, 8Y92, 8Y93, 8Y94, 8Y95, 8YR2, 8Z1L) was performed with the align function in PyMOL. Herein, all hDAT structures as well as hNET structure 8HFE were aligned with 9EO4 followed by the alignment of all hNET structures with 8HFE accordingly in order to visualize the flexible loop region between TM6a and TM6b.^18–20,31,32,50,51^

Next, the coordinates of a previously published model of hNET^25^ were used as a starting point for MD simulations. The protein-ligand complex was prepared by docking 1*R*,2*R*-11e to this model and subsequently embedding the protein–ligand complex in a POPC membrane model using Memprot.GPCR-ModSim.^52^ The embedded system was thereafter used for free energy calculations in the MD engine Q^53^ using QligFEP^54^ and QresFEP^55^ protocols. Equilibrated transporter–ligand complexes were transferred to the Q molecular dynamics package and simulated under spherical boundary conditions, using a 50 Å diameter simulation sphere centered on the ligand binding site and treated with the surface-constrained all-atom solvent (SCAAS) model to mimic bulk solvation.^56^ Long-range electrostatics were treated using a local reaction field approach,^57^ with no cutoff applied to atoms undergoing alchemical transformation, and covalent bonds involving hydrogen atoms were constrained using SHAKE^58^ allowing a 2 fs integration time step. Ligand transformations were carried out using the dual-topology QligFEP protocol, while residue perturbations employed the single-topology QresFEP protocol, both using soft-core potentials and linear λ-sampling (typically ∼100 λ-windows for ligand perturbations) with multiple independent replicas. Relative free energies were estimated using the Bennett acceptance ratio (BAR).^59^ Relative free energies were obtained by performing the perturbation in protein and water or holo and apo states for QligFEP and QresFEP respectively.

### 4.4 *Drosophila melanogaster* genetics and DAT inhibitor treatment

The transgenic UAS reporter line for the wild type YFP-tagged human DAT (hDAT) was already available in the laboratory.^45^*Fumin* (DAT-null) flies were obtained from Prof. Dr. Kazuhiko Kume, Nagoya City University, Japan. All flies were kept at 25 °C in a 12 h light/12 h dark cycle on a standard cornmeal medium. Food consumption was measured using the FlyPAD device (http://flypad.rocks), according to the manufacturer's protocol (V2, 2018, Easy Behavior).^60^ Briefly, three- to five-day-old male flies expressing hDAT in a *fumin* background, were starved for 20 to 22 h at 29 °C and received 2 μL of 5 mM glucose (prepared in 1% agarose), supplemented with DMSO (control) or 100 times IC_50_ of compounds in FlyPad arena. Food consumption of individual flies was measured for a period of 1 h at 25 °C and the total number of sips was plotted. Locomotor activity of flies was studied using a protocol described in Kasture *et al.*,^45^ with some modification. Briefly, three- to five-day-old male flies received standard cornmeal medium/food, supplemented with DMSO (control), or 100 times the IC_50_ values for DAT of nisoxetine, GBR-12909, 6, 11e or 11f, for 48 h, and were then transferred to 5 mm diameter polycarbonate tubes which carried food pellet, supplemented with DMSO or the test compounds. Locomotor activity of the flies was determined using the DAM5H activity monitor (Trikinetics, USA). In DAM5H, the flies were kept on a 12 h light/12 h dark cycle for the first two days, and the cycle was shifted to a 12 h dark/12 h dark for four subsequent days. During this period, the fly locomotion activity was monitored at 1 min time intervals, and the data were grouped into 60 min bins. For sleep analysis, data from the second day of the dark/dark phase were used for calculations. The pySolo software was used to quantify fly sleep.^61^ Inactivity of 5 min or more was considered as sleep.

## Author contributions

Conceptualization: M. A. D., A. P. IJ., L. H. H. and D. v. d. E.; investigation: M. A. D., W. J., A. S. K., R. L., T. A. M. M., J. G. v. d. P. and A. M. M. B.; data curation, formal analysis and validation: M. A. D., W. J., A. S. K., R. L., T. A. M. M.; funding resources and supervision: G. J. P. v. W., T. H., S. S., A. P. IJ., L. H. H. and D. v. d. E.; writing – original draft: M. A. D., W. J., A. S. K.; writing – reviewing and editing: M. A. D., W. J., A. S. K., R. L., T. A. M. M., G. J. P. v. W., T. H., S. S., A. P. IJ., L. H. H. and D. v. d. E.

## Conflicts of interest

There is no conflict of interest to declare.

## Supplementary Material

MD-OLF-D6MD00112B-s001

## Data Availability

The data supporting this article have been included as part of the supplementary information (SI). Supplementary information: Fig. S1–S3, NMR spectra and HPLC spectra. See DOI: https://doi.org/10.1039/d6md00112b.
