# Supplementary material for: From dual norepinephrine–dopamine reuptake inhibition to selective dopamine reuptake inhibitors: a case of aromatically substituted N,2-cyclopentylamines
Source: RSC Med Chem. 2026 Jun 24;17(7):3447–62. doi: 10.1039/d6md00112b (PMC13334707; doi:10.1039/d6md00112b)
Supplement: MD-017-D6MD00112B-s001 [file MD-017-D6MD00112B-s001.pdf]

Supplementary Information (SI) for RSC Medicinal Chemistry.
This journal is © The Royal Society of Chemistry 2026

Supporting Information

From dual norepinephrine-dopamine reuptake inhibition to selective dopamine reuptake inhibitors: a case of aromatically substituted *N*,2-cyclopentylamines

Majlen A. Dilweg[a], Willem Jespers[a,e], Ameya S. Kasture[b,c], Rongfang Liu[a], Tamara A. M. Mocking[a], Jelle G. van der Ploeg[a], Adrianus M. M. Baselier[a], Gerard J. P. van Westen[a], Thomas Hummel[c], Sonja Sucic[b], Adriaan P. IJzerman[a], Laura H. Heitman[a,d], Daan van der Es[a]*

[a]Division of Medicinal Chemistry, Leiden Academic Centre for Drug Research, Leiden University, Leiden, The Netherlands

[b]Institute of Pharmacology, Medical University of Vienna, Vienna, Austria

[c]Department of Neurosciences and Developmental Biology, University of Vienna, Vienna, Austria

[d]Oncode Institute, Leiden, The Netherlands

[e]Department of Medicinal Chemistry, Photopharmacology and Imaging, Groningen Research. Institute of Pharmacy (GRIP), Faculty of Science and Engineering, Antonius Deusinglaan 1, 9713 AV Groningen, The Netherlands

*Corresponding author, email: d.van.der.es@lacdr.leidenuniv.nl

Contents

Supplementary figures S2

NMR spectra S4

HPLC spectra S25

Classified as Internal | Intern

Supplementary figures

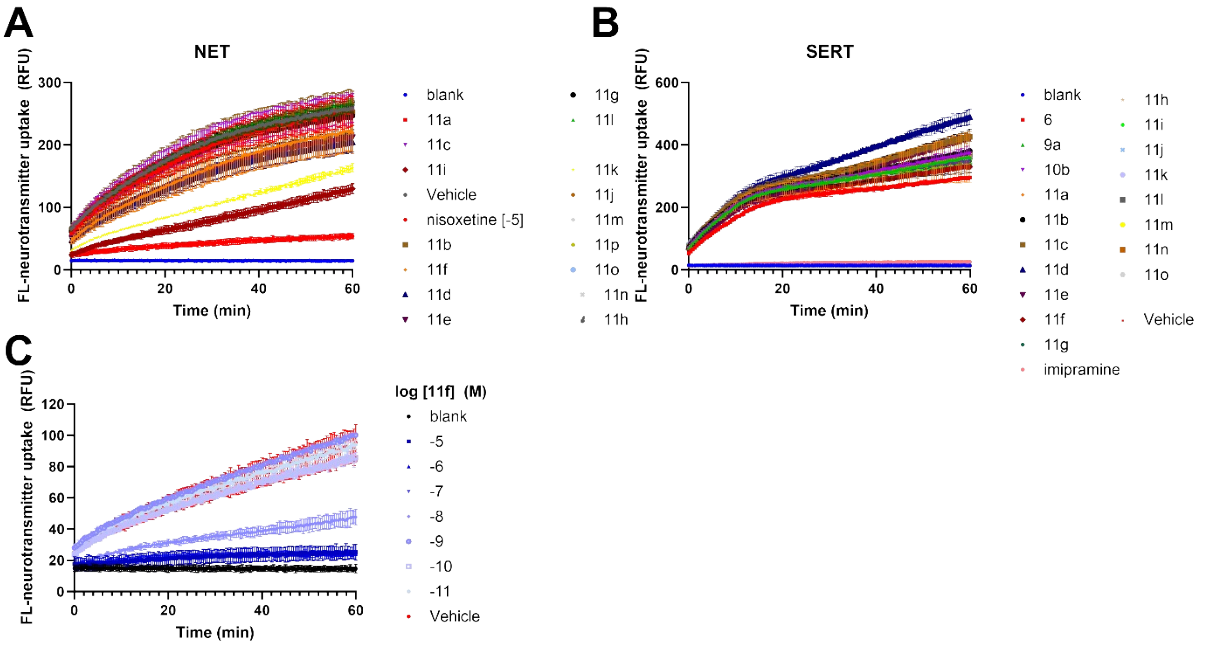

Figure S1. Representative raw time traces of cycloalkylamine-mediated inhibition of fluorescent neurotransmitter uptake. A) NET inhibition by 1µM of the indicated compounds. B) SERT inhibition by 1µM of the indicated compounds. C) DAT inhibition by an ascending concentration of compound **11f**.

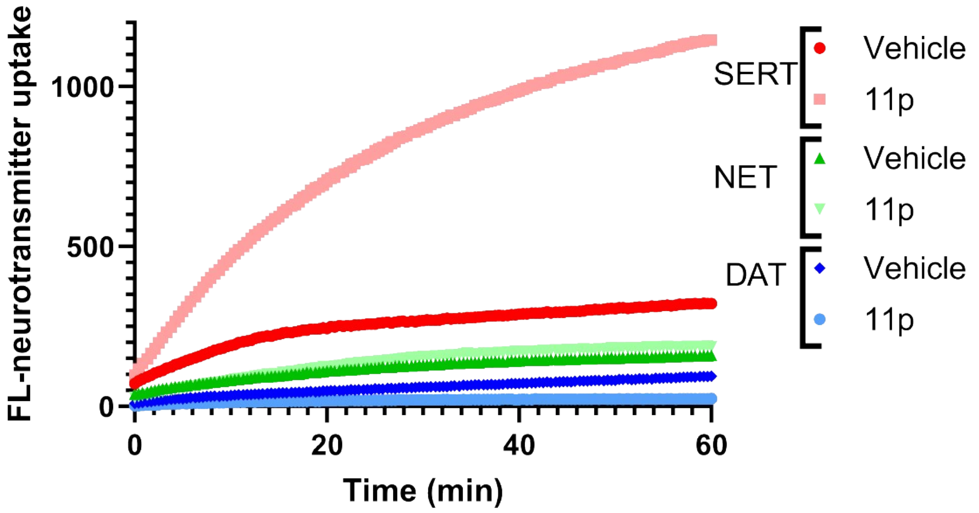

Figure S2. Representative time traces of fluorescent neurotransmitter uptake in absence and presence of 1µM compound **11p** in HEK293-Jumpin cells induced to express SERT, NET or DAT.

Classified as Internal | Intern

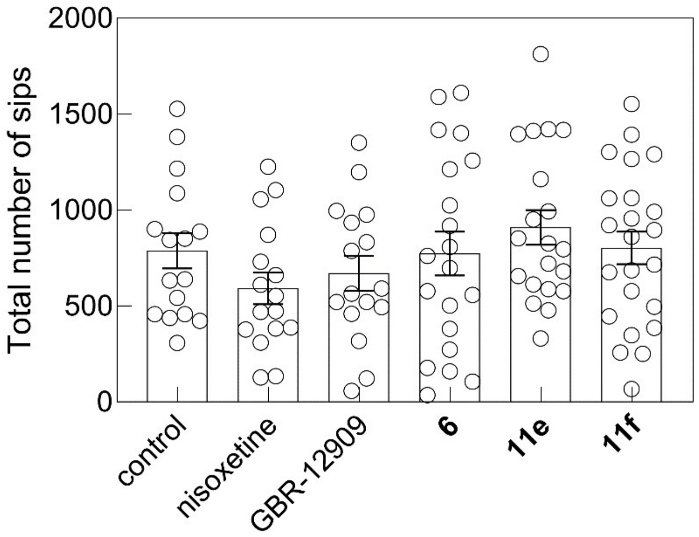

Figure S3. Flies consume food supplemented with cycloalkylamine inhibitor test compounds. Three- to five-day-old male flies expressing human DAT in DAT null background were starved for a period of 20 to 22 h at 29 °C and introduced individually into FlyPad arena. Flies received 2 µL of 5 mM glucose (prepared in 1% agarose) supplemented with DMSO (control) or concentrations corresponding to 100 times the IC_{50} of each compound. Feeding behavior of flies was studied for 1 h at 25 °C and the total number of sips was measured. Circles represent individual flies.

Classified as Internal | Intern

NMR spectra

Compound 7; 1H NMR (CDCl3, 400 MHz)

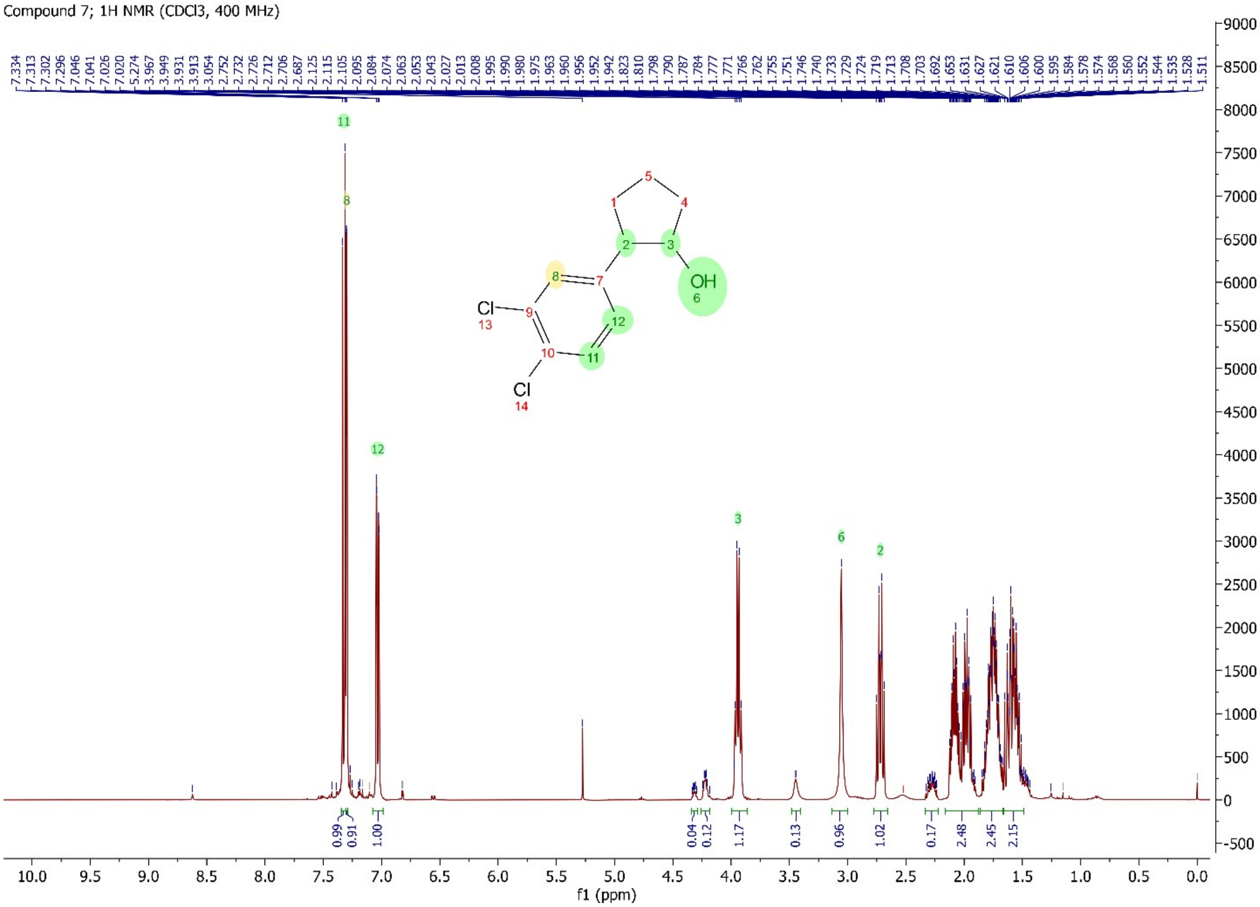

Compound 8; 1H NMR (CDCl3, 400 MHz)

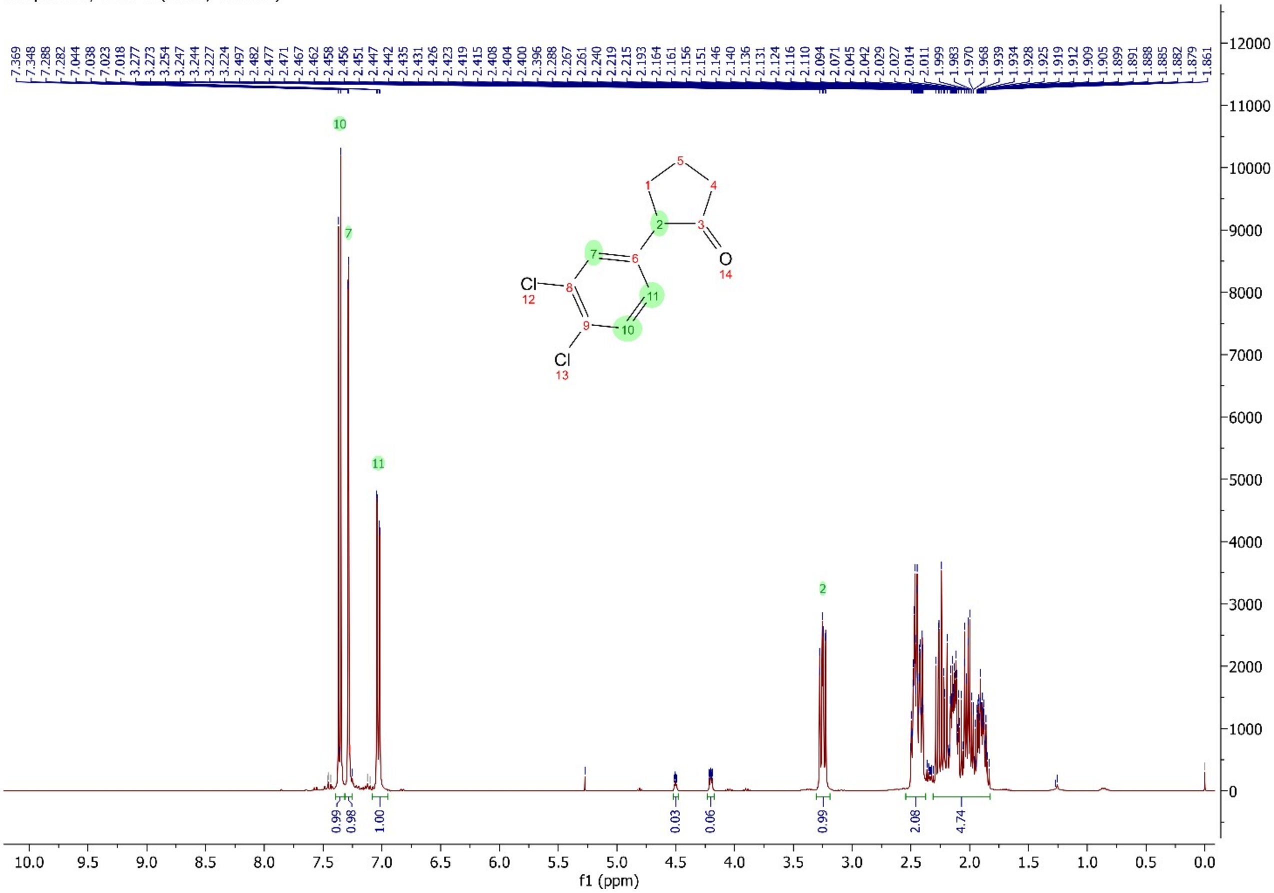

Classified as Internal | Intern

Compound 9a; 1H NMR (CDCl3, 400 MHz)

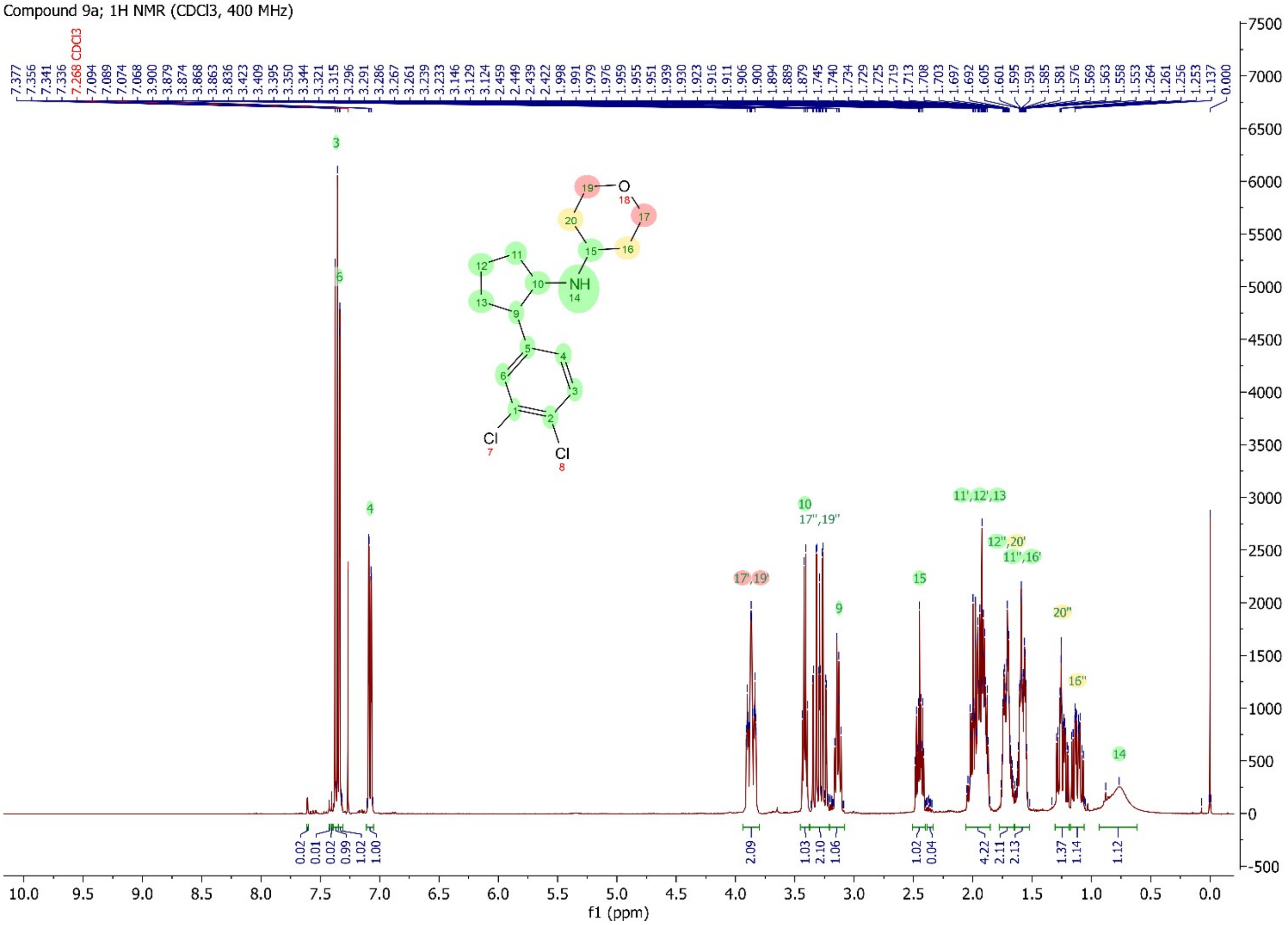

Compound 9a; 13C NMR (CDCl3, 126 MHz)

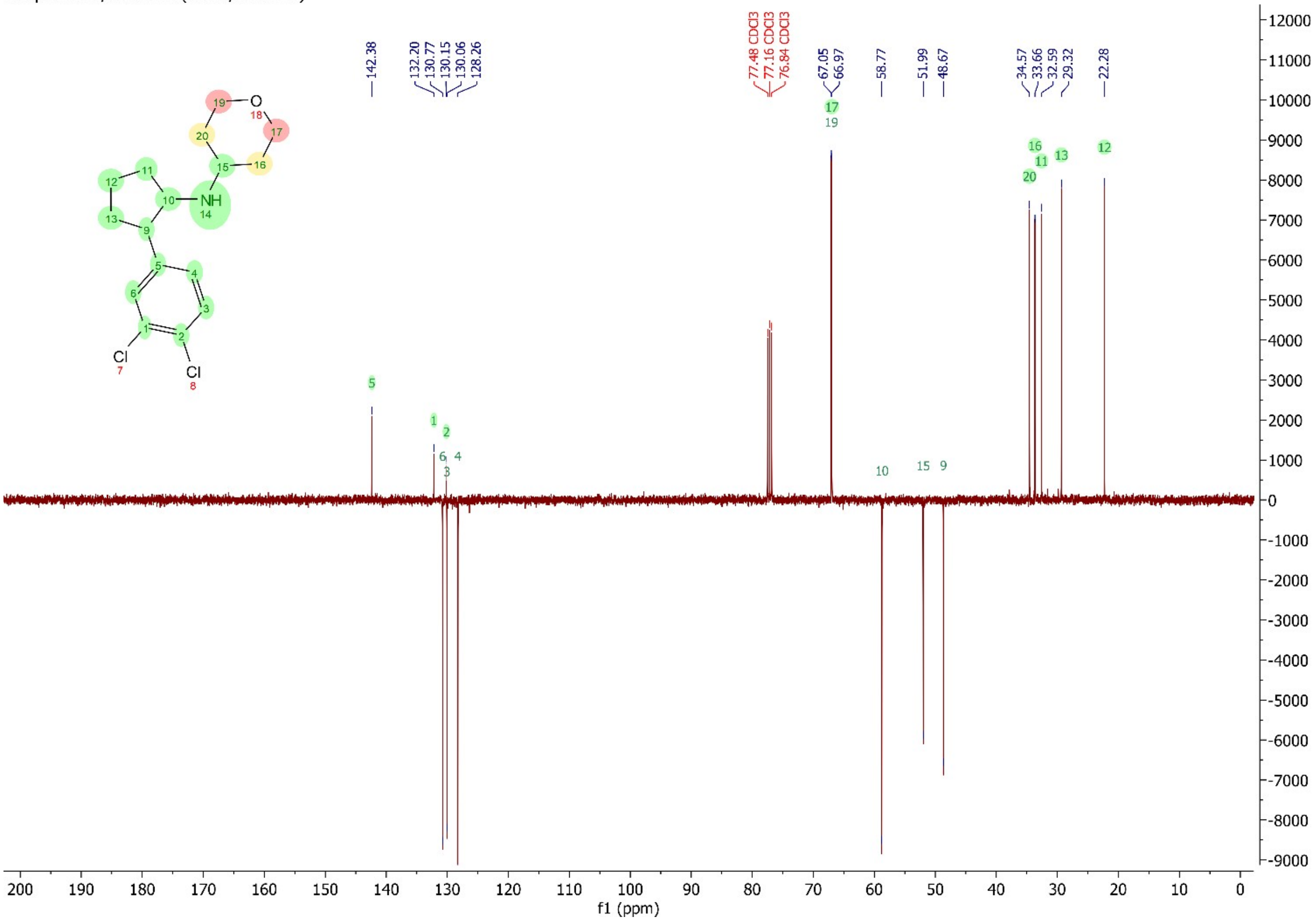

Classified as Internal | Intern

Compound 10b; 1H NMR (CDCl3, 400 MHz)

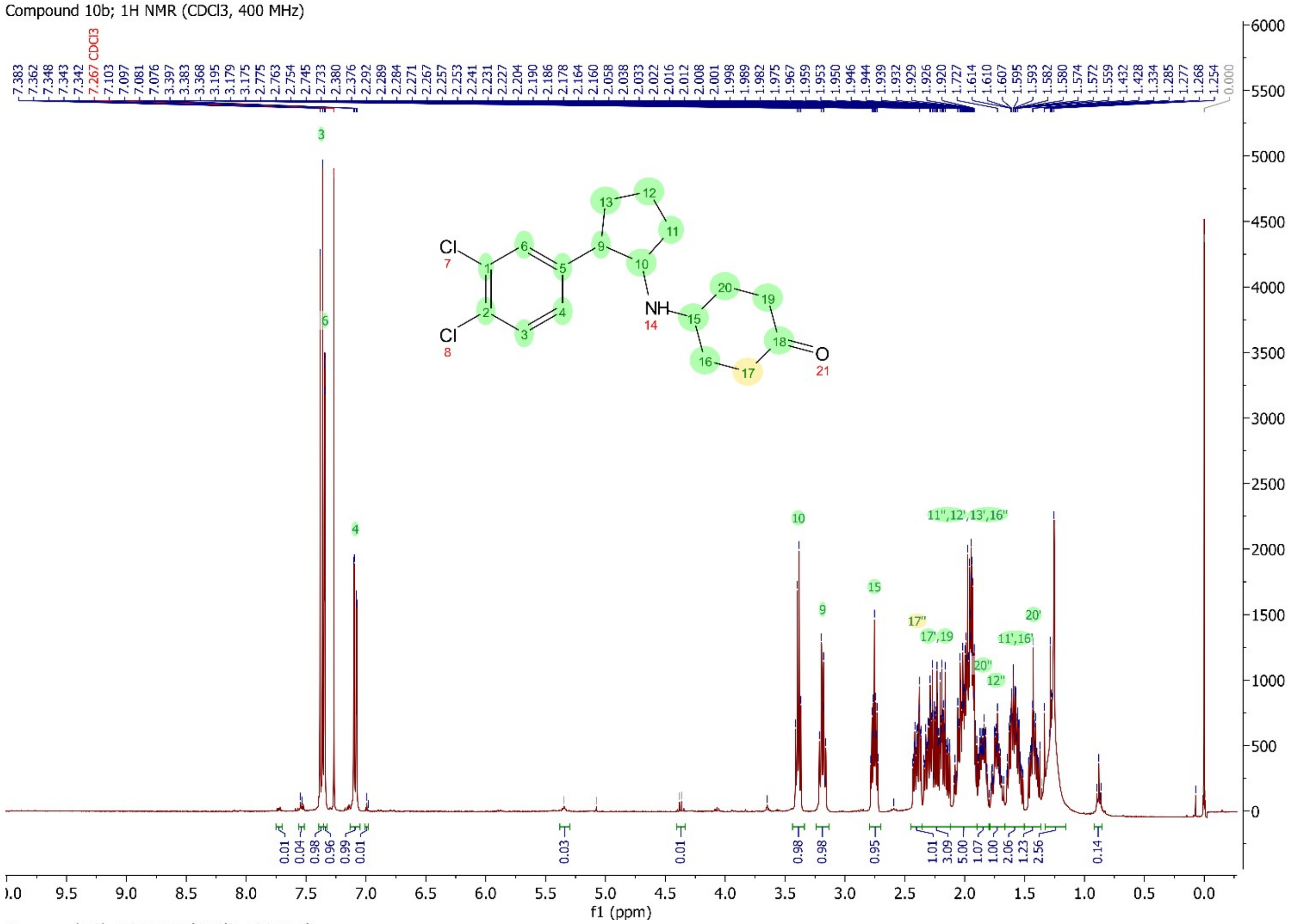

Compound 10b; 13C NMR (CDCl3, 126 MHz)

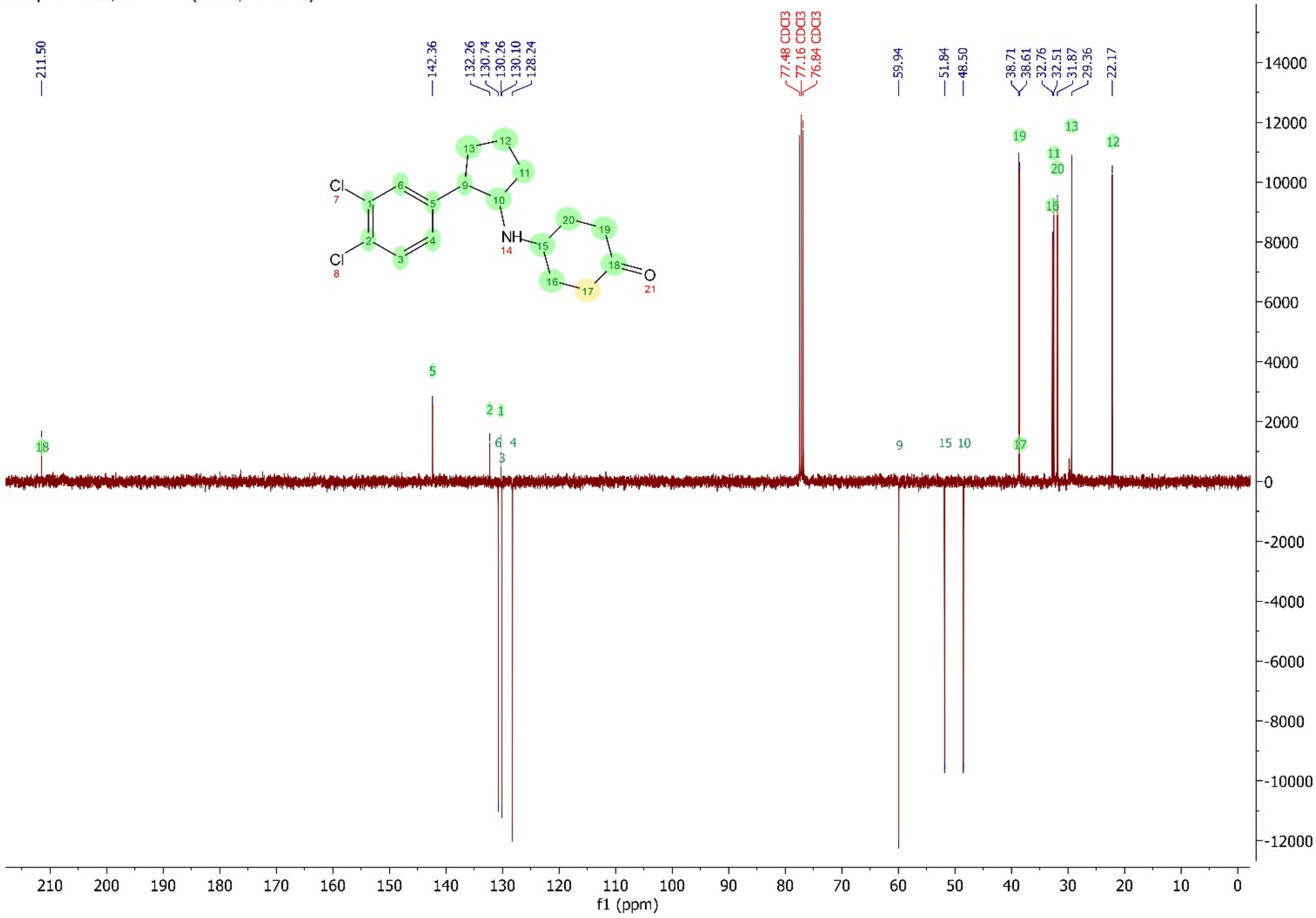

Classified as Internal | Intern

Compound 11a; 1H NMR (CDCl3, 400 MHz)

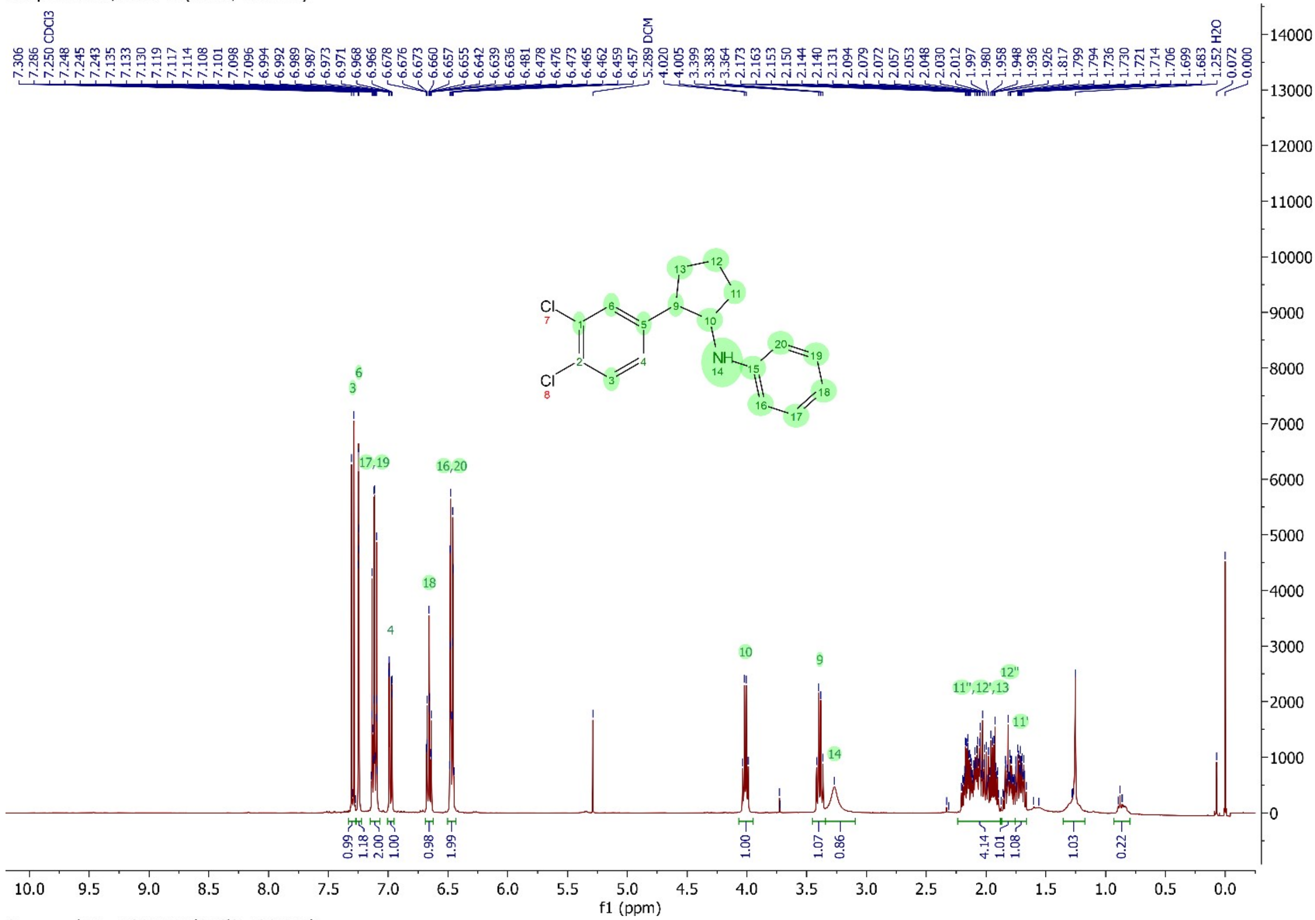

Compound 11a; 13C NMR (CDCl3, 126 MHz)

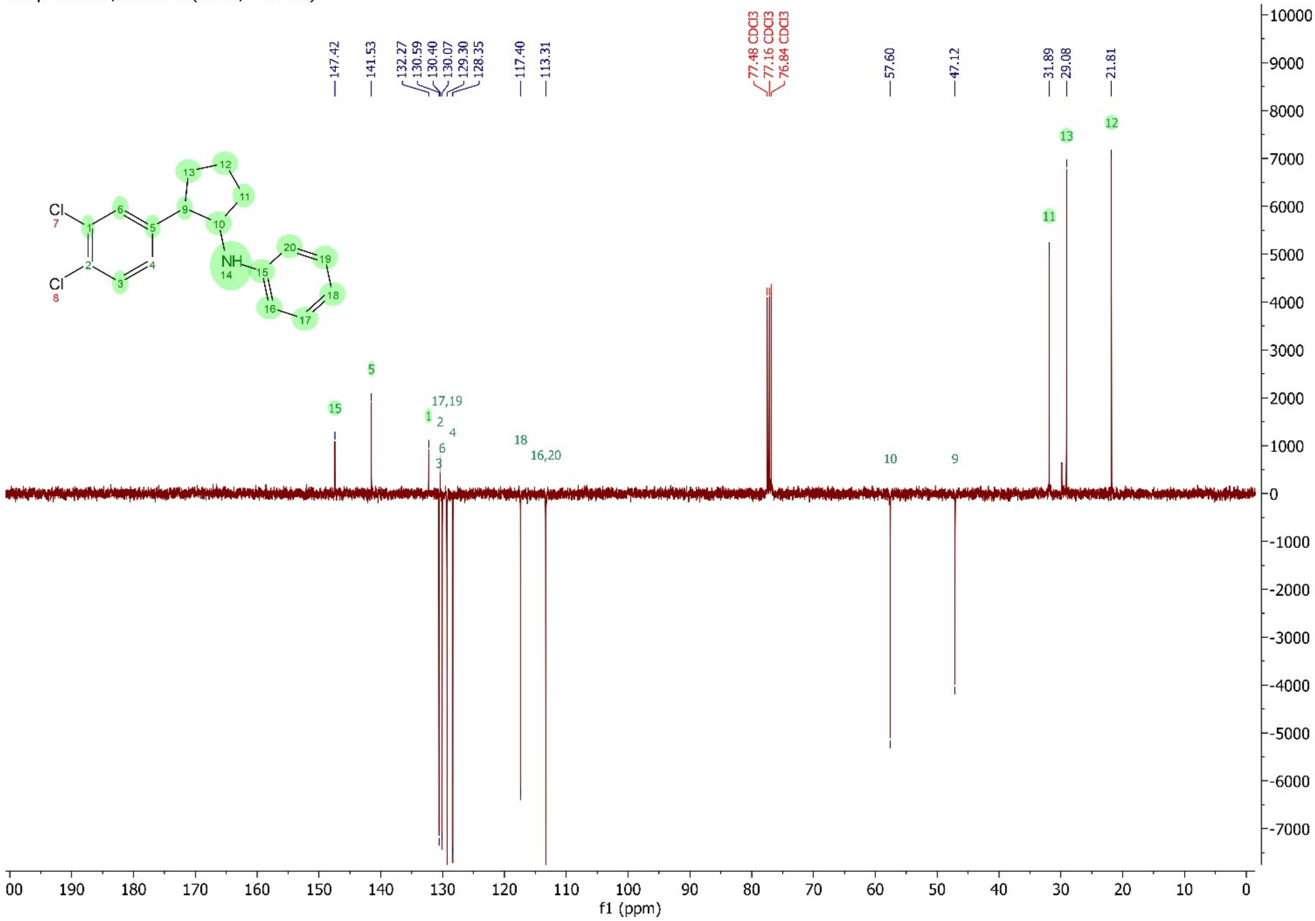

Classified as Internal | Intern

Compound 11b; 1H NMR (CDCl3, 400 MHz)

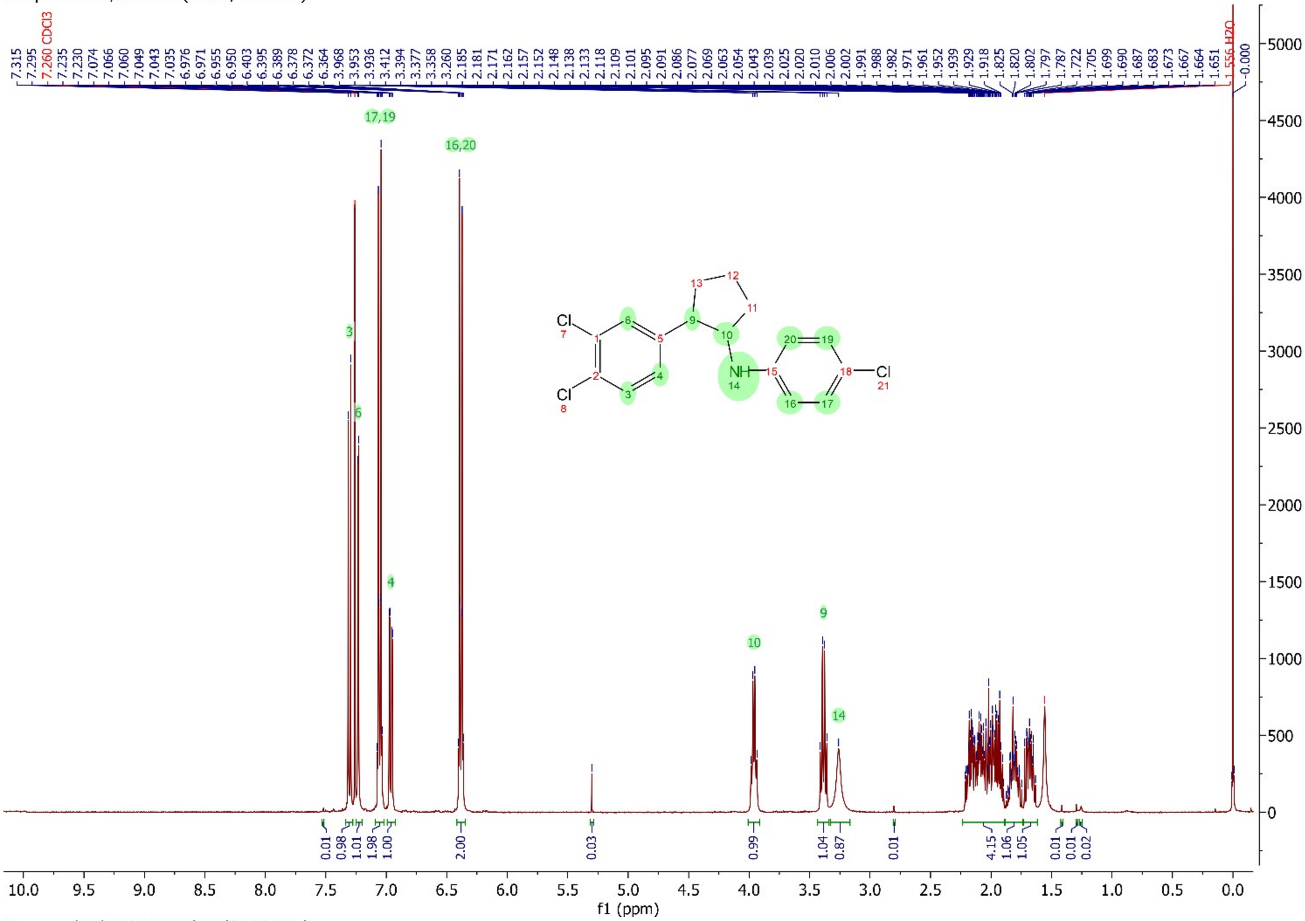

Compound 11b; 13C NMR (CDCl3,126 MHz)

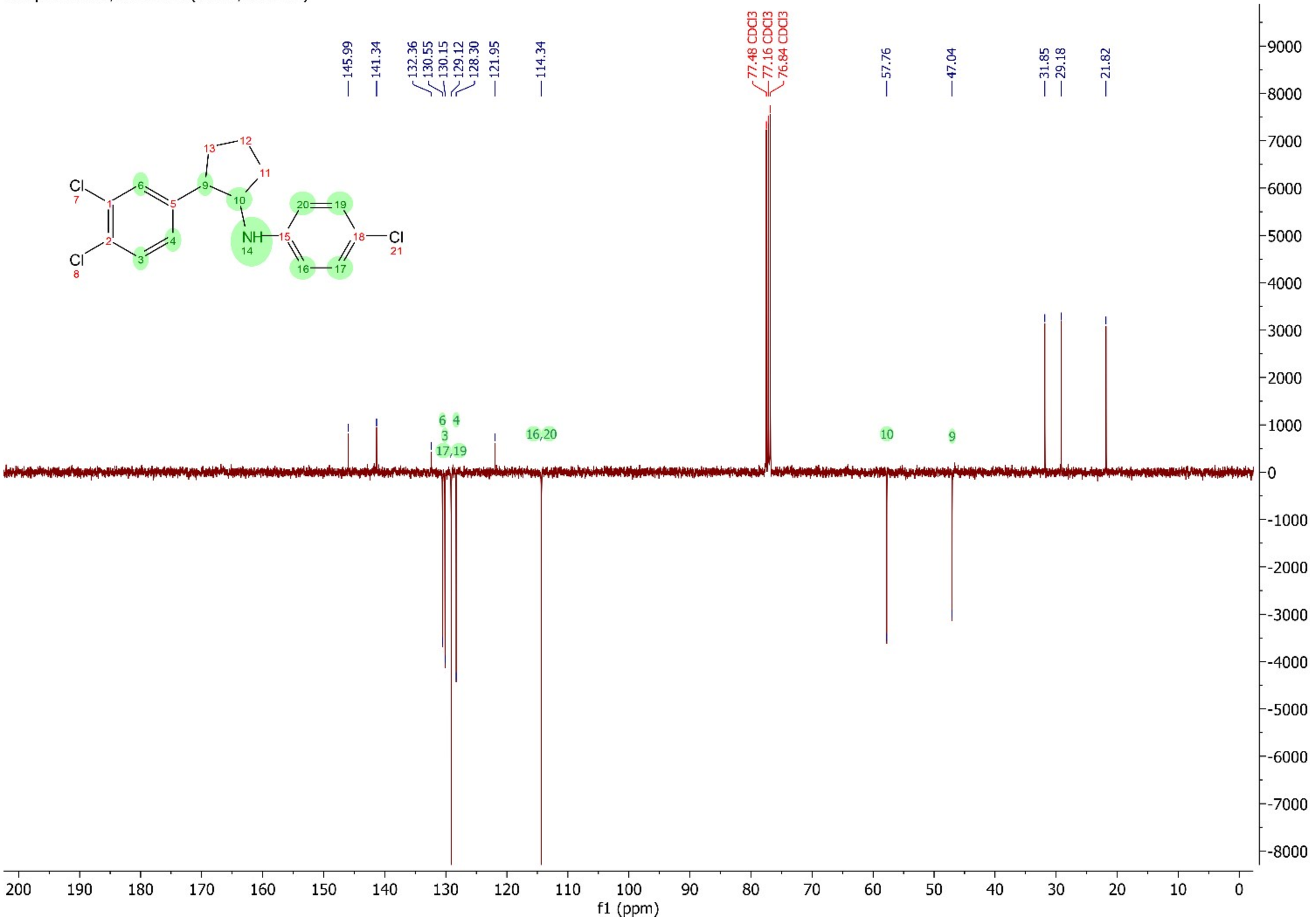

Classified as Internal | Intern

Compound 11c; 1H NMR (CDCl3, 400 MHz)

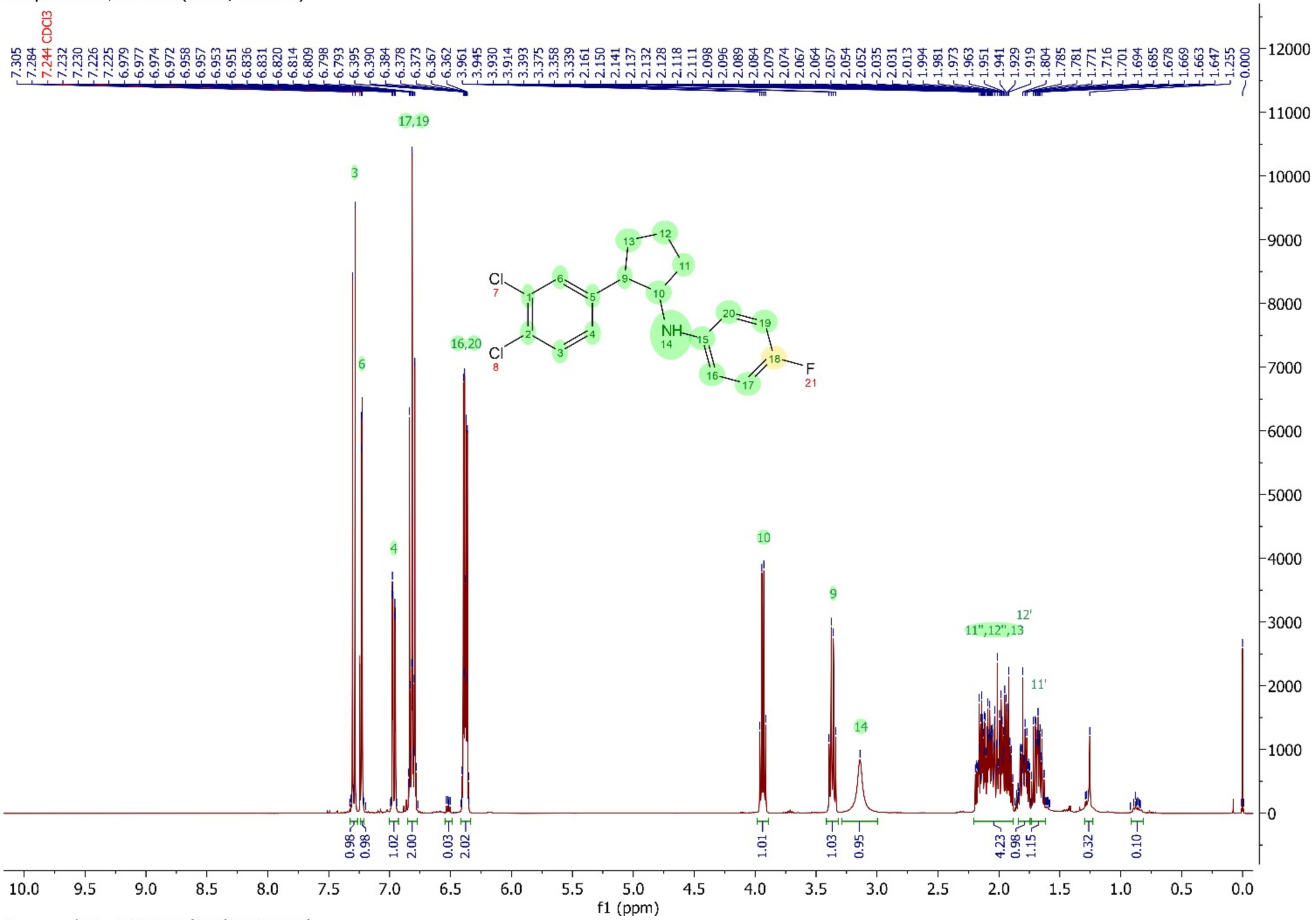

Compound 11c; 13C NMR (CDCl3, 126 MHz)

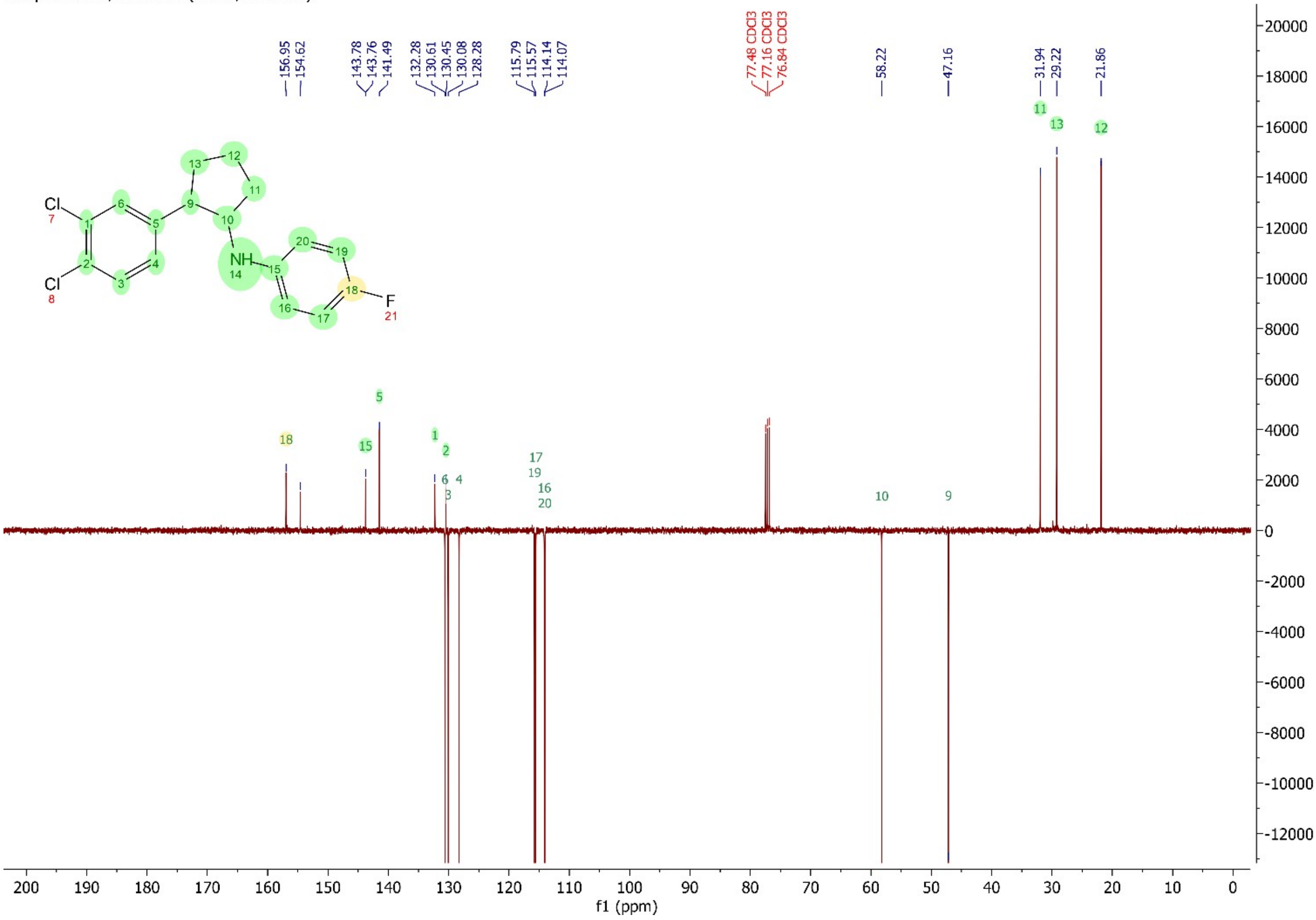

Classified as Internal | Intern

Compound 11d; 1H NMR (CDCl3, 400 MHz)

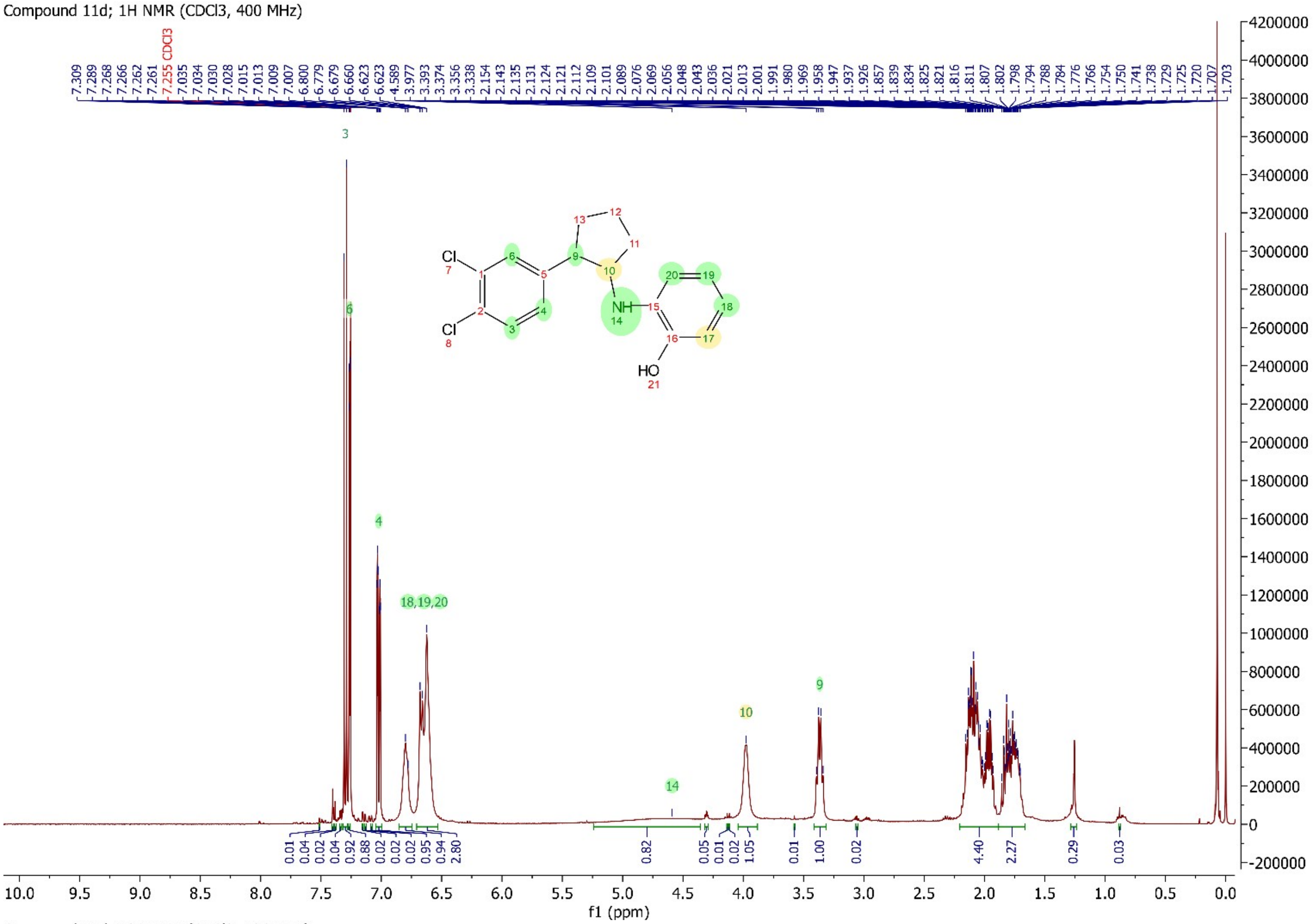

Compound 11d; 13C NMR (CDCl3, 126 MHz)

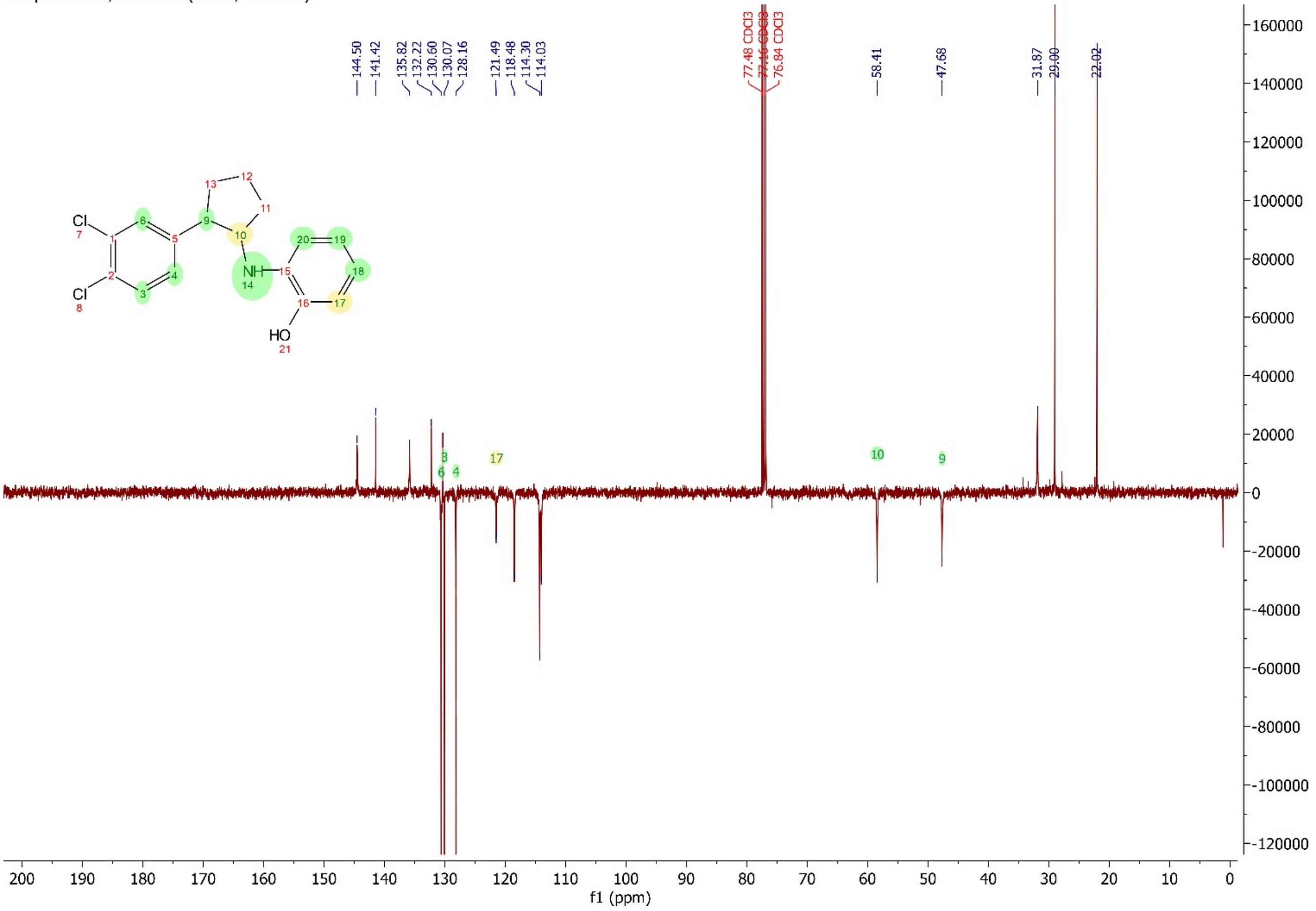

Classified as Internal | Intern

Compound 11e; 1H NMR (CDCl3, 400 MHz)

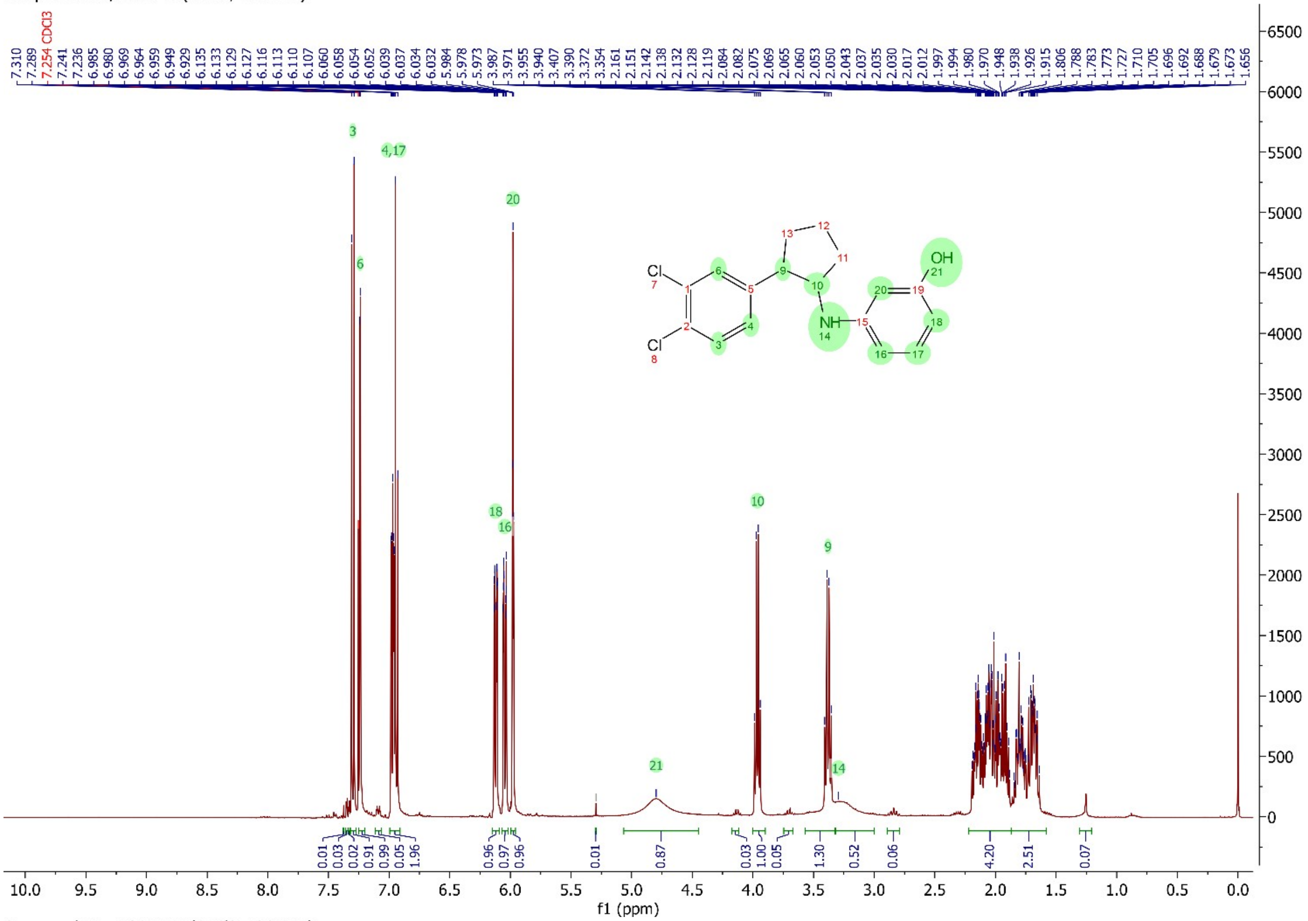

Compound 11e; 13C NMR (CDCl3, 126 MHz)

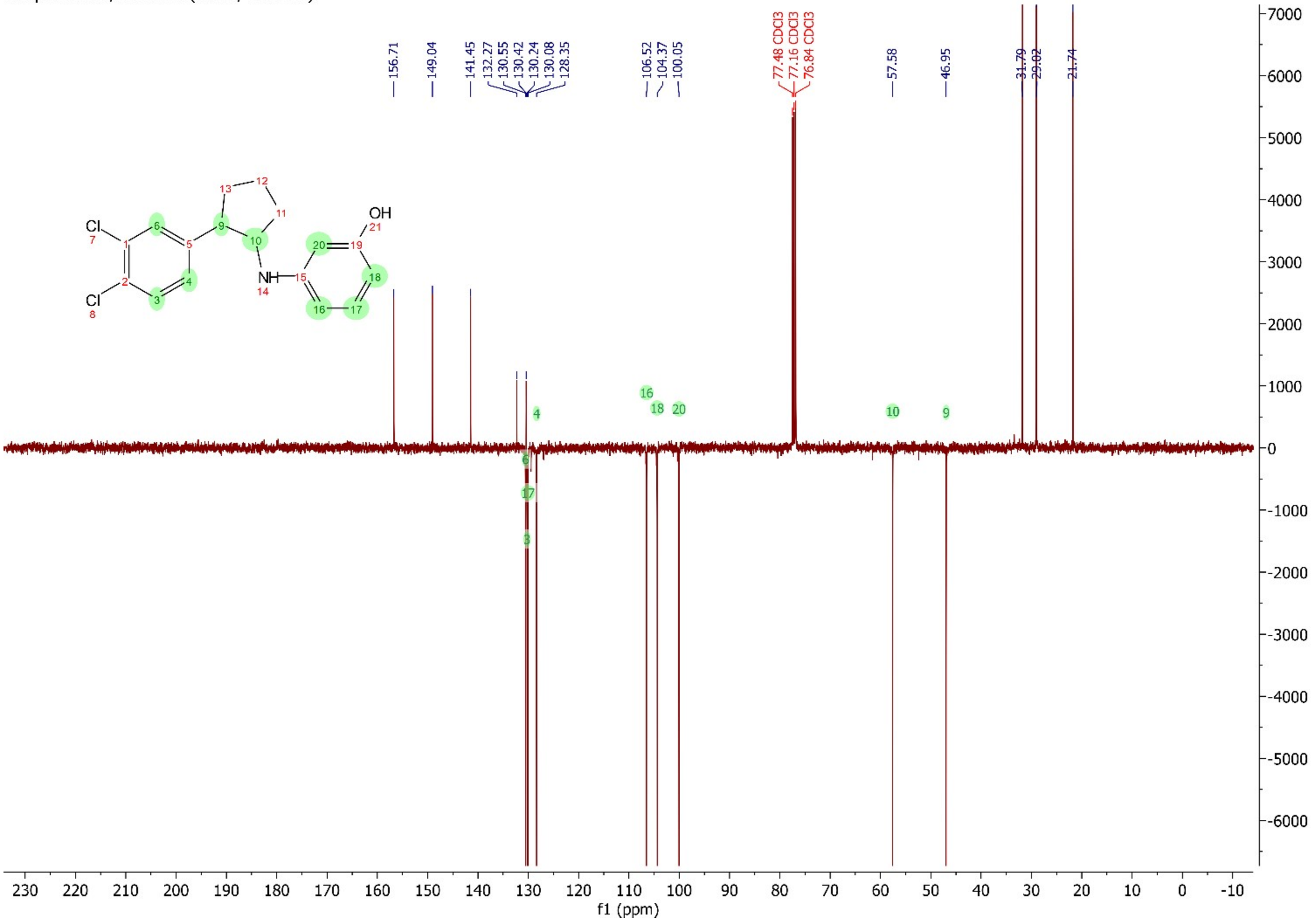

Classified as Internal | Intern

Compound 11f; 1H NMR (CDCl3, 400 MHz)

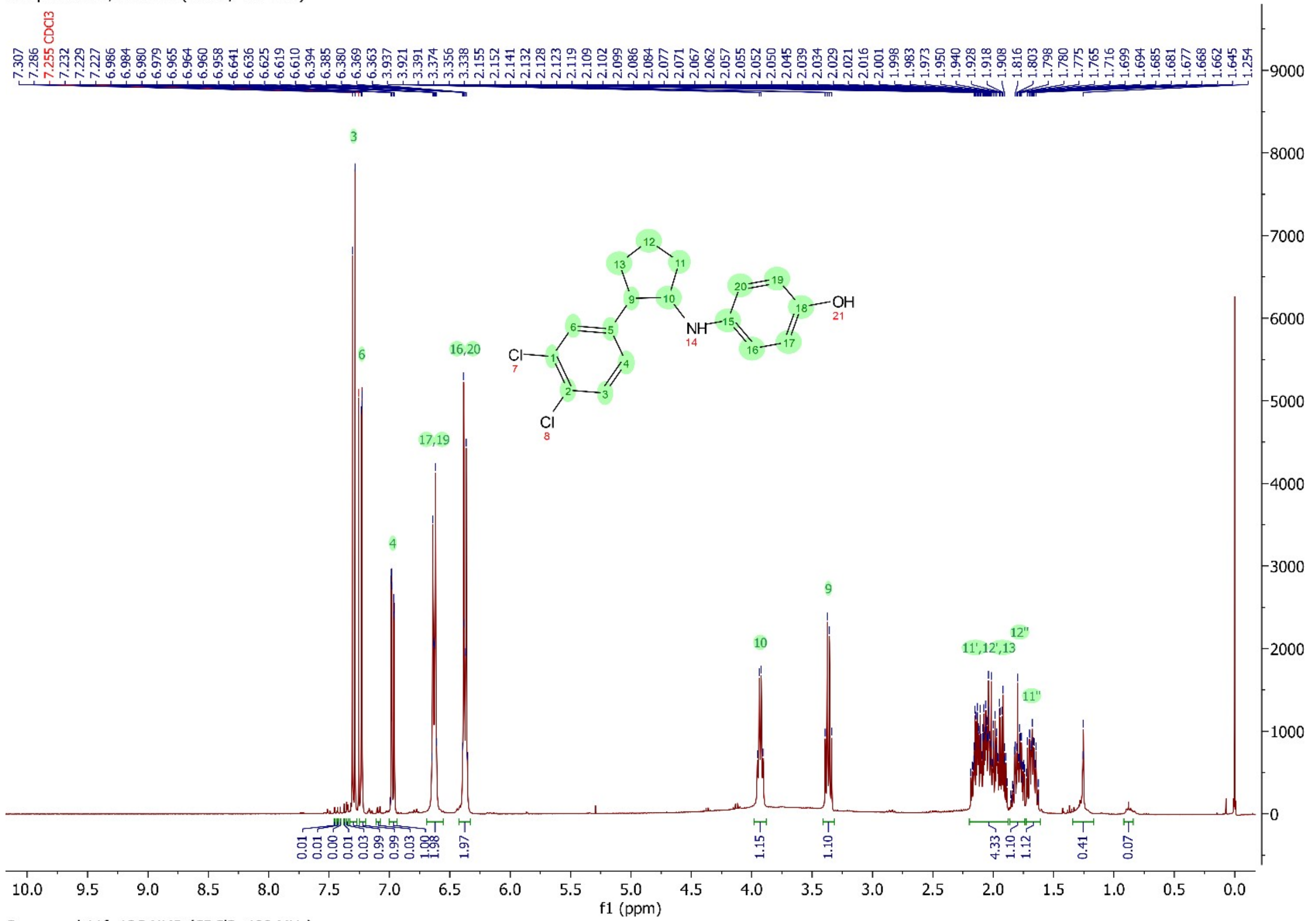

Compound 11f; 13C NMR (CDCl3, 400 MHz)

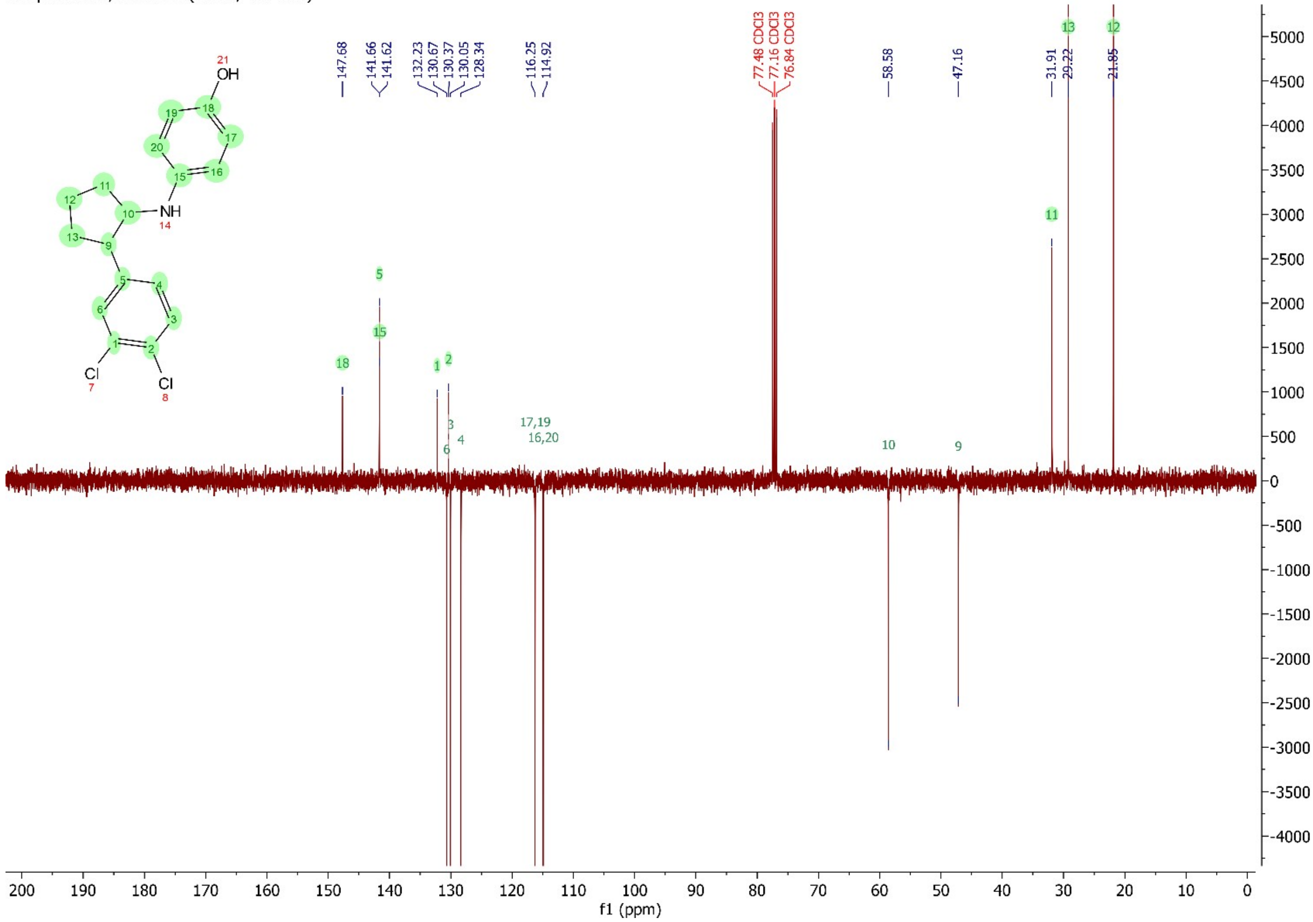

Classified as Internal | Intern

Compound 11g; 1H NMR (CDCl3, 400 MHz)

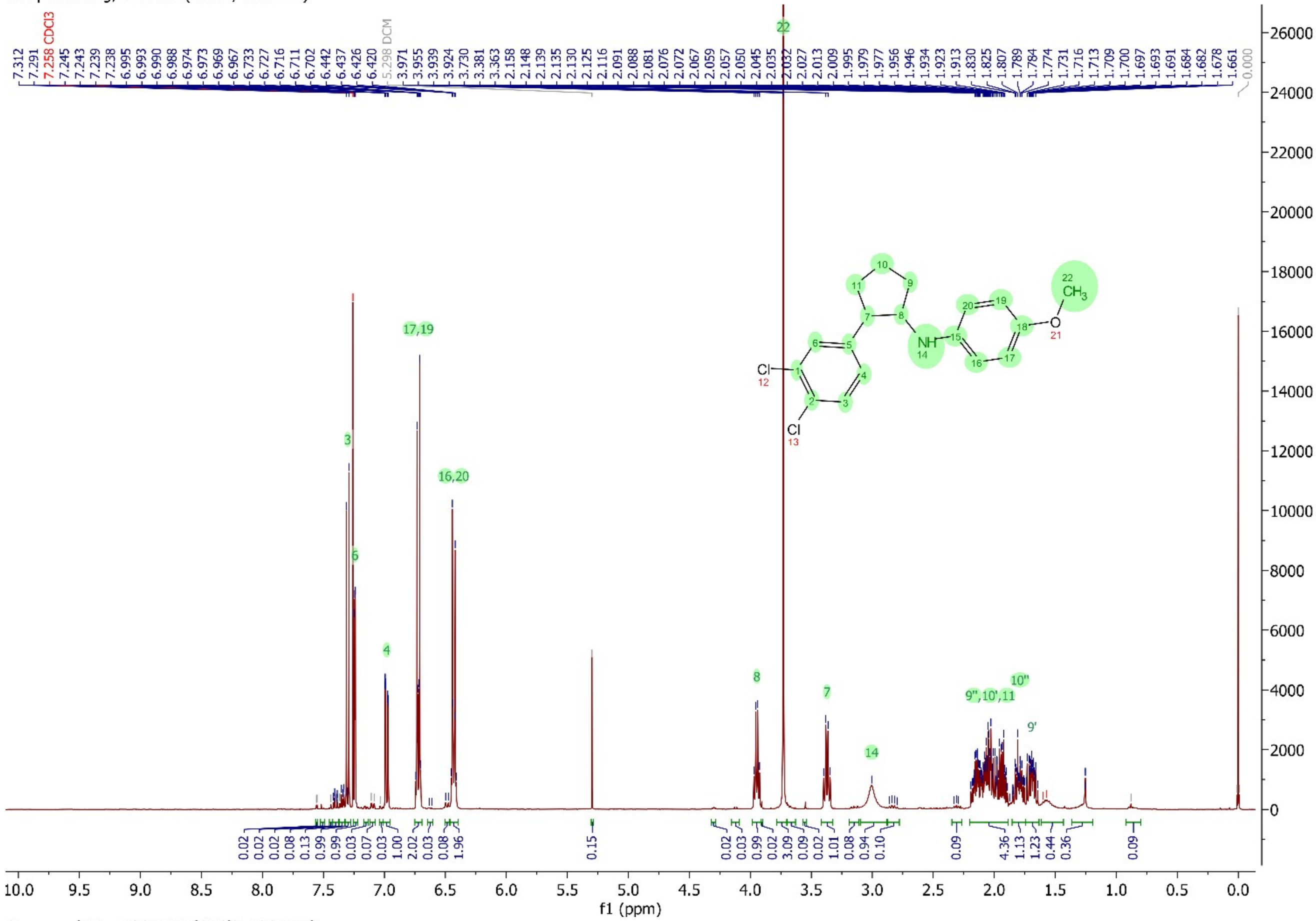

Compound 11g; 13C NMR (CDCl3, 126 MHz)

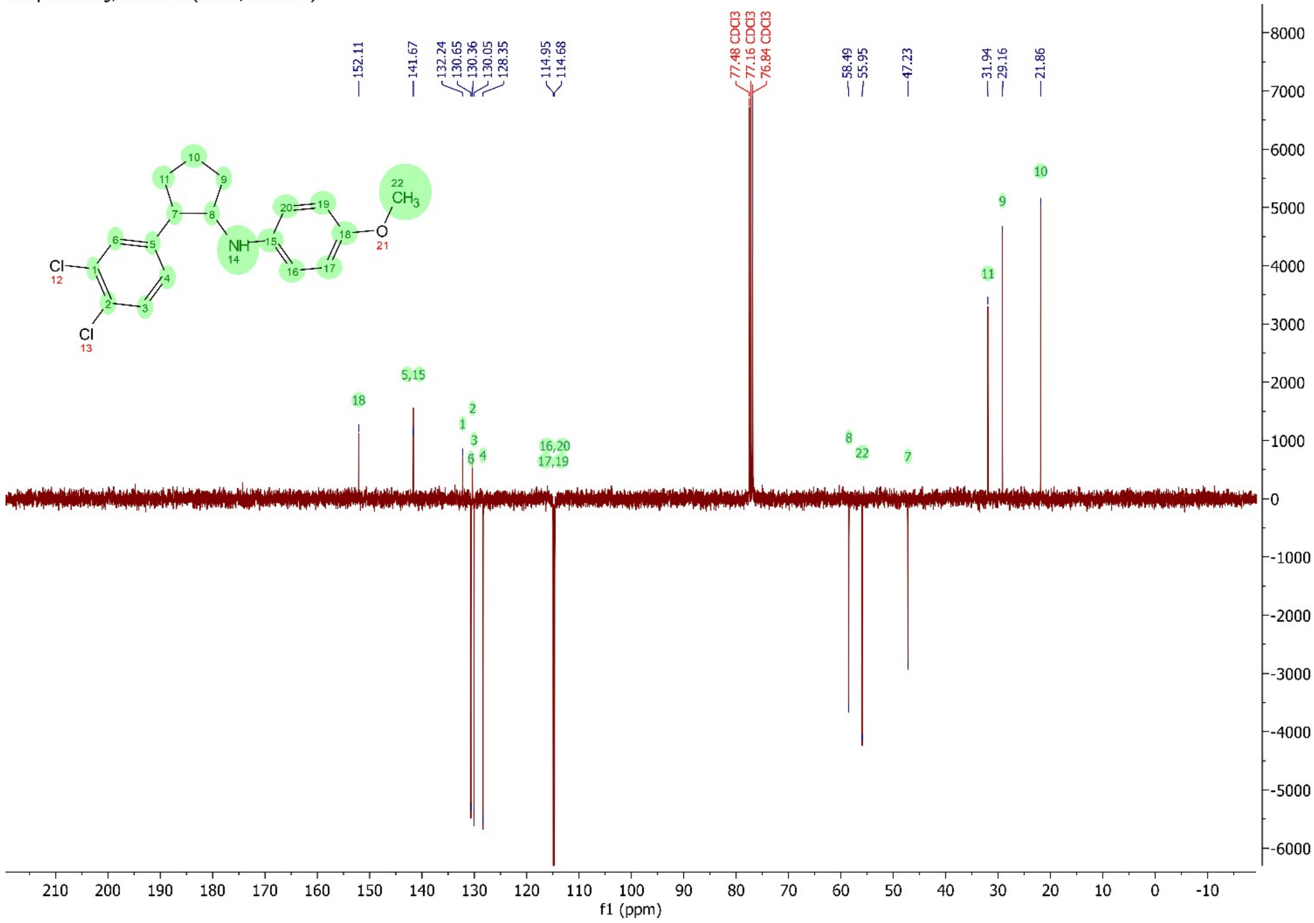

Classified as Internal | Intern

Compound 11h; 1H NMR (CDCl3, 400 MHz)

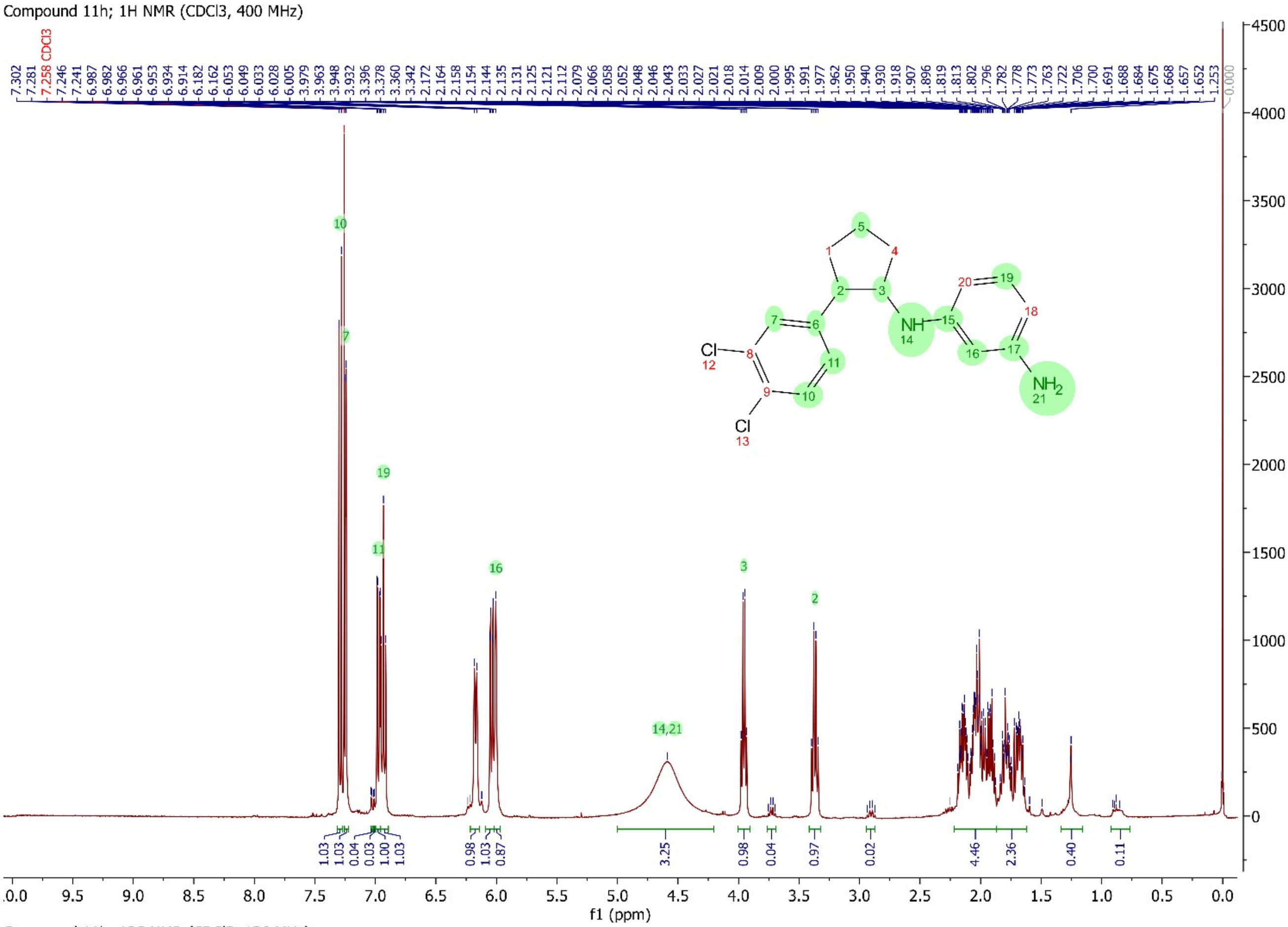

Compound 11h; 13C NMR (CDCl3, 126 MHz)

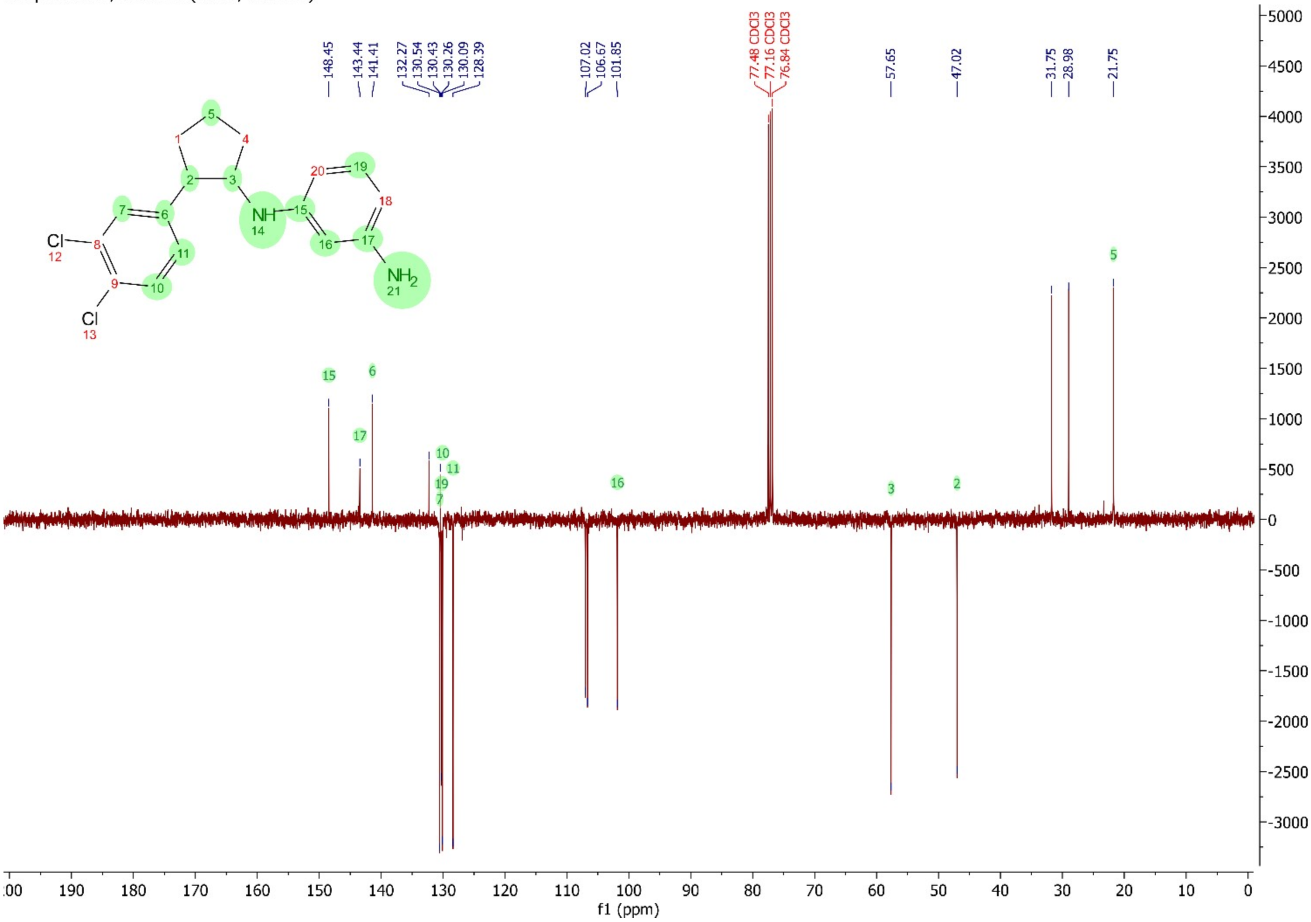

Classified as Internal | Intern

Compound 11i; 1H NMR (CDCl3, 400 MHz)

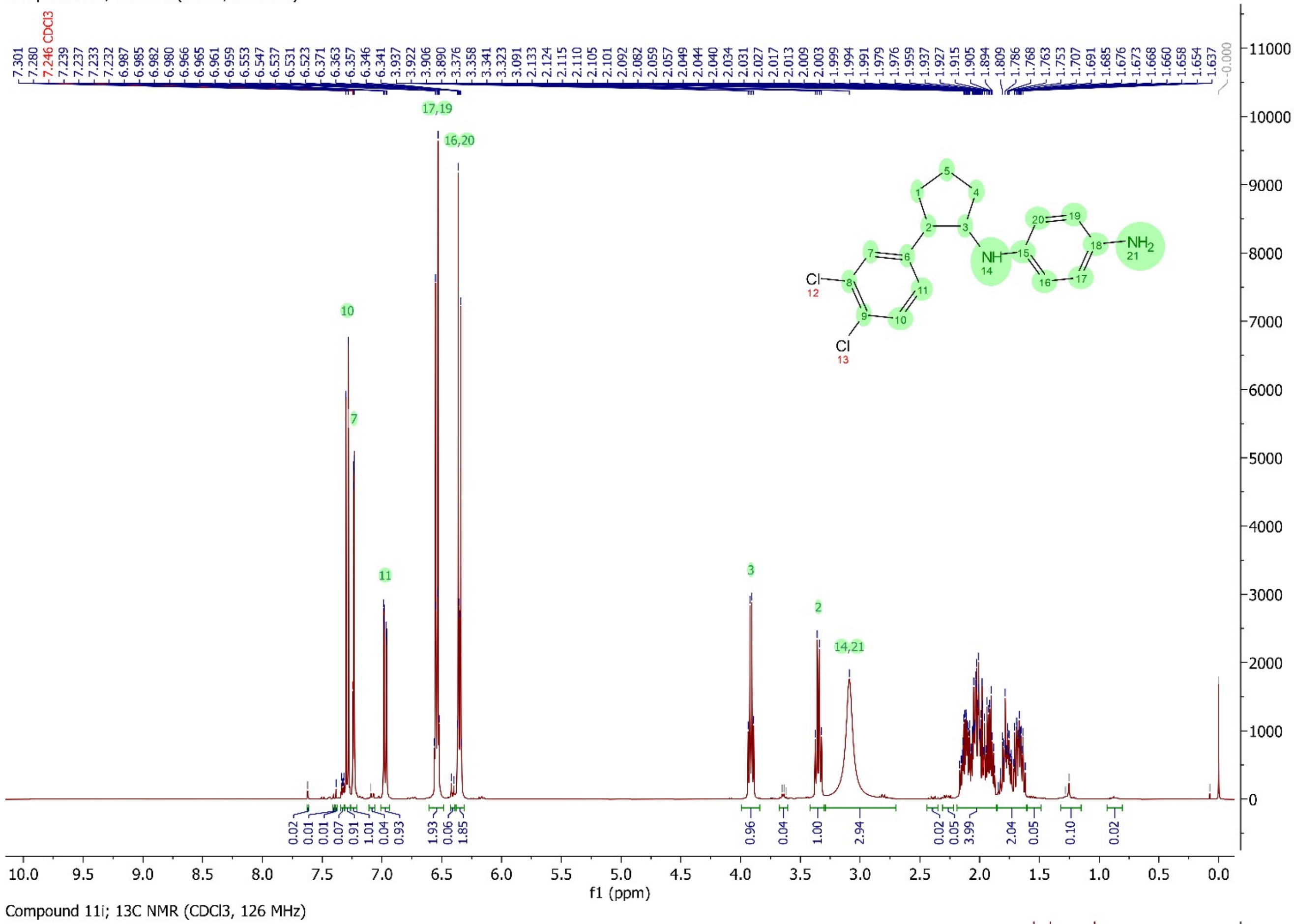

Compound 11i; 13C NMR (CDCl3, 126 MHz)

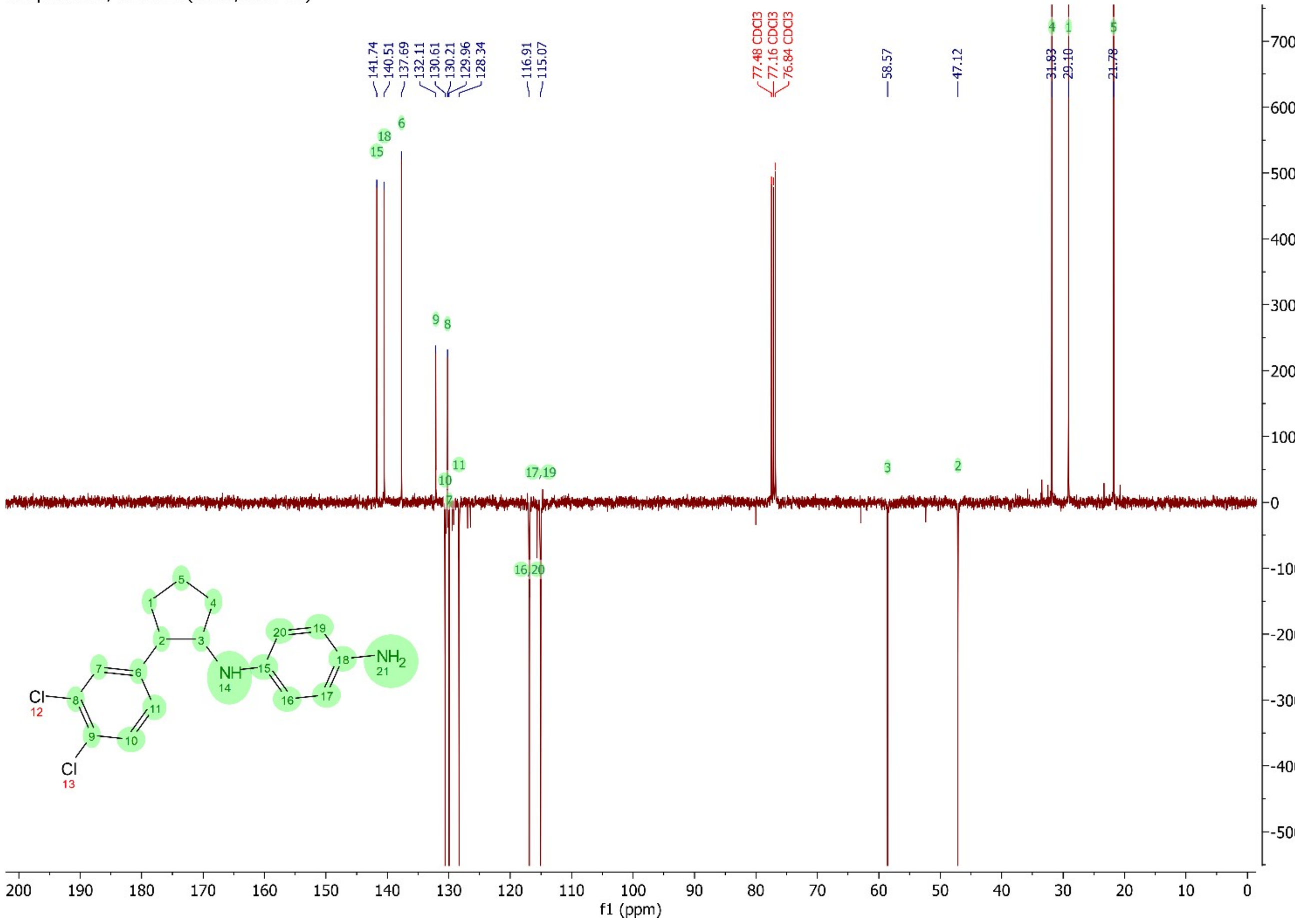

Classified as Internal | Intern

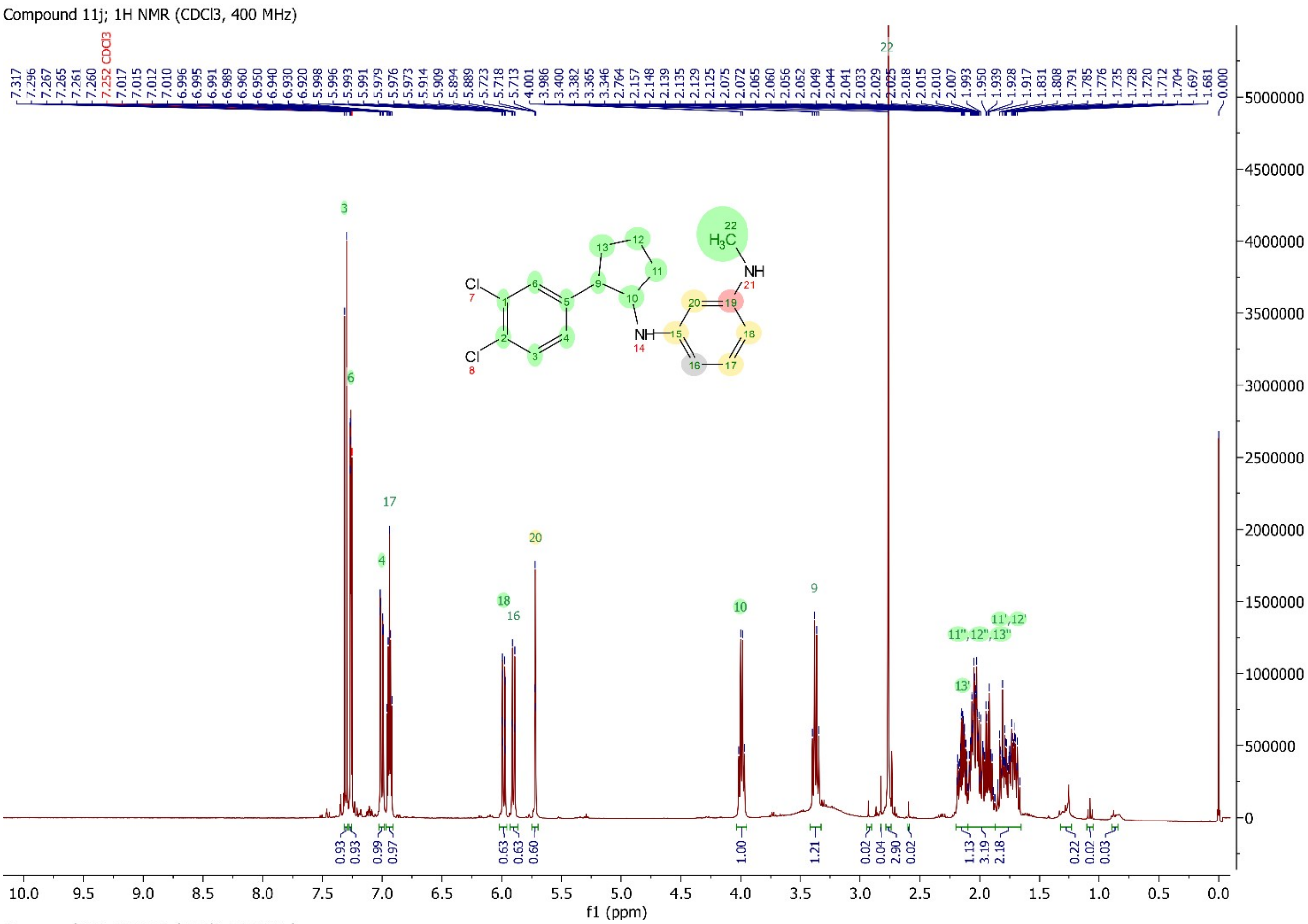

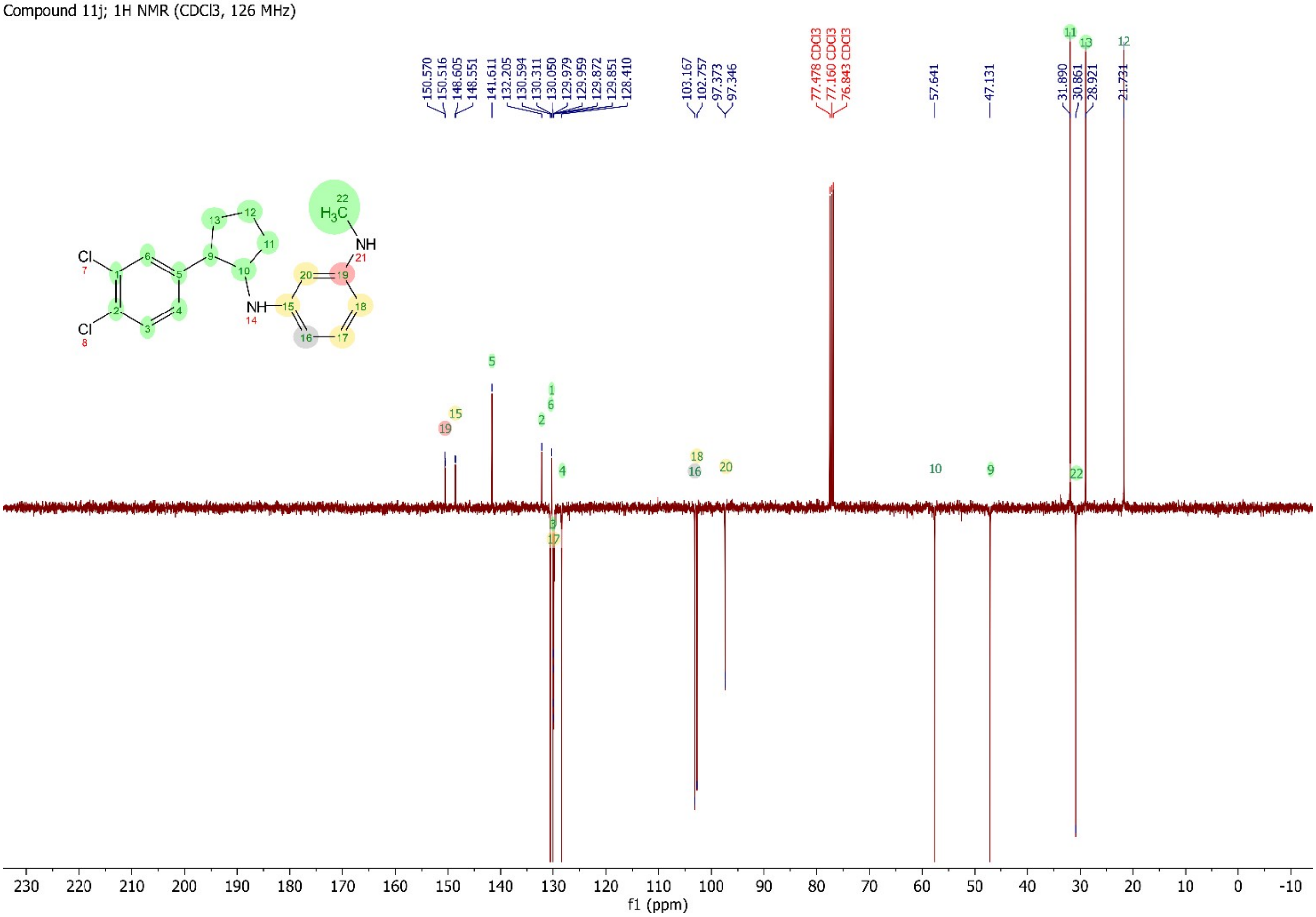

Classified as Internal | Intern

Compound 11k; 1H NMR (CDCl3, 400 MHz)

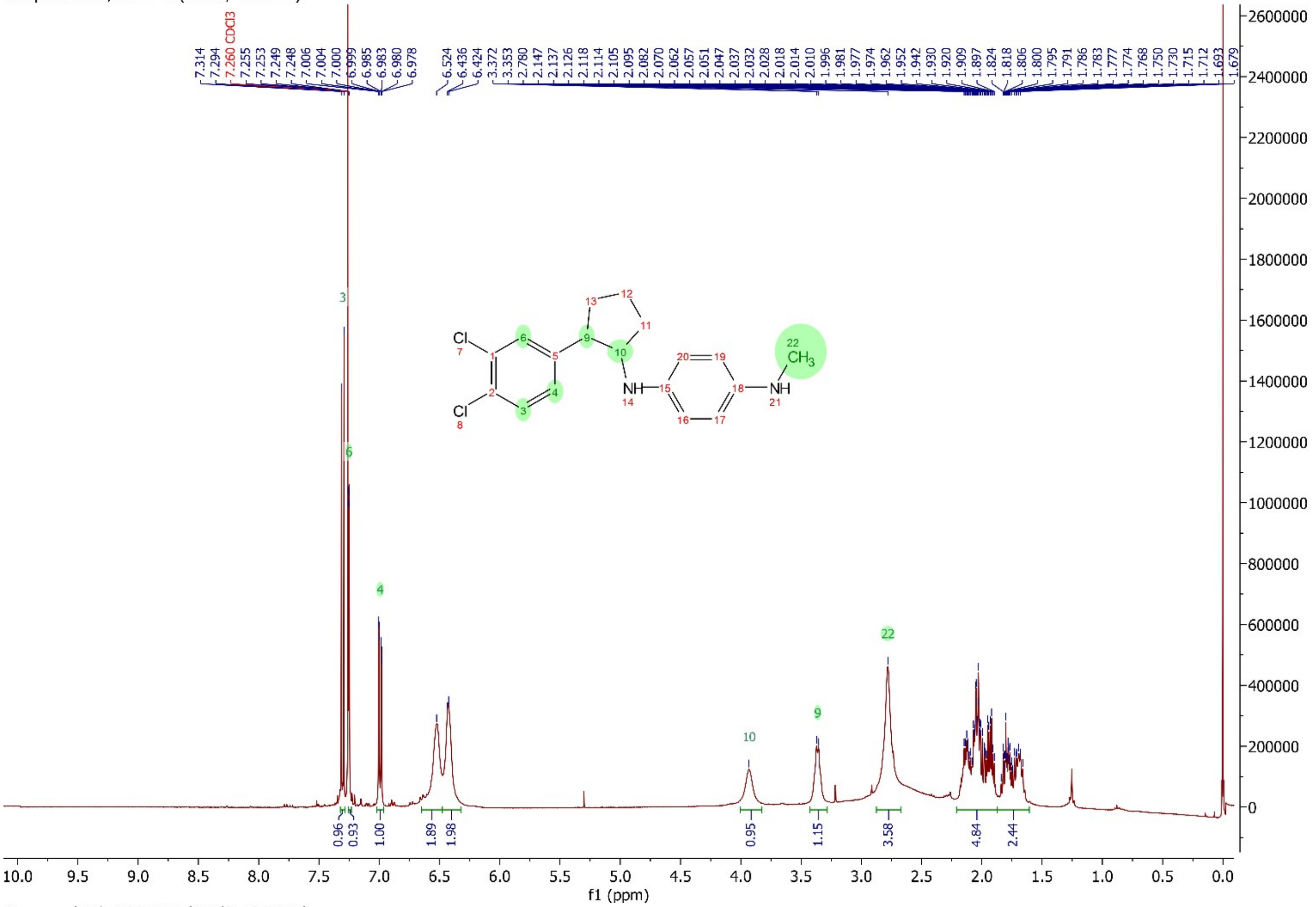

Compound 11k; 13C NMR (CDCl3, 126 MHz)

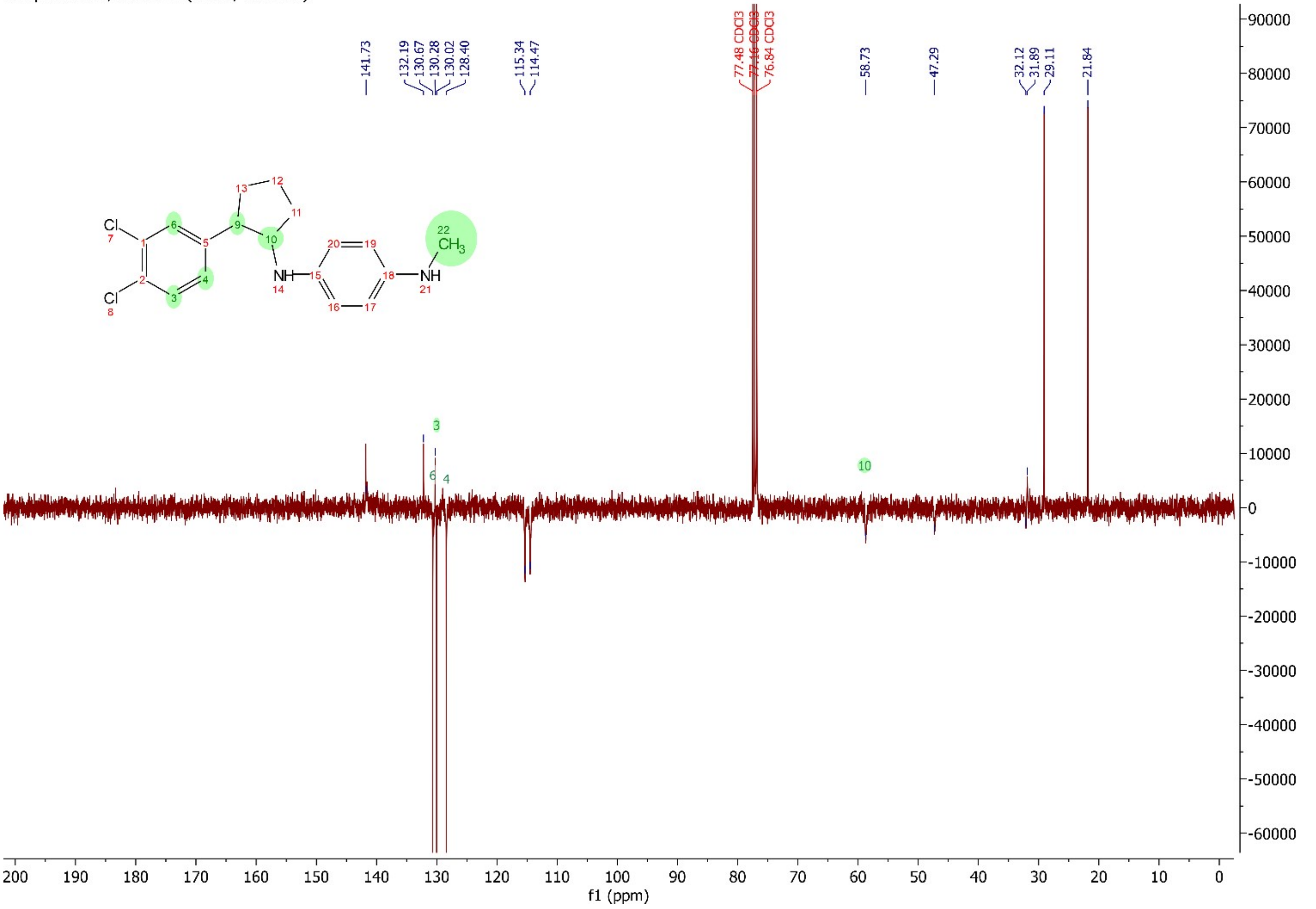

Classified as Internal | Intern

Compound 11l; 1H NMR (CDCl3, 400 MHz)

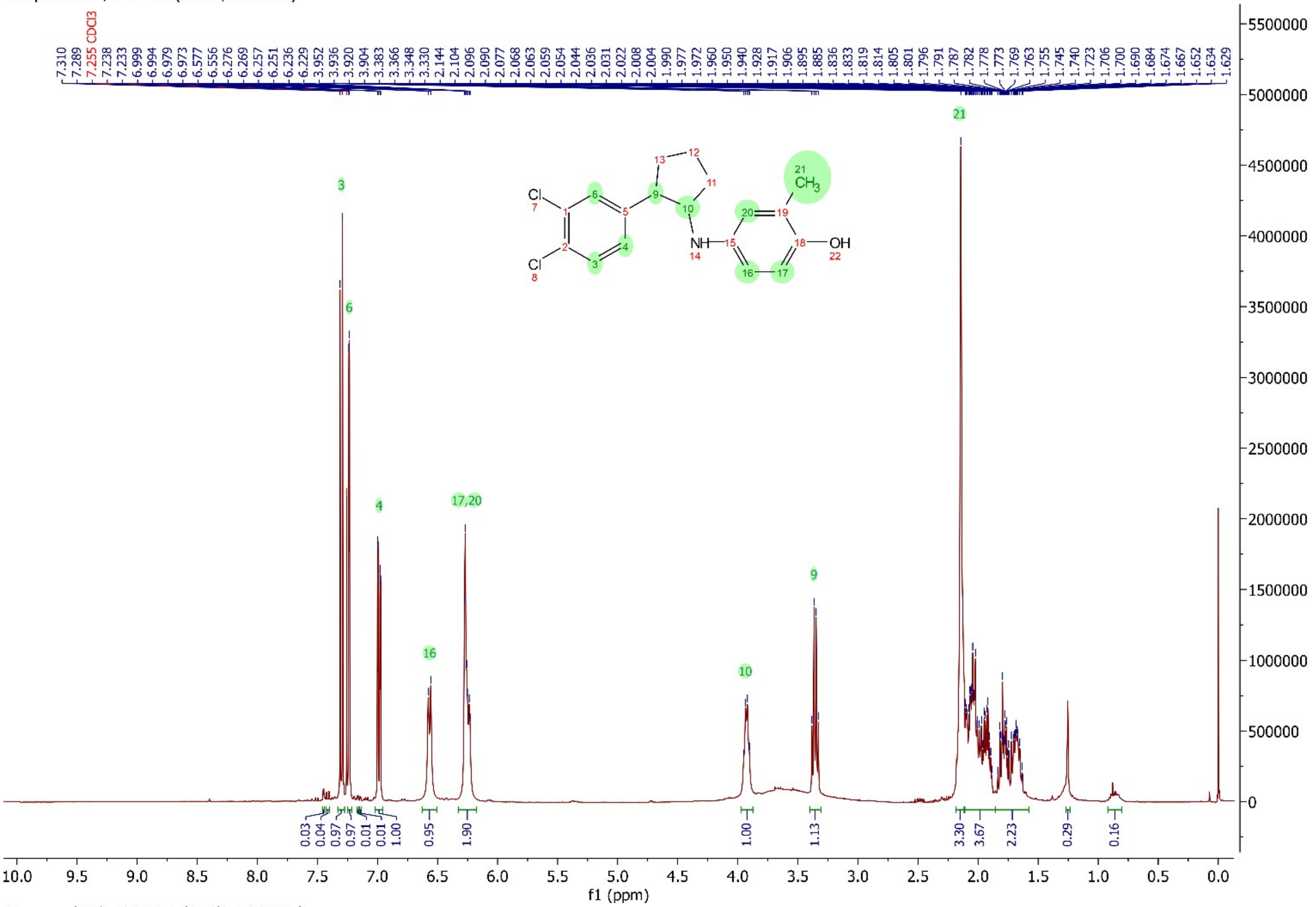

Compound 11l; 13C NMR (CDCl3, 126 MHz)

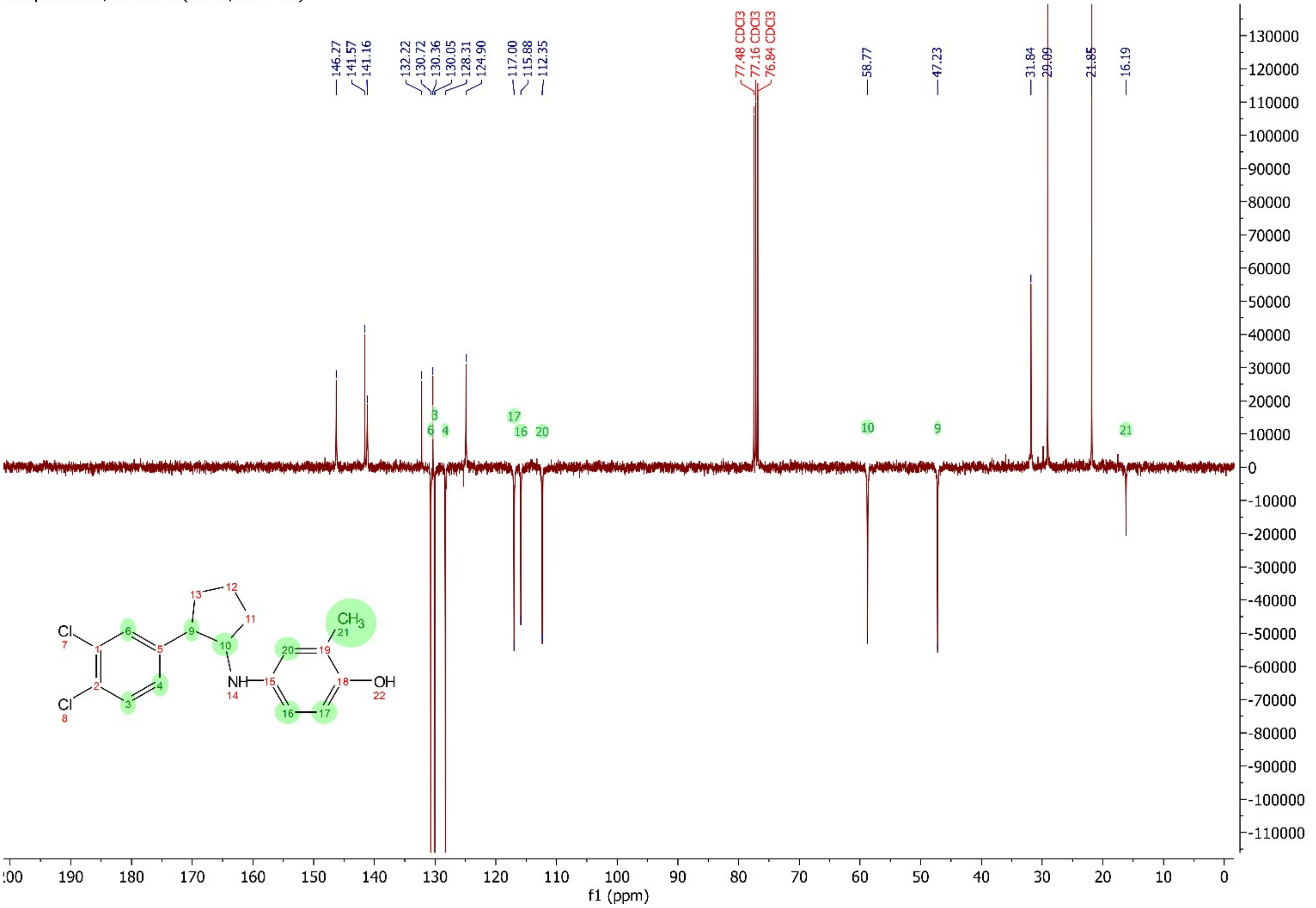

Classified as Internal | Intern

Compound 11m; 1H NMR (CDCl3, 400 MHz)

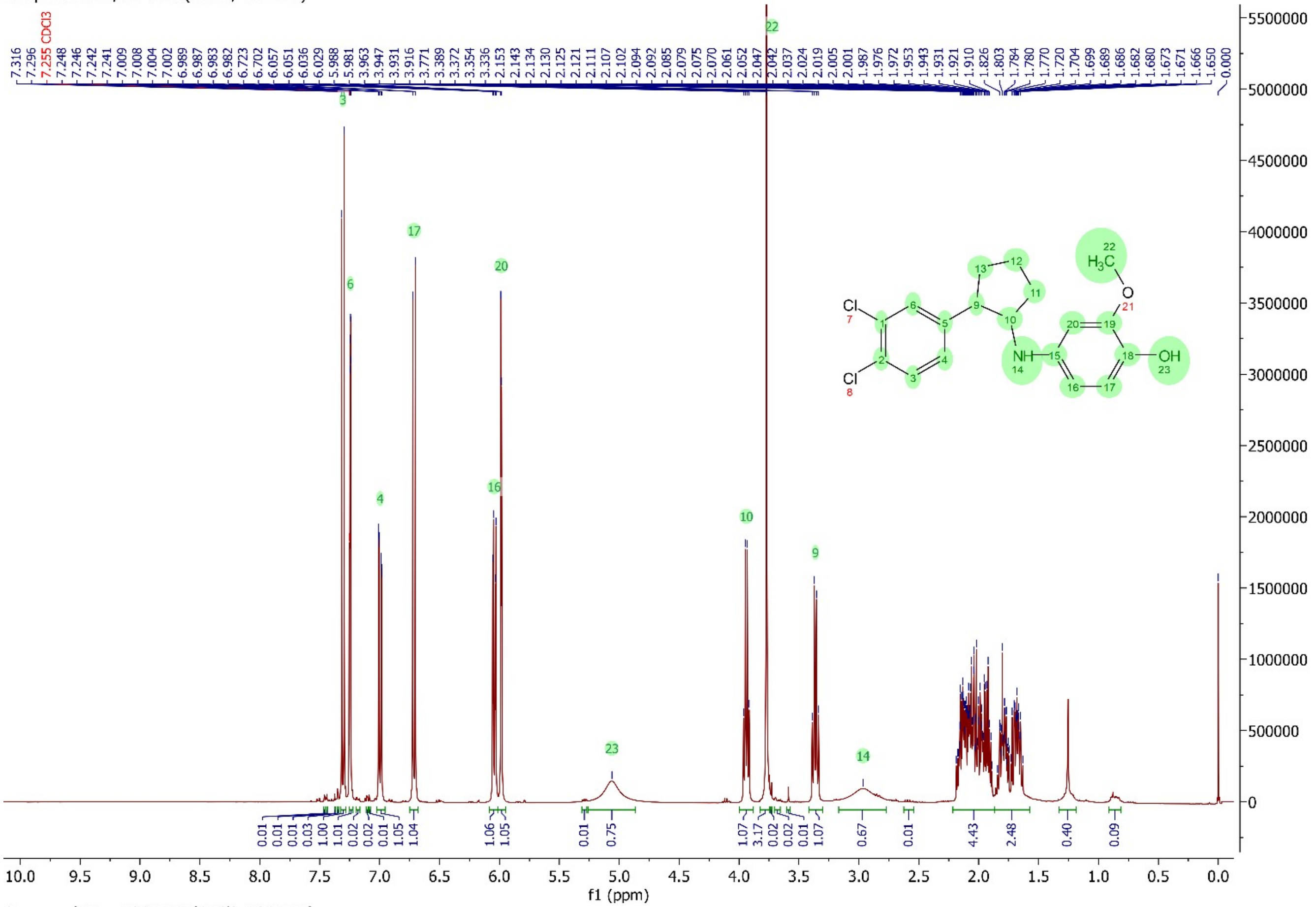

Compound 11m; 13C NMR (CDCl3, 126 MHz)

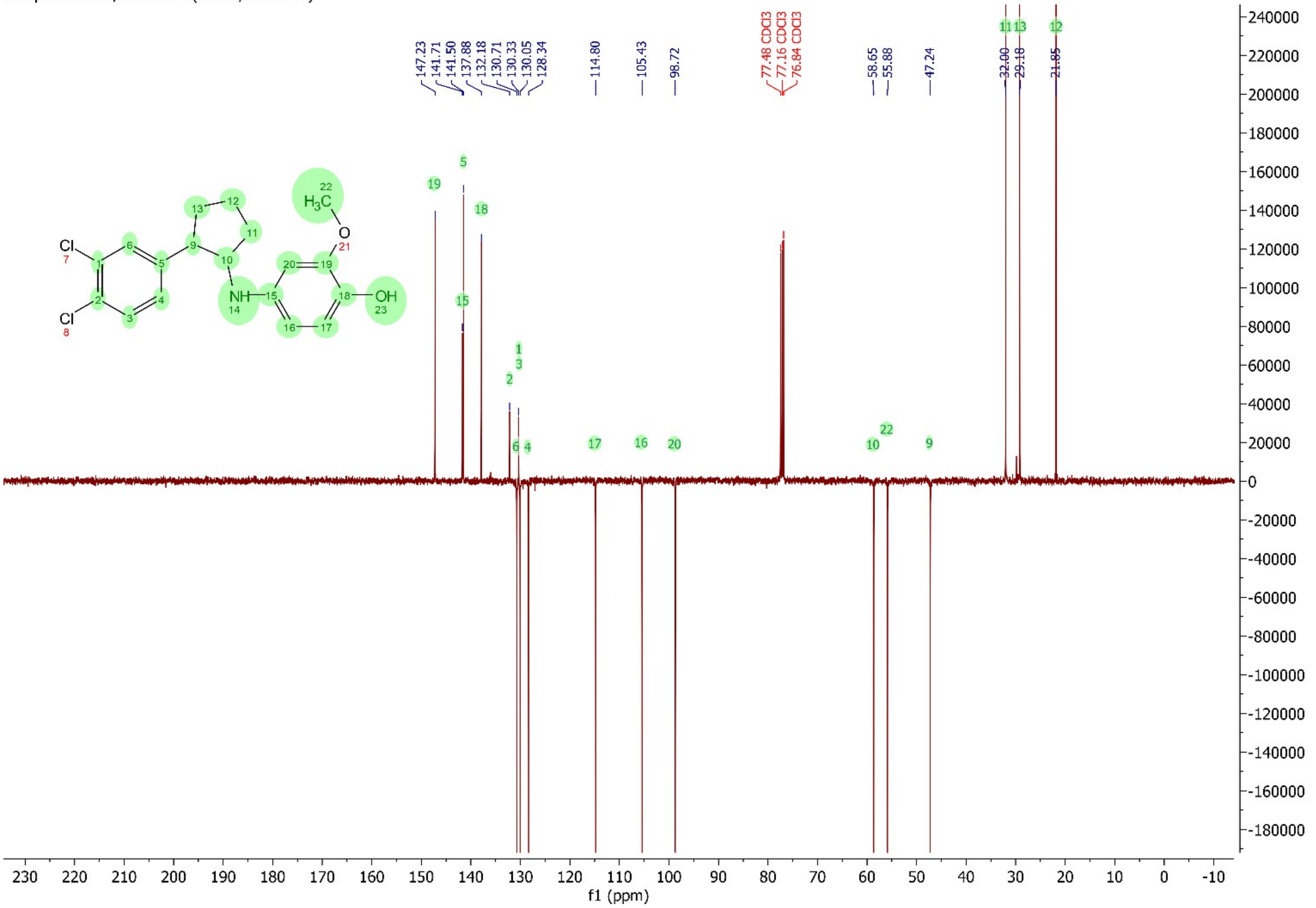

Classified as Internal | Intern

Compound 11n; 1H NMR (MeOD, 400 MHz)

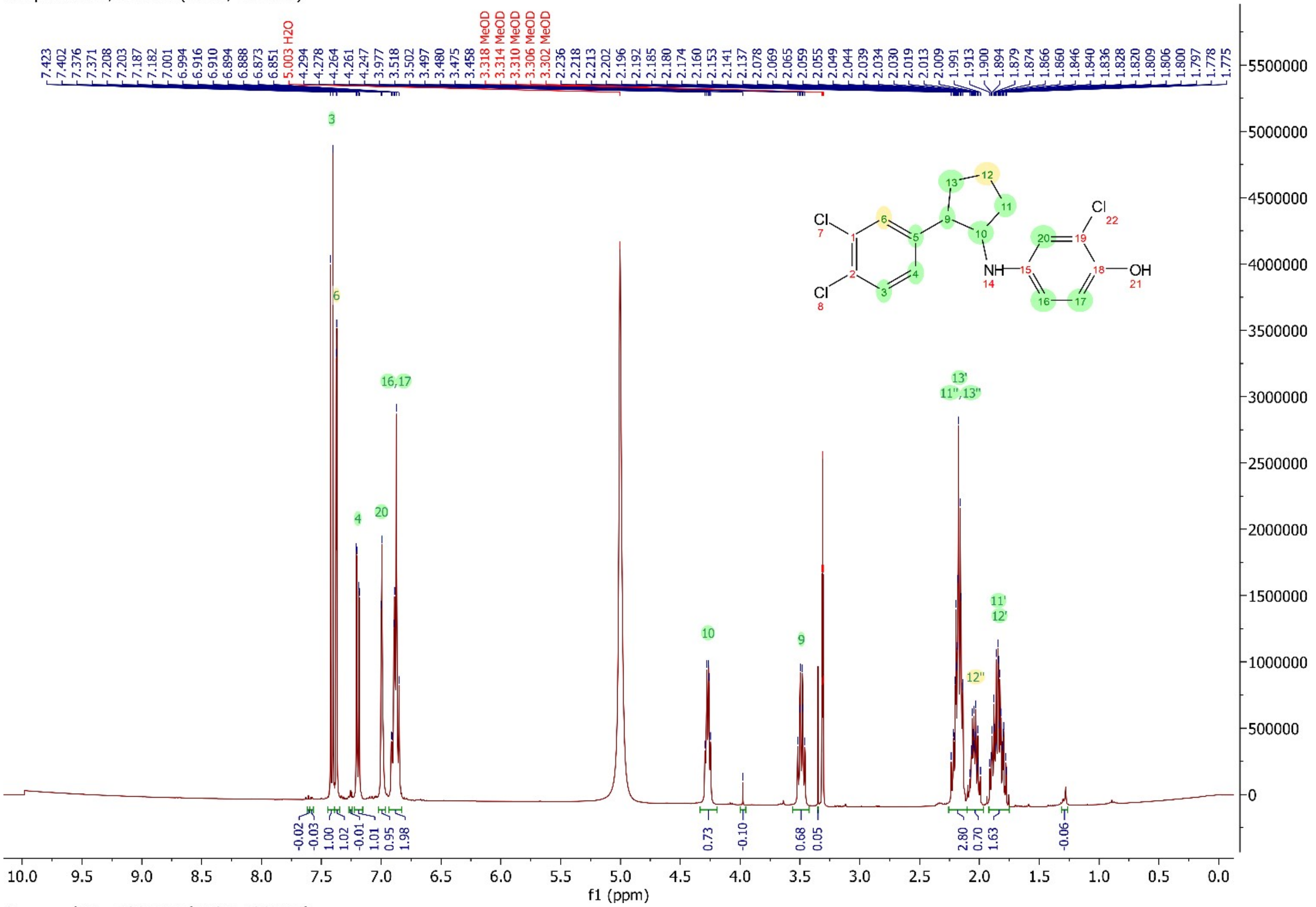

Compound 11n; 13C NMR (MeOD, 126 MHz)

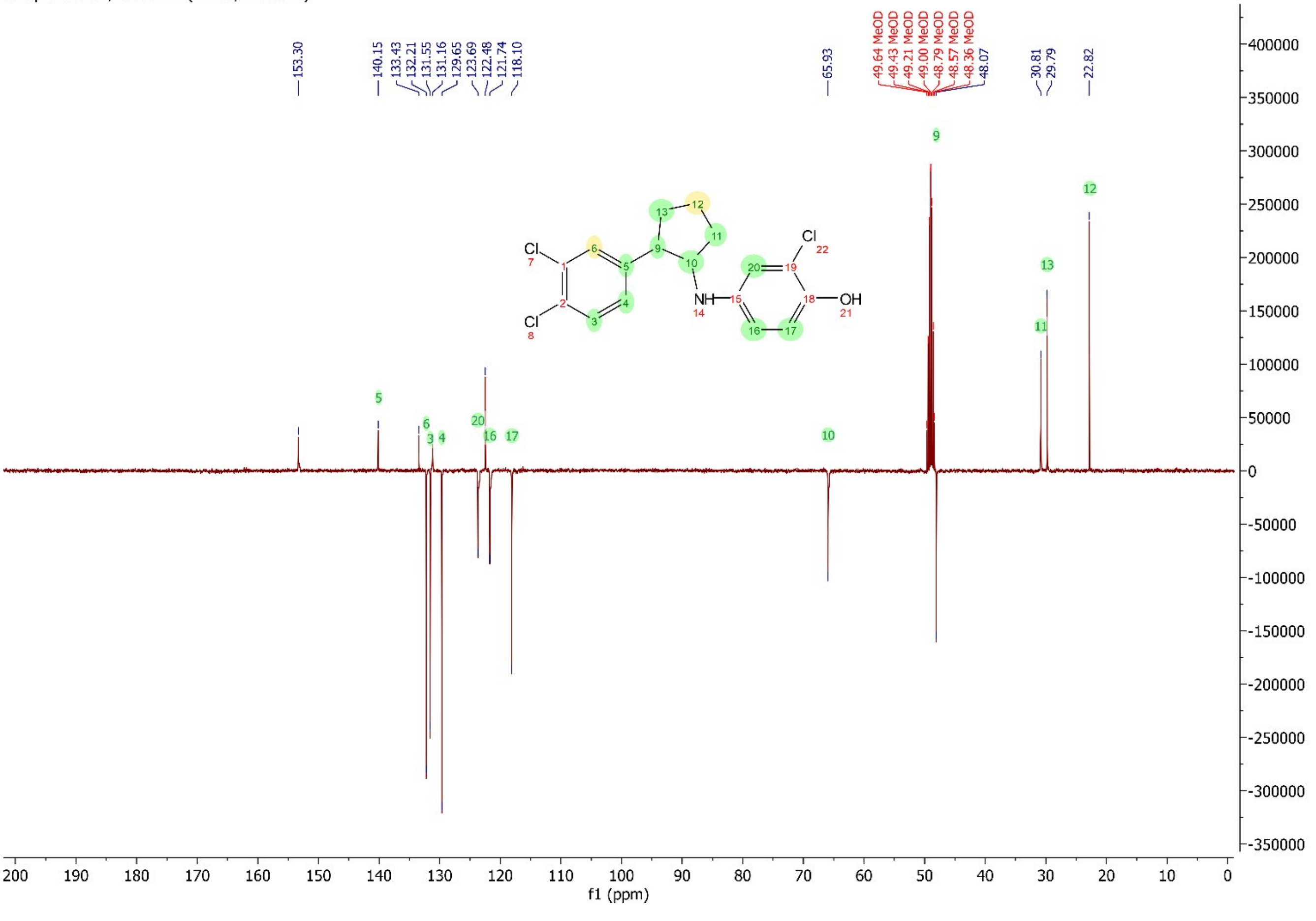

Classified as Internal | Intern

Compound 11n; 1H NMR (MeOD, 400 MHz)

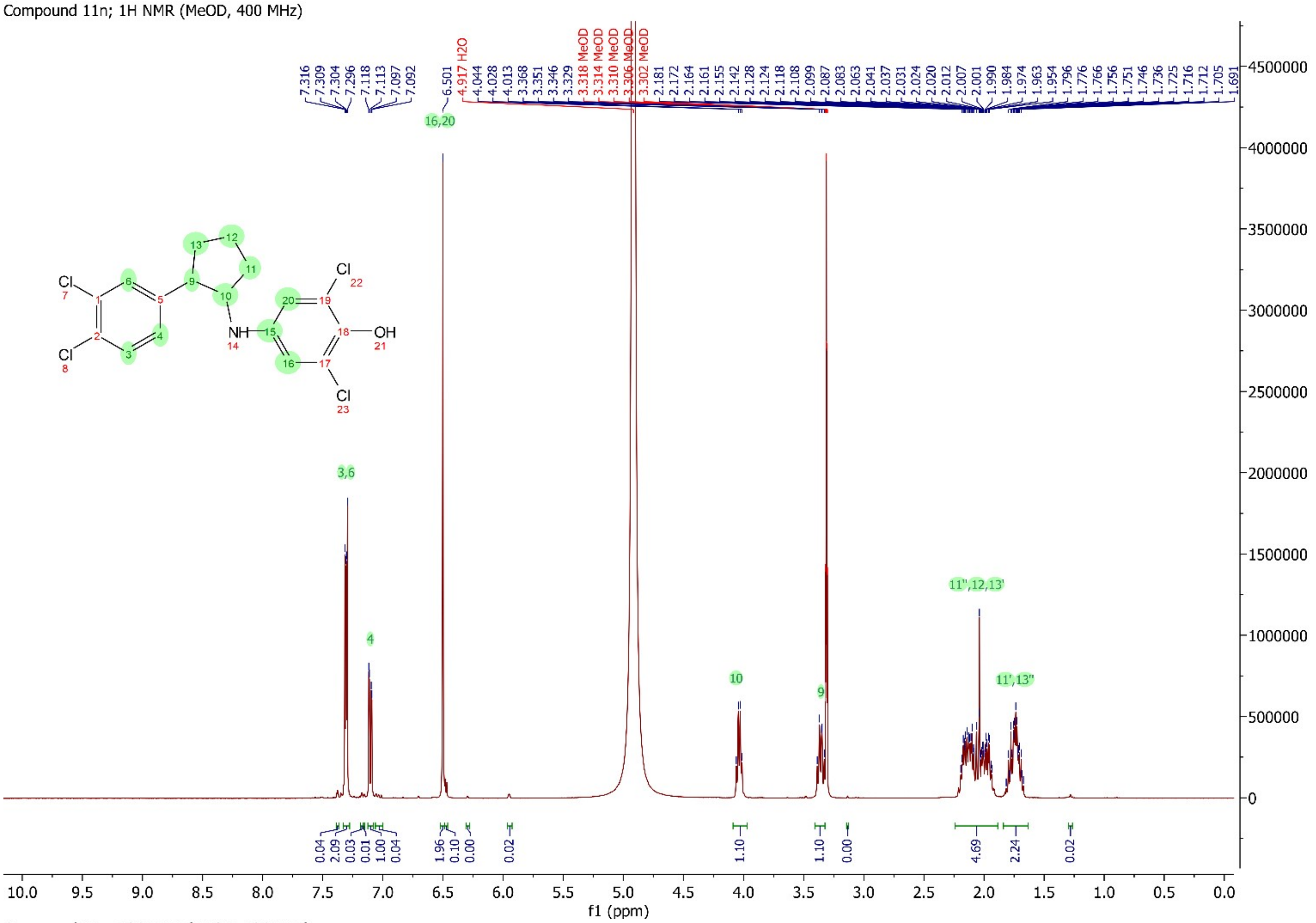

Compound 11o; 13C NMR (MeOD, 126 MHz)

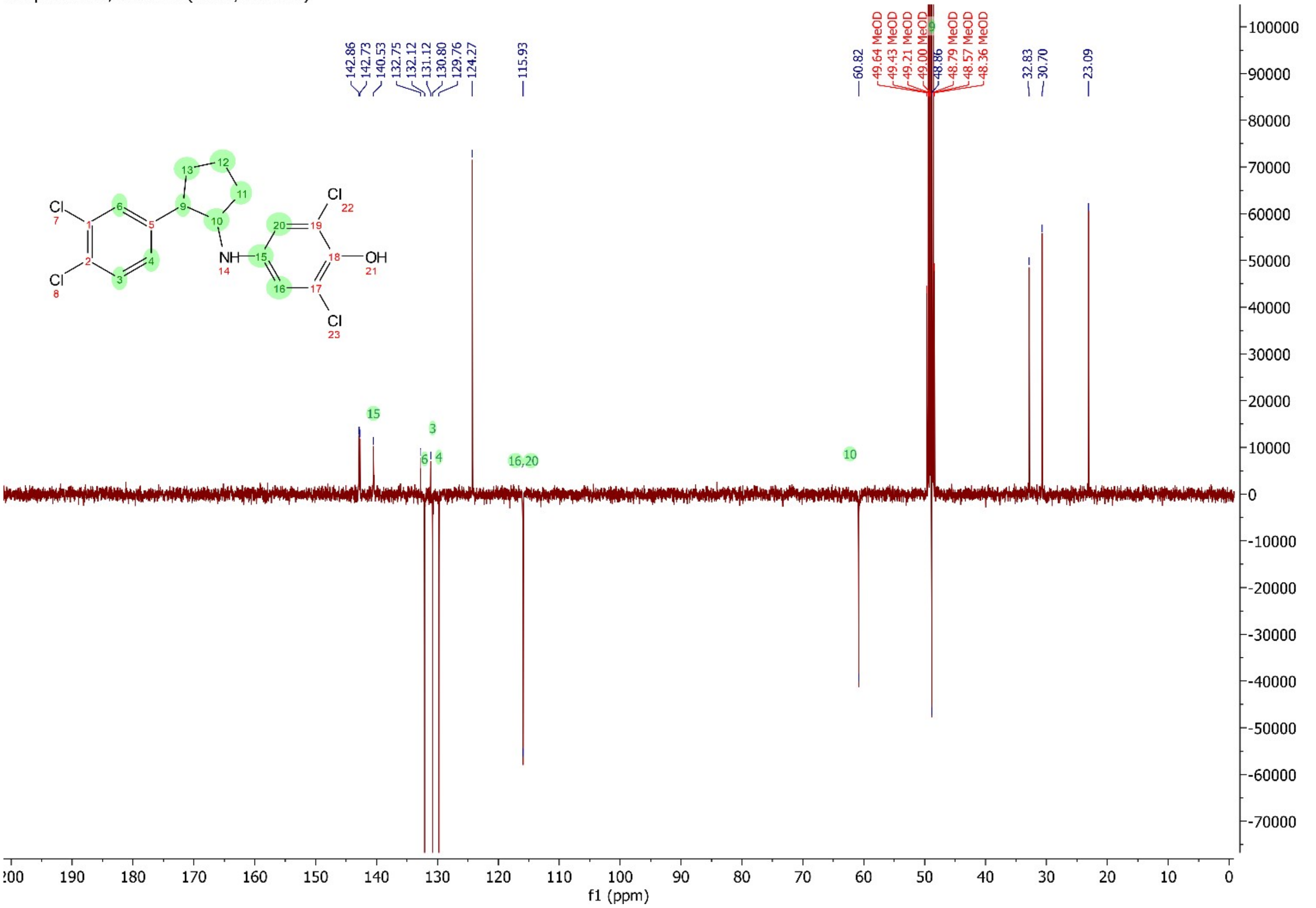

Classified as Internal | Intern

Compound 11p; 1H NMR (MeOD, 400 MHz)

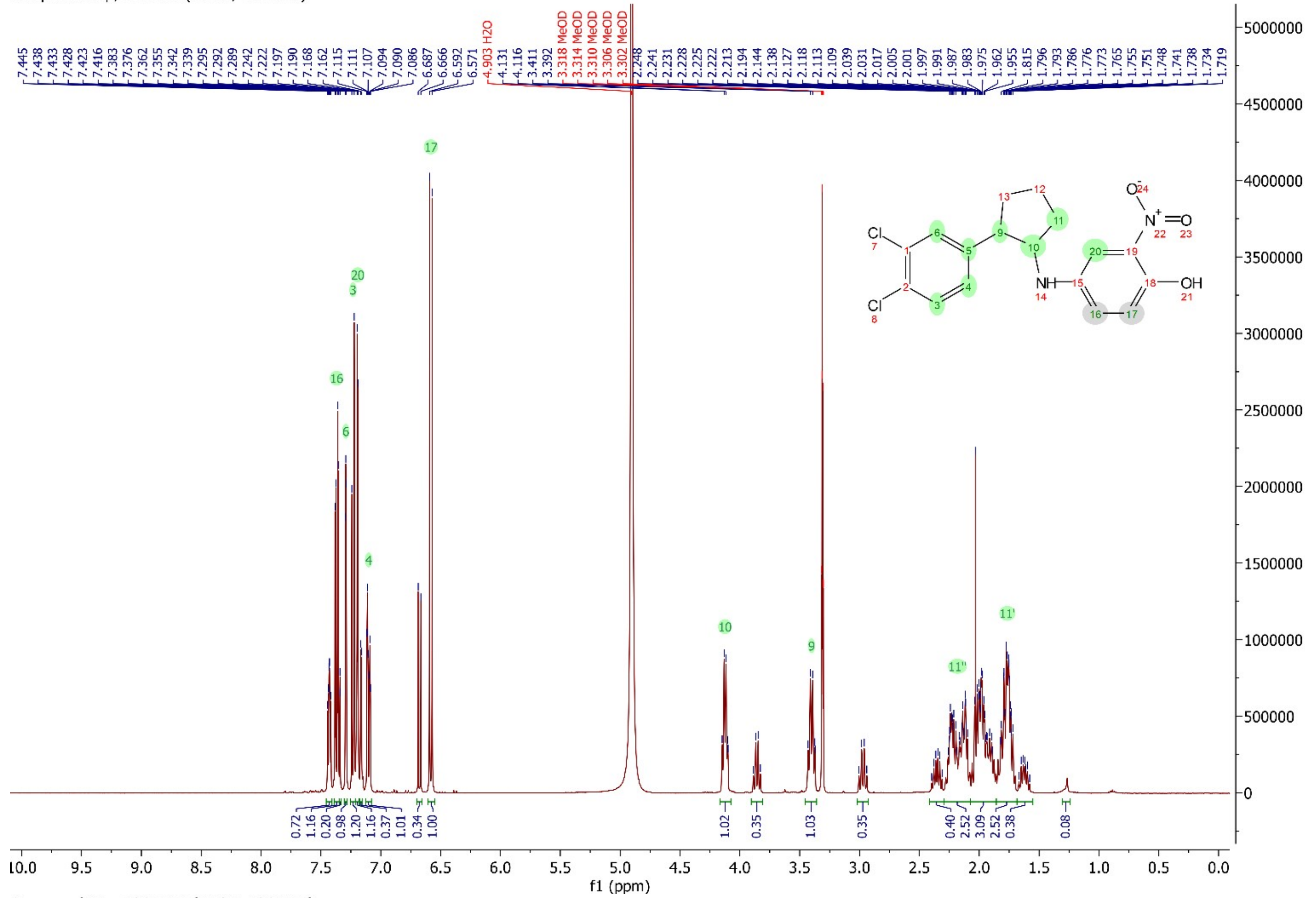

Compound 11p; 13C NMR (MeOD, 126 MHz)

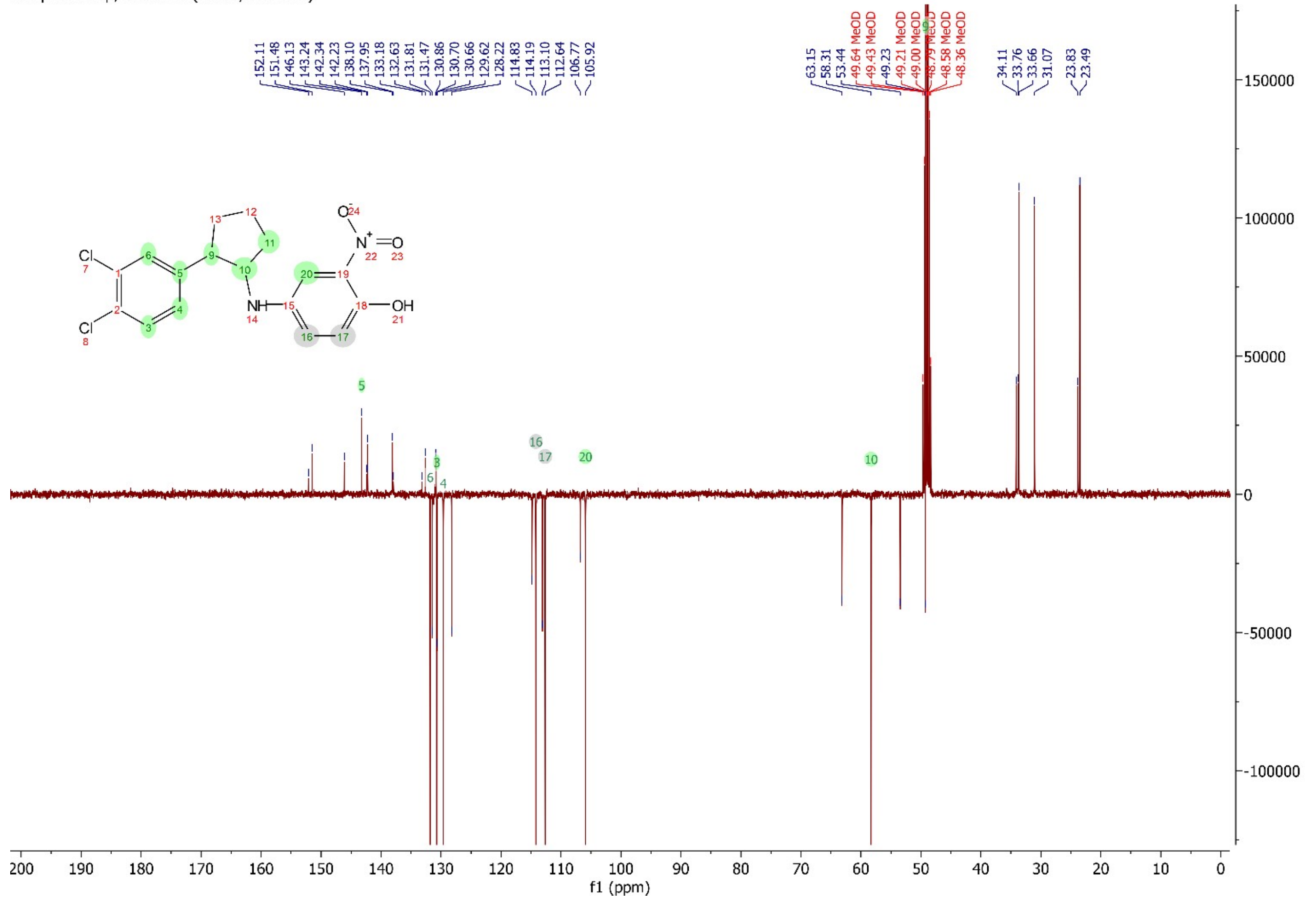

Classified as Internal | Intern

Exemplary NOESY NMR spectra

Compound 11j; NOESY NMR (CDCl3)

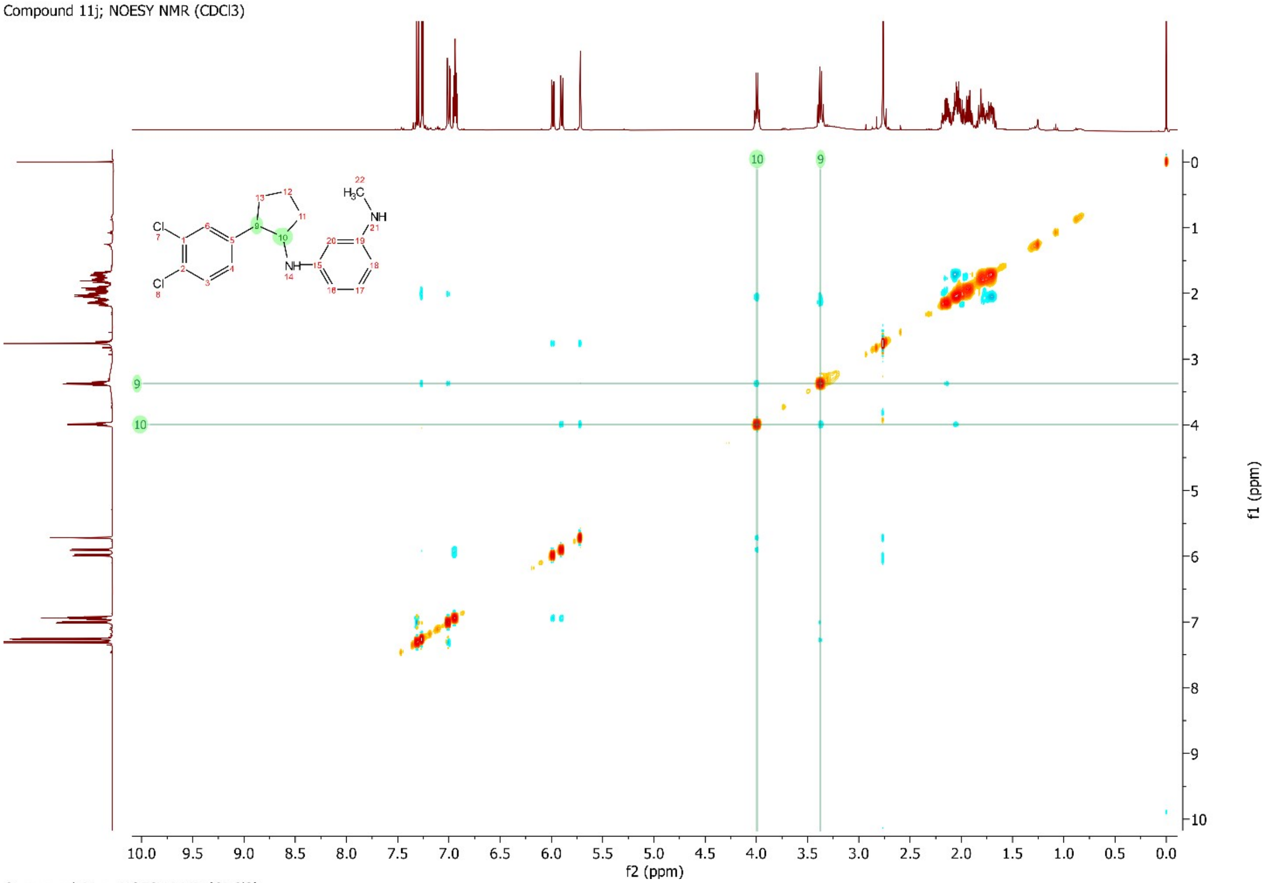

Compound 11m; NOESY NMR (CDCl3)

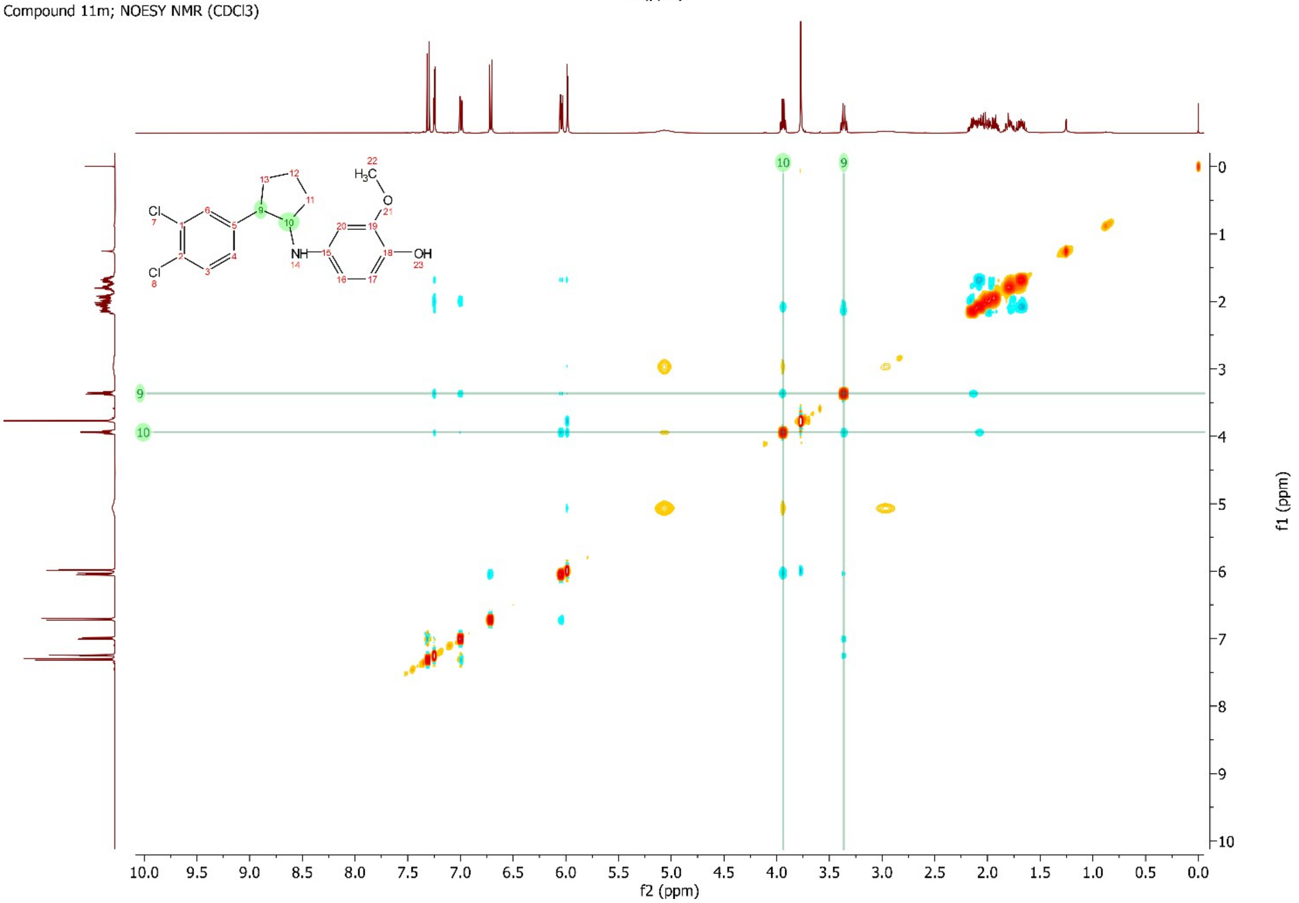

Classified as Internal | Intern

Compound 11n; NOESY NMR (CDCl3)

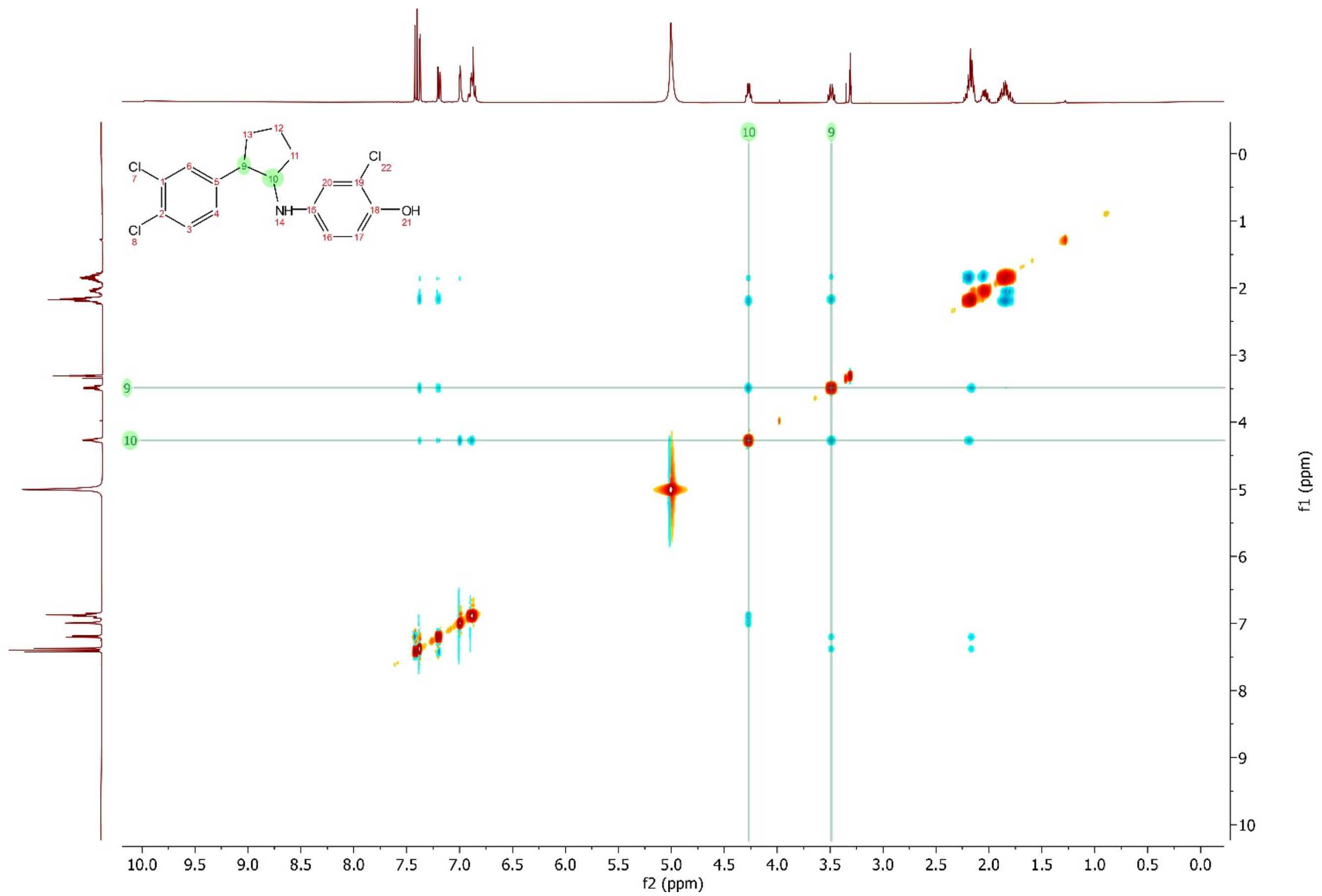

Classified as Internal | Intern

HPLC chromatograms

==== Shimadzu LabSolutions Browser Report ====

Chromatogram(HPLC compound 9a.lcd)

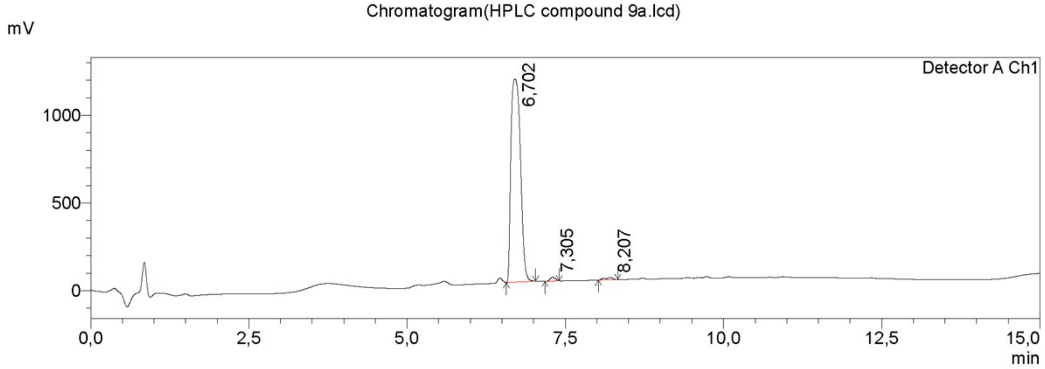

Peak Table(HPLC compound 9a.lcd)

Detector A 215nm

Peak#	Ret. Time	Area	Height	Mark	Conc.	Area%
1	6,702	12149558	1159356	M	97,929	97,929
2	7,305	137470	21915	M	1,108	1,108
3	8,207	119502	13714	M	0,963	0,963
Total		12406529	1194985		100,000	100,000

Chromatogram(HPLC compound 10b.lcd)

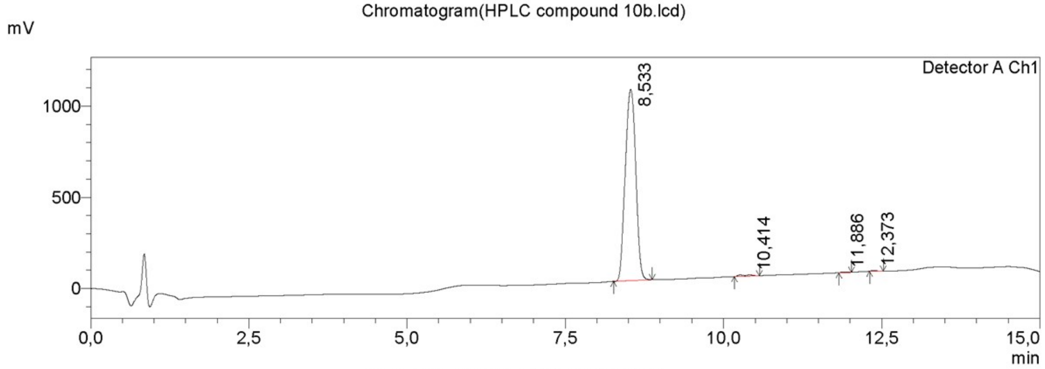

Peak Table(HPLC compound 10b.lcd)

Detector A 215nm

Peak#	Ret. Time	Area	Height	Mark	Conc.	Area%
1	8,533	11995424	1047669	M	98,911	98,911
2	10,414	95556	6463	M	0,788	0,788
3	11,886	11687	2126	M	0,096	0,096
4	12,373	24830	4398	M	0,205	0,205
Total		12127497	1060655		100,000	100,000

Classified as Internal | Intern

==== Shimadzu LabSolutions Browser Report ====

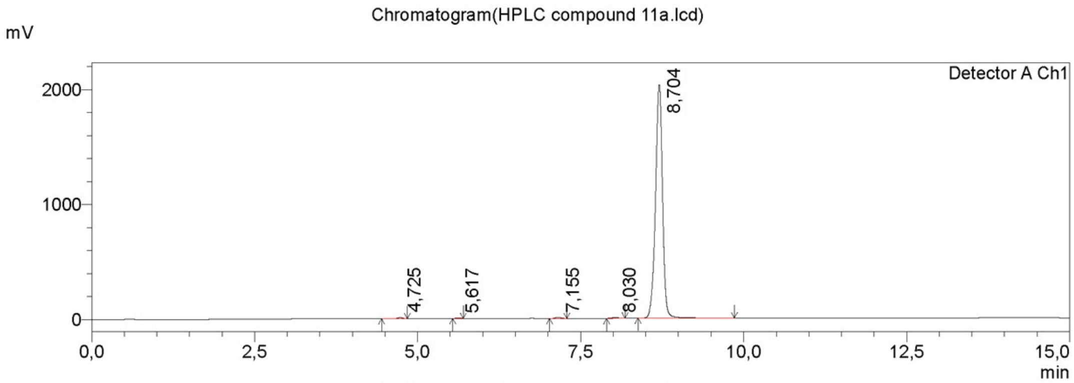

Peak Table(HPLC compound 11a.lcd)

Detector A 254nm

Peak#	Ret. Time	Area	Height	Mark	Conc.	Area%
1	4,725	57155	10544	M	0,370	0,370
2	5,617	20537	4115	M	0,133	0,133
3	7,155	62027	10279	M	0,401	0,401
4	8,030	50805	7674	M	0,329	0,329
5	8,704	15270283	2028164	M	98,768	98,768
Total		15460806	2060776		100,000	100,000

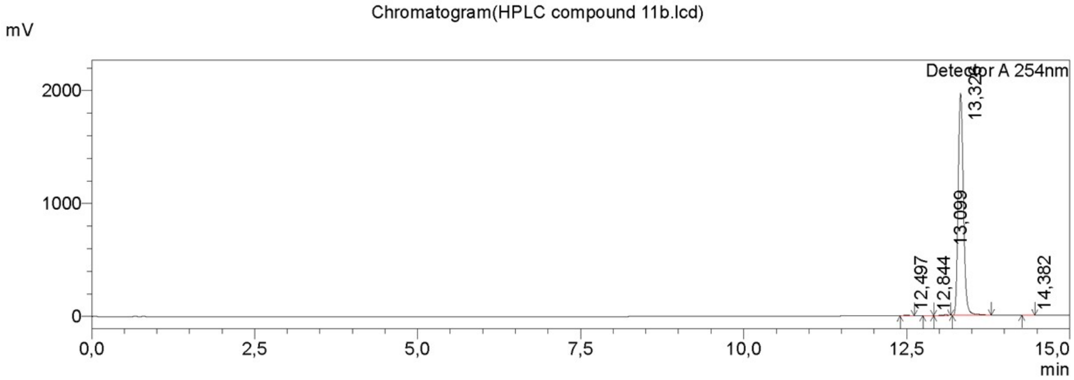

Peak Table(HPLC compound 11b.lcd)

Detector A 254nm

Peak#	Ret. Time	Area	Height	Mark	Conc.	Area%
1	12,497	26145	4725	M	0,233	0,233
2	12,844	3868	769	M	0,035	0,035
3	13,099	39854	6918	M	0,355	0,355
4	13,326	11134790	1967055	M	99,311	99,311
5	14,382	7358	1444	M	0,066	0,066
Total		11212015	1980912		100,000	100,000

Classified as Internal | Intern

==== Shimadzu LabSolutions Browser Report ====

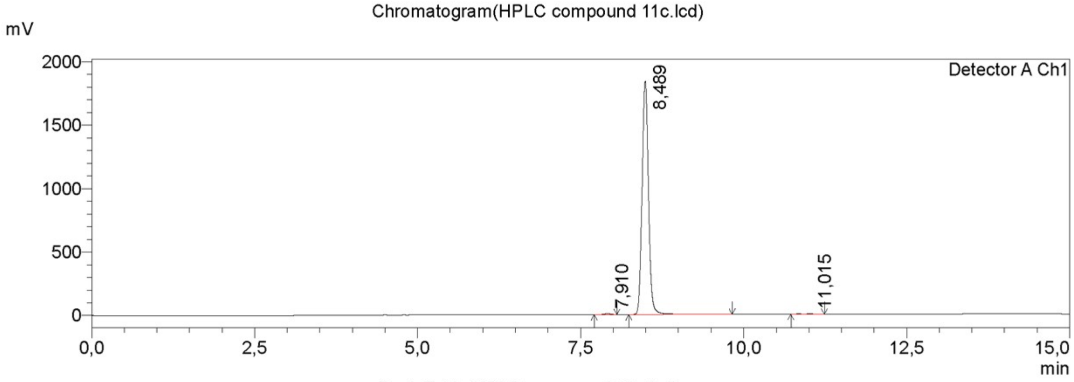

Peak Table(HPLC compound 11c.lcd)

Detector A 254nm

Peak#	Ret. Time	Area	Height	Mark	Conc.	Area%
1	7,910	48791	7188	M	0,390	0,390
2	8,489	12418729	1838237	M	99,156	99,156
3	11,015	56853	4448	M	0,454	0,454
Total		12524372	1849872		100,000	100,000

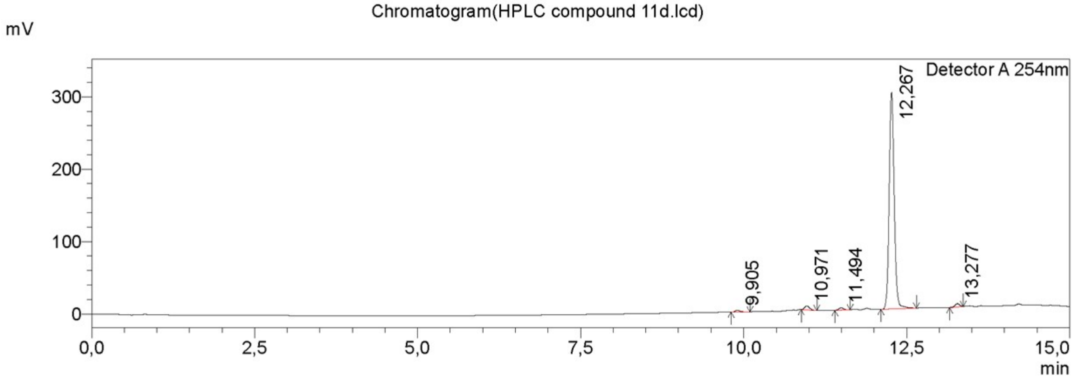

Peak Table(HPLC compound 11d.lcd)

Detector A 254nm

Peak#	Ret. Time	Area	Height	Mark	Conc.	Area%
1	9,905	11270	2248	M	0,673	0,673
2	10,971	27790	5415	M	1,660	1,660
3	11,494	16803	3362	M	1,004	1,004
4	12,267	1595769	299274	M	95,334	95,334
5	13,277	22237	4510	M	1,328	1,328
Total		1673870	314808		100,000	100,000

Classified as Internal | Intern

==== Shimadzu LabSolutions Browser Report ====

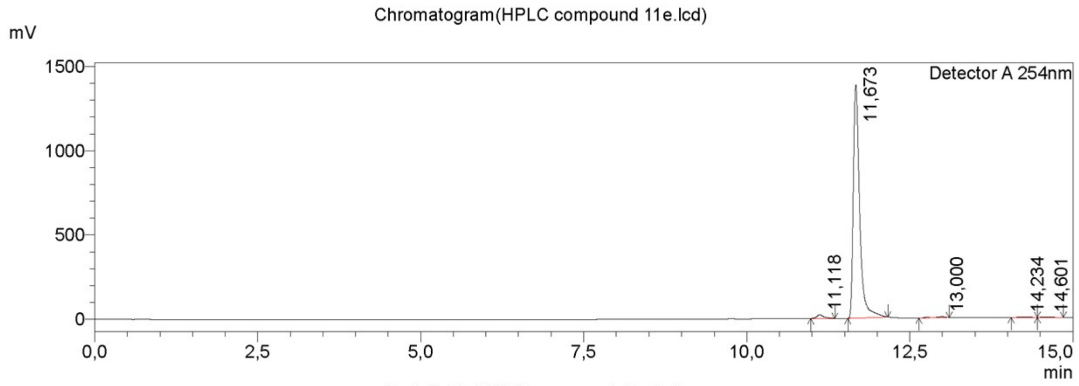

Peak Table(HPLC compound 11e.lcd)

Detector A 254nm

Peak#	Ret. Time	Area	Height	Mark	Conc.	Area%
1	11,118	159107	21682	M	1,638	1,638
2	11,673	9413281	1384051	M	96,905	96,905
3	13,000	76263	6118	M	0,785	0,785
4	14,234	31478	3199	M	0,324	0,324
5	14,601	33769	3856	M	0,348	0,348
Total		9713899	1418906		100,000	100,000

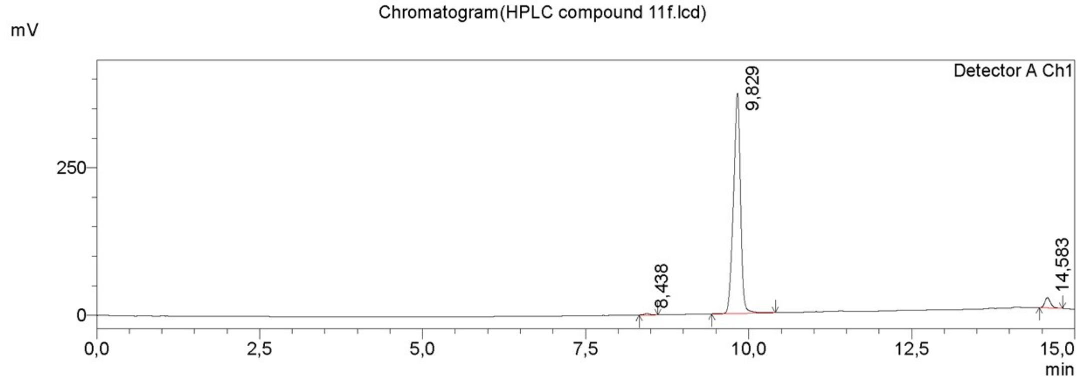

Peak Table(HPLC compound 11f.lcd)

Detector A 254nm

Peak#	Ret. Time	Area	Height	Mark	Conc.	Area%
1	8,438	12599	2222	M	0,431	0,431
2	9,829	2803777	372918	M	95,937	95,937
3	14,583	106158	17566	M	3,632	3,632
Total		2922533	392706		100,000	100,000

Classified as Internal | Intern

==== Shimadzu LabSolutions Browser Report ====

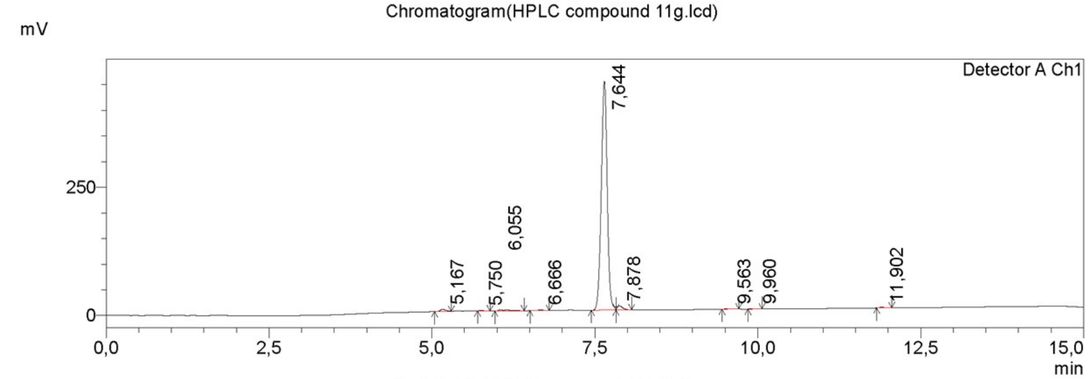

Peak Table(HPLC compound 11g.lcd)

Detector A 254nm

Peak#	Ret. Time	Area	Height	Mark	Conc.	Area%
1	5,167	24462	4387	M	0,832	0,832
2	5,750	3852	751	M	0,131	0,131
3	6,055	26790	2097	M	0,911	0,911
4	6,666	8618	1365	M	0,293	0,293
5	7,644	2814064	446331	M	95,729	95,729
6	7,878	43056	7178	V M	1,465	1,465
7	9,563	9087	1221	M	0,309	0,309
8	9,960	3623	553	M	0,123	0,123
9	11,902	6074	1112	M	0,207	0,207
Total		2939627	464996		100,000	100,000

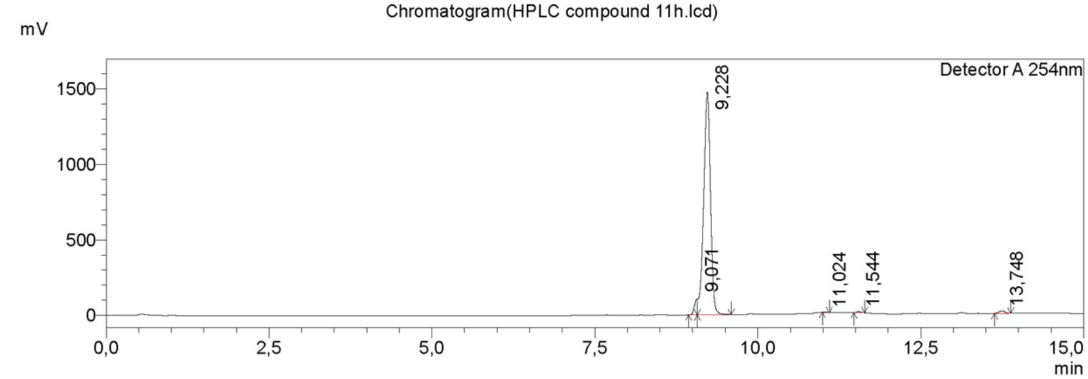

Peak Table(HPLC compound 11h.lcd)

Detector A 254nm

Peak#	Ret. Time	Area	Height	Mark	Conc.	Area%
1	9,071	352982	103304	M	3,125	3,125
2	9,228	10778651	1475223	V M	95,422	95,422
3	11,024	11950	3624	M	0,106	0,106
4	11,544	42357	7806	M	0,375	0,375
5	13,748	109887	16604	M	0,973	0,973
Total		11295827	1606562		100,000	100,000

Classified as Internal | Intern

==== Shimadzu LabSolutions Browser Report ====

Chromatogram(HPLC compound 11i.lcd)

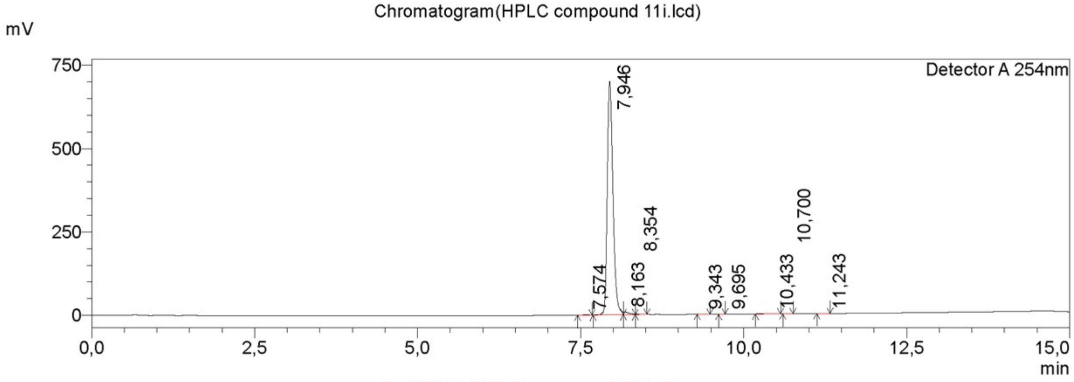

Peak Table(HPLC compound 11i.lcd)

Detector A 254nm

Peak#	Ret. Time	Area	Height	Mark	Conc.	Area%
1	7,574	6327	1251	M	0,146	0,146
2	7,946	4257841	701045	M	98,310	98,310
3	8,163	45023	8144	V M	1,040	1,040
4	8,354	5690	1021	V M	0,131	0,131
5	9,343	1159	188	M	0,027	0,027
6	9,695	676	139	M	0,016	0,016
7	10,433	10017	628	M	0,231	0,231
8	10,700	2168	465	M	0,050	0,050
9	11,243	2126	381	M	0,049	0,049
Total		4331028	713262		100,000	100,000

Chromatogram(HPLC compound 11j.lcd)

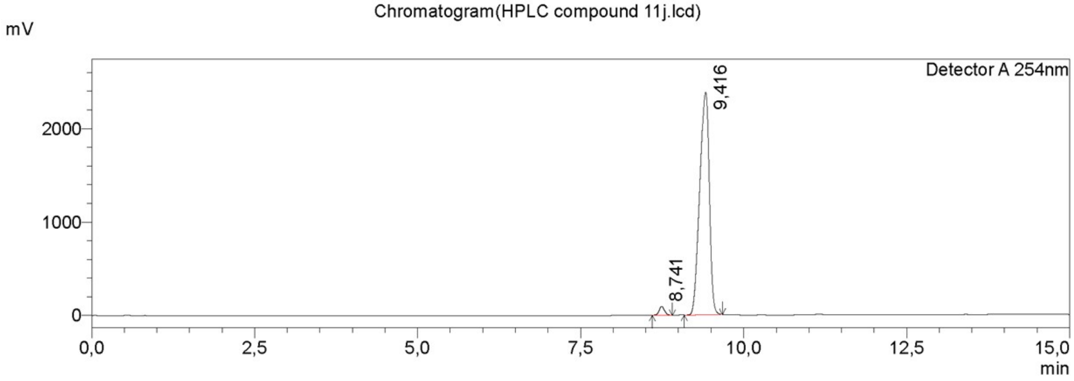

Peak Table(HPLC compound 11j.lcd)

Detector A 254nm

Peak#	Ret. Time	Area	Height	Mark	Conc.	Area%
1	8,741	556177	94727	M	2,264	2,264
2	9,416	24013718	2381408	M	97,736	97,736
Total		24569895	2476135		100,000	100,000

Classified as Internal | Intern

==== Shimadzu LabSolutions Browser Report ====

Chromatogram(HPLC compound 11k.lcd)

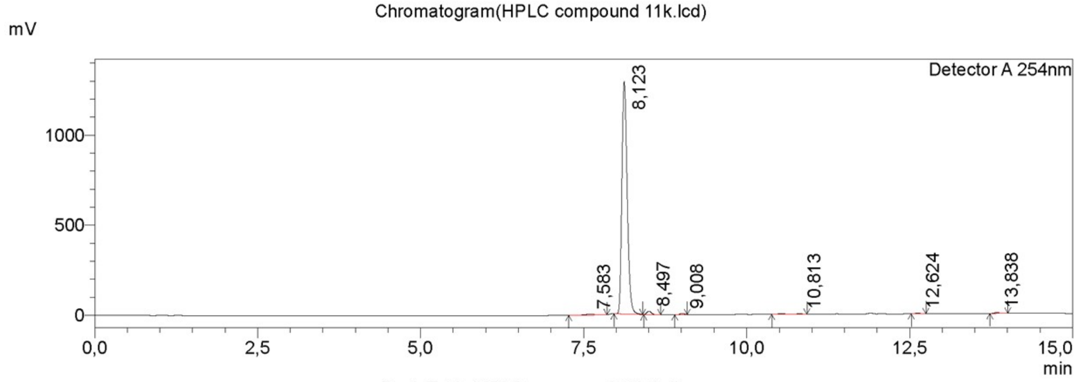

Peak Table(HPLC compound 11k.lcd)

Detector A 254nm

Peak#	Ret. Time	Area	Height	Mark	Conc.	Area%
1	7,583	57308	5774	M	0,752	0,752
2	8,123	7310019	1293201	M	95,872	95,872
3	8,497	96765	19262	M	1,269	1,269
4	9,008	36129	7794	M	0,474	0,474
5	10,813	72363	4992	M	0,949	0,949
6	12,624	25716	5243	M	0,337	0,337
7	13,838	26478	4950	M	0,347	0,347
Total		7624778	1341216		100,000	100,000

Chromatogram(HPLC compound 11l.lcd)

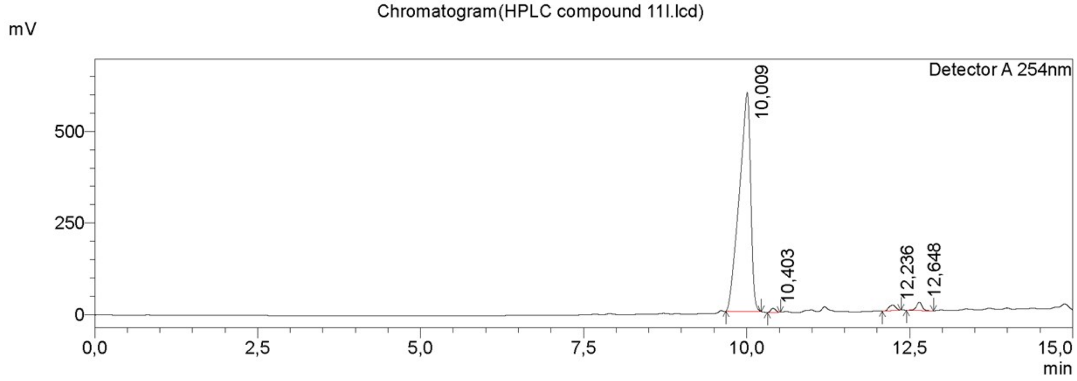

Peak Table(HPLC compound 11l.lcd)

Detector A 254nm

Peak#	Ret. Time	Area	Height	Mark	Conc.	Area%
1	10,009	7237072	597217	M	95,979	95,979
2	10,403	55329	10832	M	0,734	0,734
3	12,236	108856	15326	M	1,444	1,444
4	12,648	139020	22954	M	1,844	1,844
Total		7540277	646330		100,000	100,000

Classified as Internal | Intern

==== Shimadzu LabSolutions Browser Report ====

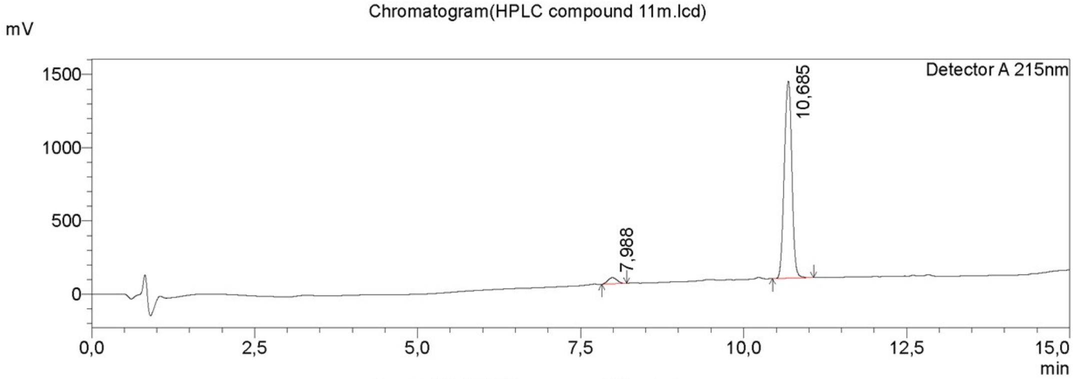

Peak Table(HPLC compound 11m.lcd)

Detector A 215nm

Peak#	Ret. Time	Area	Height	Mark	Conc.	Area%
1	7,988	420448	42782	M	3,716	3,716
2	10,685	10895489	1346425	M	96,284	96,284
Total		11315937	1389207		100,000	100,000

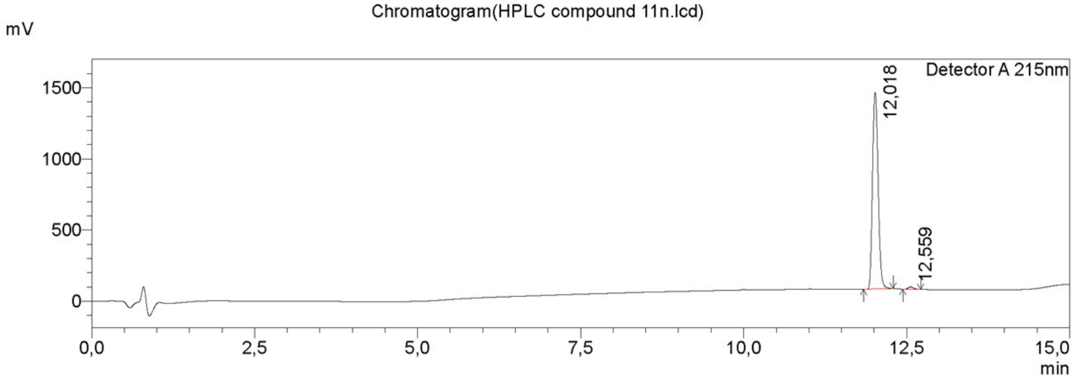

Peak Table(HPLC compound 11n.lcd)

Detector A 215nm

Peak#	Ret. Time	Area	Height	Mark	Conc.	Area%
1	12,018	8306570	1382774	M	98,874	98,874
2	12,559	94604	17008	M	1,126	1,126
Total		8401174	1399782		100,000	100,000

Classified as Internal | Intern

==== Shimadzu LabSolutions Browser Report ====

Chromatogram(HPLC compound 11o.lcd)

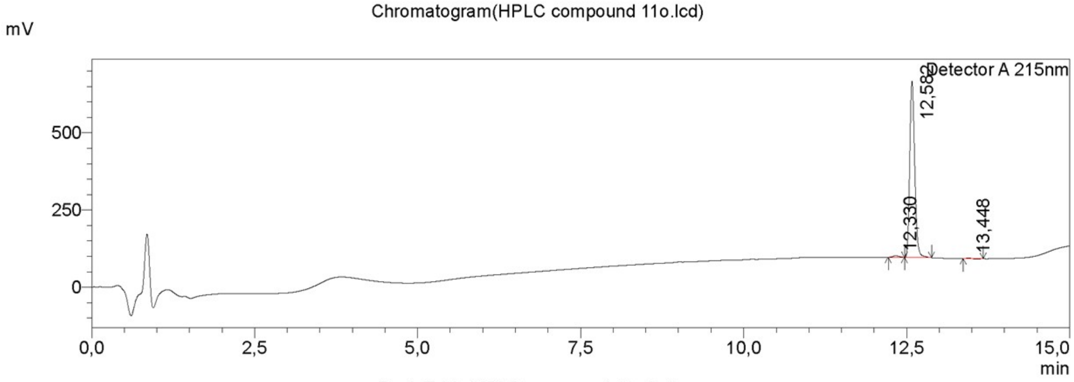

Peak Table(HPLC compound 11o.lcd)

Detector A 215nm

Peak#	Ret. Time	Area	Height	Mark	Conc.	Area%
1	12,330	31502	4708	M	1,024	1,024
2	12,582	3039719	570937	M	98,816	98,816
3	13,448	4929	2189	M	0,160	0,160
Total		3076150	577834		100,000	100,000

Chromatogram(HPLC compound 11p.lcd)

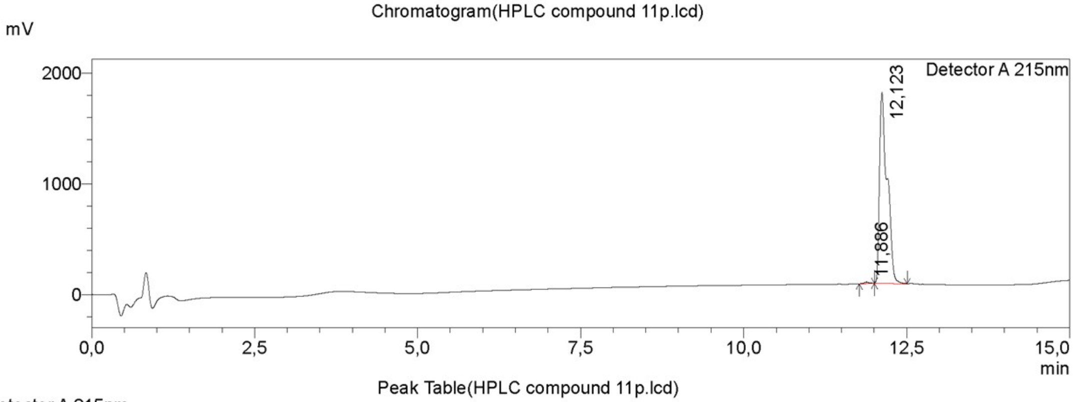

Peak Table(HPLC compound 11p.lcd)

Detector A 215nm

Peak#	Ret. Time	Area	Height	Mark	Conc.	Area%
1	11,886	87322	14920	M	0,621	0,621
2	12,123	13970099	1727412	M	99,379	99,379
Total		14057421	1742332		100,000	100,000

Classified as Internal | Intern
